# Two atoms at once: ligand skeleton expansion by a –CN– unit *via* nitrile insertion into the pyridinium amidate backbone of platinum complexes

**DOI:** 10.1039/d6dt02010k

**Published:** 2026-09-04

**Authors:** Lars K. Burri, Svitlana Shishkina, Martin Albrecht

**Affiliations:** a Department of Chemistry, Biochemistry, and Pharmaceutical Sciences, University of Bern Freiestrasse 3 CH-3012 Bern Switzerland martin.albrecht@unibe.ch; b Department of Organophosphorus Chemistry, Institute of Organic Chemistry Academician Kukhar str. 5 02094 Kyiv-94 Ukraine

## Abstract

We demonstrate the selective and direct platinum-mediated insertion of a nitrile fragment into the C–N bond of a pyridinium amidate scaffold. This reactivity expands the ligand skeleton through the insertion of two atoms at once. Isotope labelling indicates N–C_pyridinium_ bond cleavage in the ligand, and time-dependent monitoring suggests the formation of multiple intermediates *en route* to the final nitrile-inserted complex.

## Abstract 2

Platinum-mediated insertion of a nitrile fragment into the C–N bond of a pyridinium amidate scaffold enables selective expansion of the ligand skeleton by two atoms at once, in contrast to the typically observed single C or N insertion processes.

## Graphical abstract

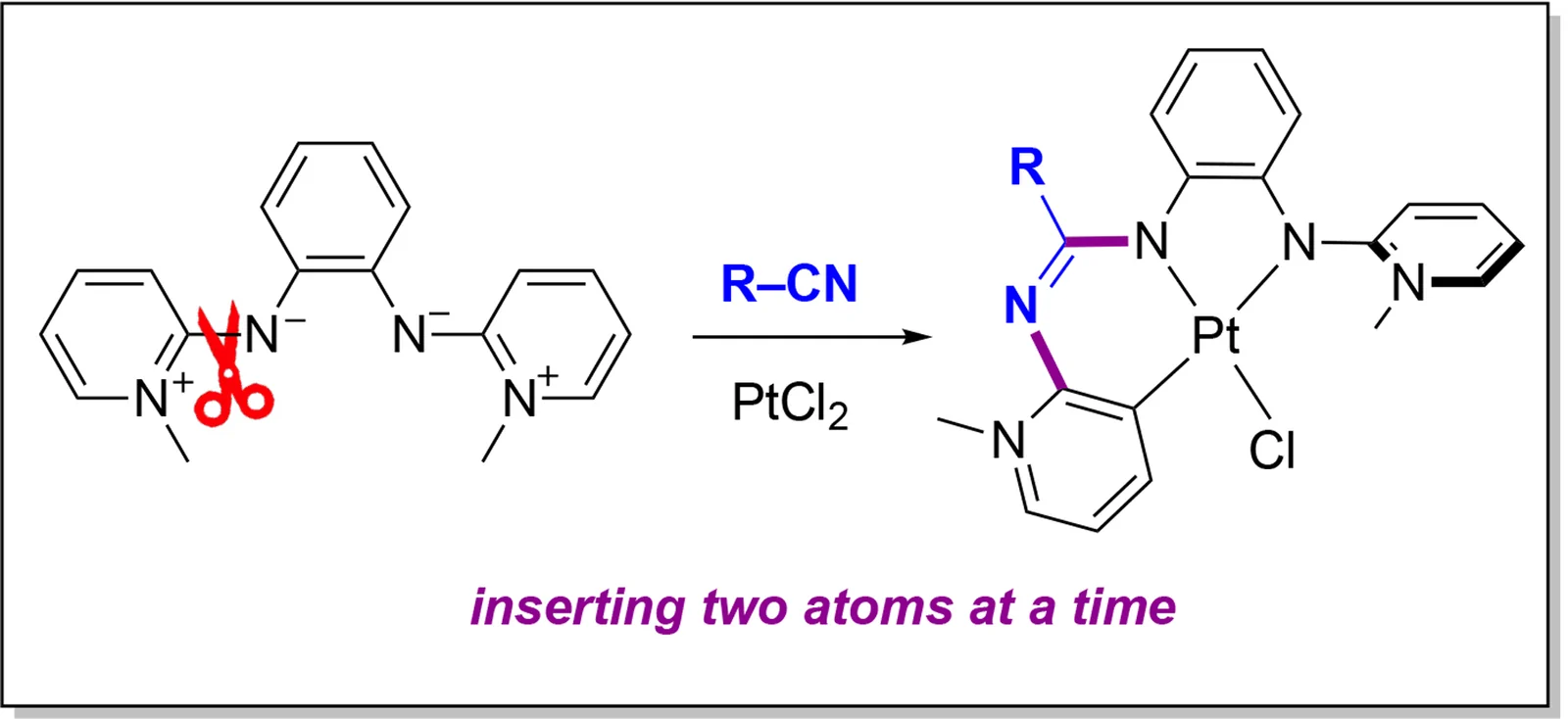

Skeletal editing^1^ has become a powerful and increasingly popular tool to access novel molecular structures without the need to develop completely new synthetic routes.^2–4^ Most potent strategies include the editing of (hetero)cyclic skeletons, including atom deletion,^5,6^ transmutation^7–10^ and insertion,^11–14^ though acyclic skeleton editing has also become available.^15,16^ Editing typically involves single carbon or nitrogen atom manipulation, promoted by the progress in carbene and nitrene chemistry. In contrast, insertion of two or more atoms into a cyclic structure is much less common and largely limited to ring opening and reconnections.^17–20^ The selective insertion of a –CN– fragment is unprecedented and may offer a new approach for altering and potentially functionalizing a wide array of (hetero)cyclic compounds. A recent procedure utilized nitriles together with boryl radicals for –CN– insertion yet remains limited to trifluoroacetamides as substrates.^21^

Here, we show that pyridinium amidates (PYAs) are prone to the insertion of a –CN– fragment from nitriles such as acetonitrile or benzonitrile in a platinum-mediated activation of a C–N bond of the PYA skeleton. Such PYAs have become particularly versatile in catalytic applications, in part because of their electronic flexibility within the continuum between an imine-type quinoidal limiting resonance structure and a zwitterionic resonance structure with anionic amidate donors (Scheme 1).^22,23^ However, in palladium chemistry, metalation of the phenylene-bridged bisPYA ligand 1 produces the expected coordination complexes of type [Pd(N,N)X_2_] (with N,N = 1),^24^ the same reaction with platinum induces unprecedented C–N bond cleavage and selective direct nitrile insertion into the backbone of one of the PYA units, allowing the PYA backbone to be expanded by two atoms.

**Scheme 1:**
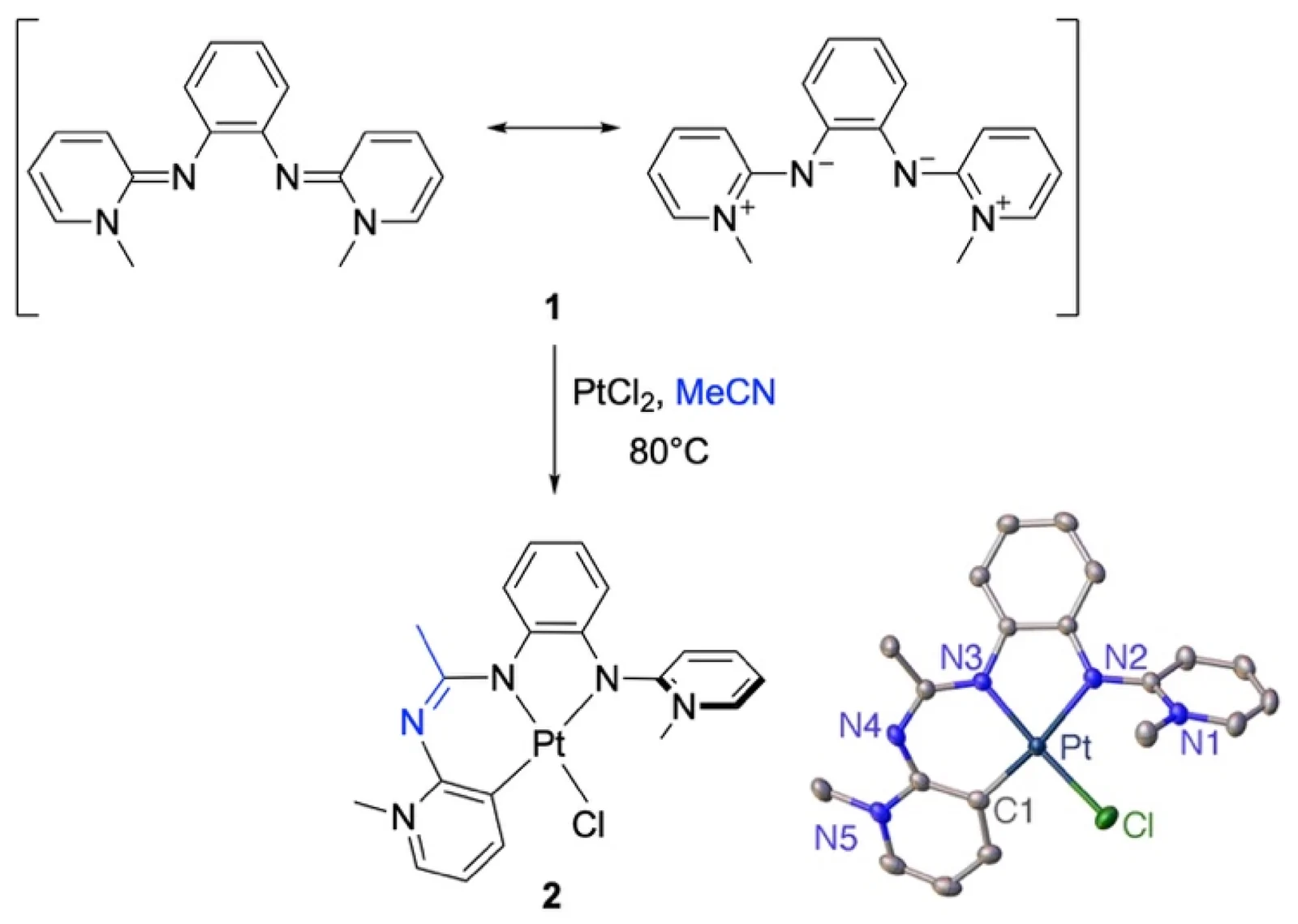
Insertion of MeCN into the C–N bond of ligand 1 mediated by platinum to form complex 2, and the molecular structure of 2 determined by X-ray diffraction.

Specifically, the reaction of bisPYA ligand 1 with [PtCl_2_(MeCN)_2_] in acetonitrile resulted in the formation of a new species together with the protonated ligand in a 1 : 1 ratio according to ^1^H NMR spectroscopy. Purification of the reaction mixture over a short Al_2_O_3_ plug afforded complex 2 as a red and air-stable solid in 44% yield (Scheme 1). Recrystallization of 2 by diffusion of pentane into a CH_2_Cl_2_ solution of the complex afforded crystals suitable for X-ray diffraction analysis. The molecular structure revealed a square-planar platinum center with an N,N′,C-tridentate coordinating ligand. The ligand has been expanded by a CN unit, which was identified by the additional methyl group as the product of insertion of MeCN into the backbone of the PYA ligand. Such a product implies C–N cleavage of the ligand and formation of a new N–C_MeCN_ as well as a new C–N_MeCN_ bond. Contrary to the typically observed reaction trajectories for metal-mediated nitrile activation,^25,26^ the process here involves direct and selective C–N bond formation with both the nitrogen and carbon of the nitrile. Presumably as a consequence of the expansion of the ligand scaffold, cyclometalation occurred *via* activation of the C(sp^2^)–H bond of the pyridinium ring. Notably, cyclometallation of PYA ligands has thus far only been observed *via* C(sp^3^)–H bond activation of the *N*-bound methyl group,^24^ while formation of 2 marks the first example of C(sp^2^)–H bond activation with PYA ligand systems.

The ^1^H NMR spectroscopic data for complex 2 are in agreement with the X-ray diffraction analysis. Specifically, the spectrum features three distinct methyl resonances at 2.44 ppm corresponding to the *C*-bound CH_3_ unit from acetonitrile insertion, and at 3.95 and 4.13 ppm for the two inequivalent pyridinium N–CH_3_ groups. Eleven distinct aromatic resonances indicate the dissymmetric nature of the ligand as well as cyclometalation, the latter further supported by a low-field doublet at 8.51 ppm with characteristic satellites due to ^195^Pt coupling (^2^*J*_PtH_ = 7.4 Hz).

The CN insertion reaction is highly sensitive to temperature and additives. When performed at 80 °C, the reaction is complete within 5 h, while at 60 °C, about 3 days are required to reach completion, yet product distribution in the crude mixture remained at a 1 : 1 ratio, suggesting that this product distribution is inherent to the transformation and not a kinetic result. The reaction also proceeds smoothly from PtCl_2_, though pre-stirring in MeCN to form [PtCl_2_(MeCN)_2_] is essential, while addition of 1 together with PtCl_2_ in MeCN resulted in the formation of several side products and a significantly lower yield of the cyclometalated complex 2. Furthermore, the reaction is sensitive to water. Using wet MeCN containing 2.5% v/v H_2_O completely inhibited the formation of 2, suggesting that proton transfer interferes with one of the intermediate species.

Nitrile insertion and formation of 2 imply either phenylene C–N bond breaking or pyridinium C–N bond cleavage. To distinguish between these two processes, the reaction was performed in ^15^N-labeled acetonitrile.††Due to the low amount of solvent available, this reaction was performed as a one-pot reaction resulting in two isomers after alox purification. The ^1^H NMR shifts of the cyclometalated product 2-^15^N were identical to those of the unlabeled analogue, while ^15^N NMR spectroscopy did not allow for identification of a well-resolved resonance. However, two-dimensional ^1^H–^15^N HMBC NMR spectroscopy unequivocally revealed three cross-peaks at *δ*_N_ = 220 ppm (Fig. 1). The most intense coupling of the ^15^N nucleus is with the *C*-bound methyl group originating from the inserted MeCN equivalent, which also appears as a doublet in the ^1^H NMR spectrum (*J*_NH_ = 3.4 Hz; H_α_ in Fig. 1). A less intense coupling was observed with the pyridinium N–CH_3_ group at *δ*_H_ = 3.95 ppm (H_β_), and a weak coupling was also observed with the C6-bound hydrogen of the pyridinium unit at *δ*_H_ = 7.5 ppm (H_γ_). This coupling pattern identifies the location of the ^15^N nucleus as bound to the pyridinium heterocycle with a strong ^3^*J*_NH_ and two weaker ^4^*J*_NH_ couplings rather than to the phenylene ring. Accordingly, the phenylene diamine backbone of the ligand is robust, and nitrile insertion occurs through scission of the exocyclic C_pyridinium_–N bond. Mechanistically, this outcome may speculatively point to a nucleophilic aromatic substitution-type trajectory involving MeCN as the nucleophile reacting with the electron-poor pyridinium heterocycle. Clearly, the platinum center must play a crucial role, as no such activity was observed with other metals such as Pd.^27^

**Fig. 1:**
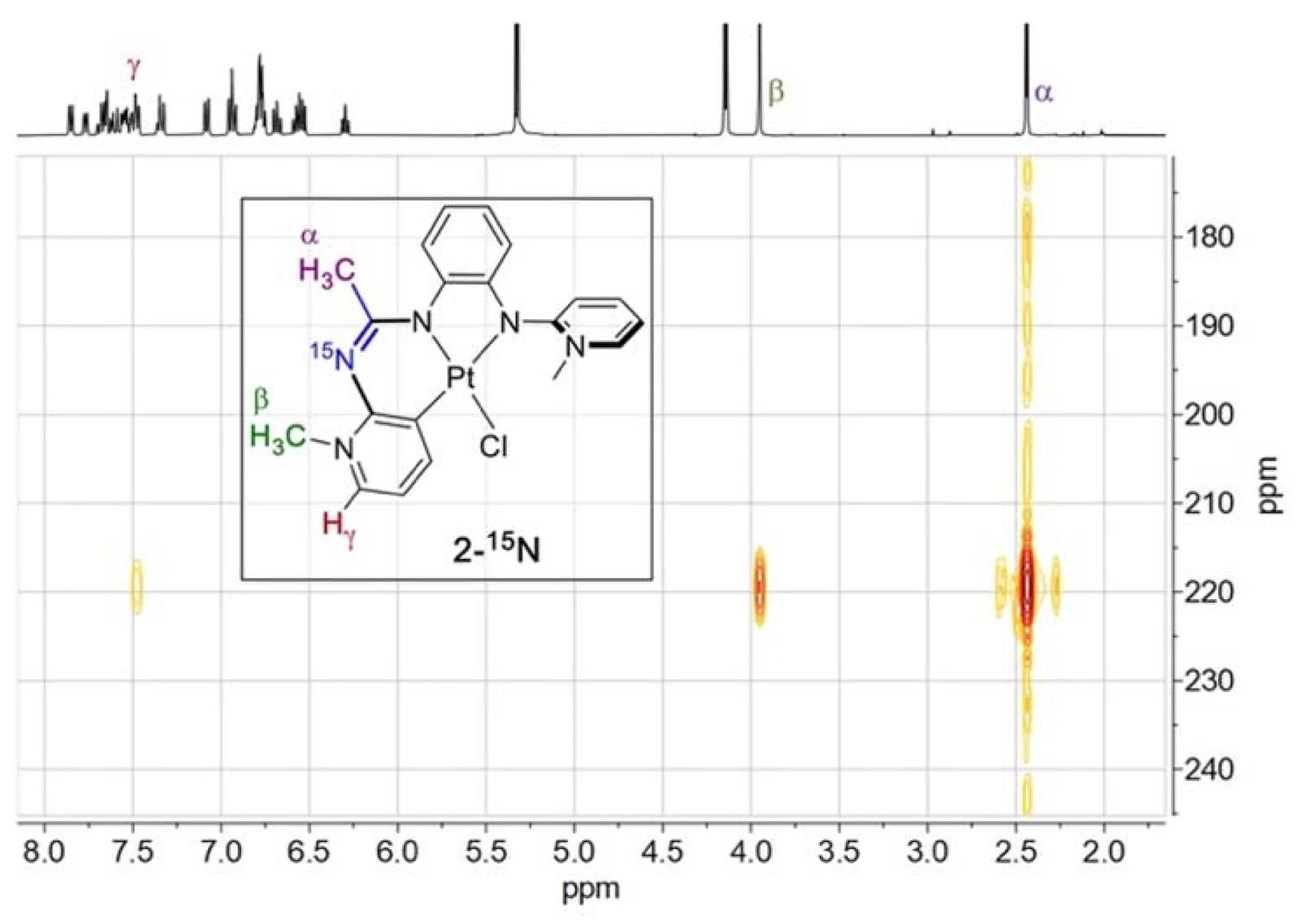
^1^H–^15^N HMBC spectrum of 2-^15^N in CD_2_Cl_2_.

To gain a better understanding of this unprecedented nitrile insertion, the reaction was performed in CD_3_CN and monitored by ^1^H NMR spectroscopy at both 60 and 75 °C. At 60 °C, two sets of transient methyl signals were observed in the pyridinium methyl region (4.5–3.0 ppm). The set of 4 methyl signals forms first, immediately after the start of the reaction, and was attributed to a first intermediate (A). A second species consisting of two signals is observed to accumulate a few minutes later and was attributed to a second intermediate (B). Structural analysis of these intermediates was precluded by the massive overlap of almost all aromatic proton signals as well as the persistently low concentrations of both species at any stage of the reaction. The four methyl signals of the first intermediate (A) were attributed to the formation of two isomers based on their simultaneous and proportional formation and consumption. The relative ratio of these putative isomers was temperature-dependent. At 60 °C, the ratio of the two species is essentially identical, while at 75 °C, a 2 : 3 ratio was observed based on methyl signal integrations. Both the first and second intermediates are expected to be dissymmetric as they feature two distinct methyl signals in the NMR spectrum, similar to the final species 2. The initial free ligand resonance shifted drastically from 3.25 ppm at the start to 3.5 ppm, while also becoming broader over time. This change is likely caused by an equilibrium of the ligand with its protonated analogue 1·HX (see below).

Kinetic modelling of the concentrations of the observed species fitted very well phenomenologically with a consecutive rate law (Fig. 2), where an initial step is first order in ligand to produce the first intermediate (*k*_1_) and subsequently the second intermediate B (*k*_2_), and eventually complex 2 (*k*_3_). To account for the additional depletion of the ligand through the equilibrium identified by the chemical shift changes (see above) as well as some other minor side products, a competitive reaction of the free ligand with the rate constant *k*_4_ was included. The resulting concentrations were modeled with SciPy^28^ and reproduced the experimental data points with *k*_1_ = 10^−4.1^ s^−1^, *k*_2_ = 10^−3.3^ s^−1^, *k*_3_ = 10^−2.9^ s^−1^ and *k*_4_ = 10^−3.9^ s^−1^ (Fig. 2, Tables S1–S7). The rate constants *k*_2_ and *k*_3_ are about one order of magnitude higher than *k*_1_ and suggest considerably faster consecutive reactions compared to the first process to form intermediate A. The three distinct rates point to a complex process as may be expected for the multiple bond breaking and making events leading to net nitrile insertion. The rate of the combined side reactions *k*_4_ is slightly larger than and obviously correlated with *k*_1_, resulting in a modeled total yield of 39% for the cyclometalated product 2, which is similar to the 44% isolated yield measured for the reaction on an NMR scale. The rates at 75 °C were calculated to be *k*_1_ = 10^−3.6^ s^−1^, *k*_2_ = 10^−2.8^ s^−1^, *k*_3_ = 10^−2.2^ s^−1^ and *k*_4_ = 10^−3.2^ s^−1^, which is around a factor of 3 faster than the *k*_1_, *k*_2_, and *k*_3_ rates at 60 °C.

**Fig. 2:**
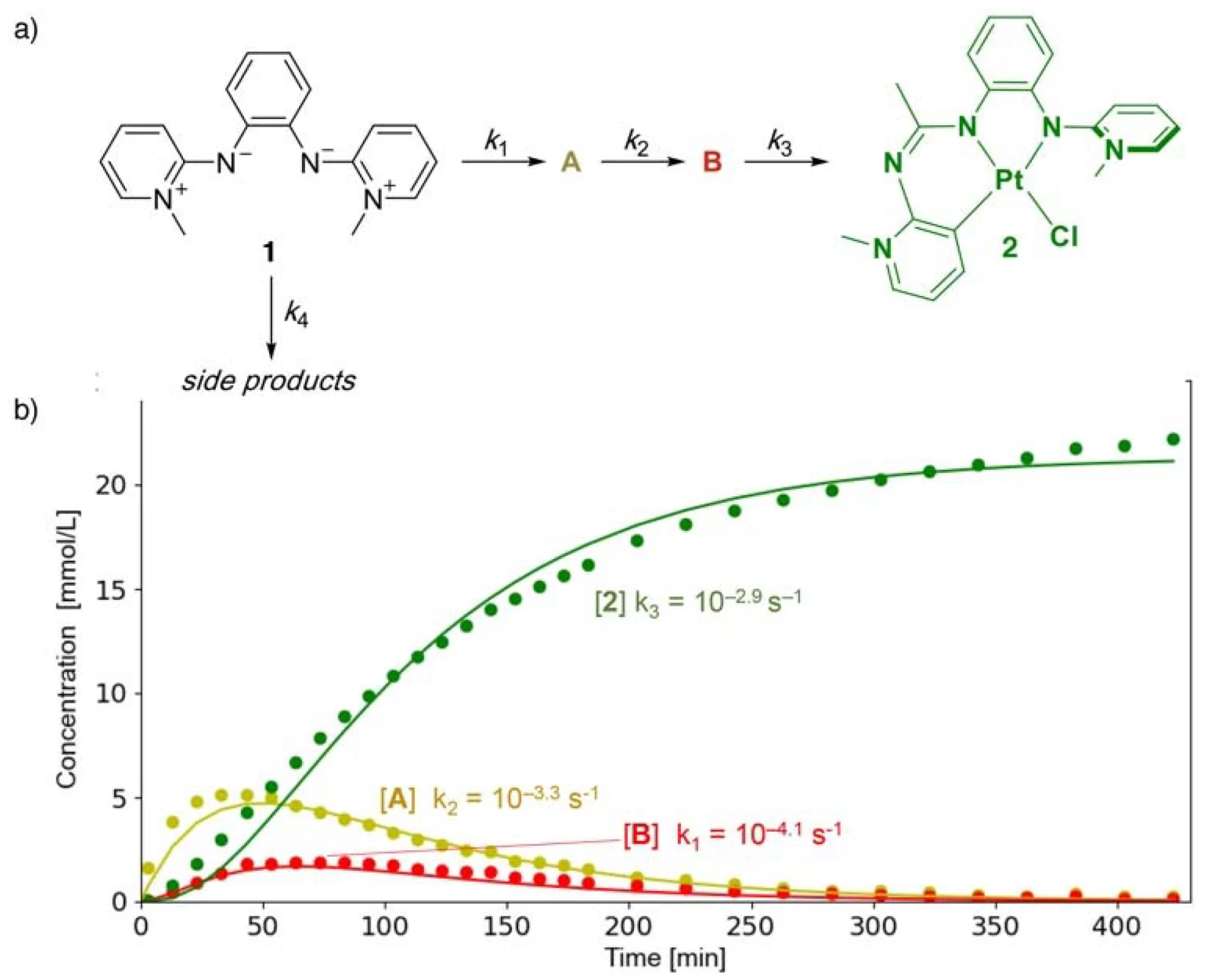
(a) Reaction scheme used for modeling the formation of 2 through sequential conversion of 1*via* a series of consecutive reactions involving two intermediates A and B as well as a parasitic side reaction of 1; (b) concentrations of the putative intermediates A (yellow), B (red) and the cyclometalated product 2 (green) over time at 60 °C; measured data points are represented by filled circles, and modeled time-dependent concentrations based on first-order rate constants are shown as solid lines.

The major by-product of the reaction was identified as the protonated ligand 1, presumably formed by scavenging the HCl released from the cyclometallation step *en route* to complex 2. Formation of the protonated ligand induces an equilibrium between residual 1 and 1·HX, in agreement with the gradual shift of the N–CH_3_ resonances observed during the reaction (see above). Consistent with this model, addition of free ligand 1 to the final product mixture shifted all signals of the by-product towards the frequencies of the free ligand. Similarly, addition of excess KO*t*Bu to the final product mixture (Fig. S9) shifted the resonances of the by-product to exactly match those of the free ligand 1. These results indicate the dual role of compound 1 in (i) coordinating to platinum and (ii) acting as a base towards the formed HCl. According to such a model, protonation of the ligand also restricts the nitrile insertion reaction from reaching higher yields. Attempts to increase the yield of 2 included the use of an external base. However, addition of either KO*t*Bu or KHMDS (HMDS = N(SiMe_3_)_2_^−^) suppressed the formation of complex 2, and only free ligand 1 was recovered after 18 h at 60 °C.‡‡A color change was observed upon the addition of the base to PtCl_2_(NCMe)_2_, suggesting base coordination to the metal, which is assumed to deviate the reaction coordinate. Using a weaker non-coordinating base such as NEtiPr_2_ or a proton sponge significantly decreased the yield of complex 2 and resulted in the formation of several side products. We therefore reverted to ligand 1 as the base. When using 2 equiv. of ligand, the spectroscopic yield of complex 2 indeed increased to 67% (based on Pt), suggesting that yield optimization is feasible through careful choice of base.

The reaction was successfully expanded to the insertion of other nitriles (Scheme 2). Thus, reaction of PtCl_2_ and ligand 1 with propionitrile or benzonitrile yielded the nitrile insertion products 3 and 4. The yield of the cyclometalated species was lowered with the larger steric bulk of the nitrile and decreased from 50% with MeCN to 42% with EtCN and further to 36% with PhCN. The identity of both complexes 3 and 4 was confirmed by X-ray diffraction analysis.

**Scheme 2:**
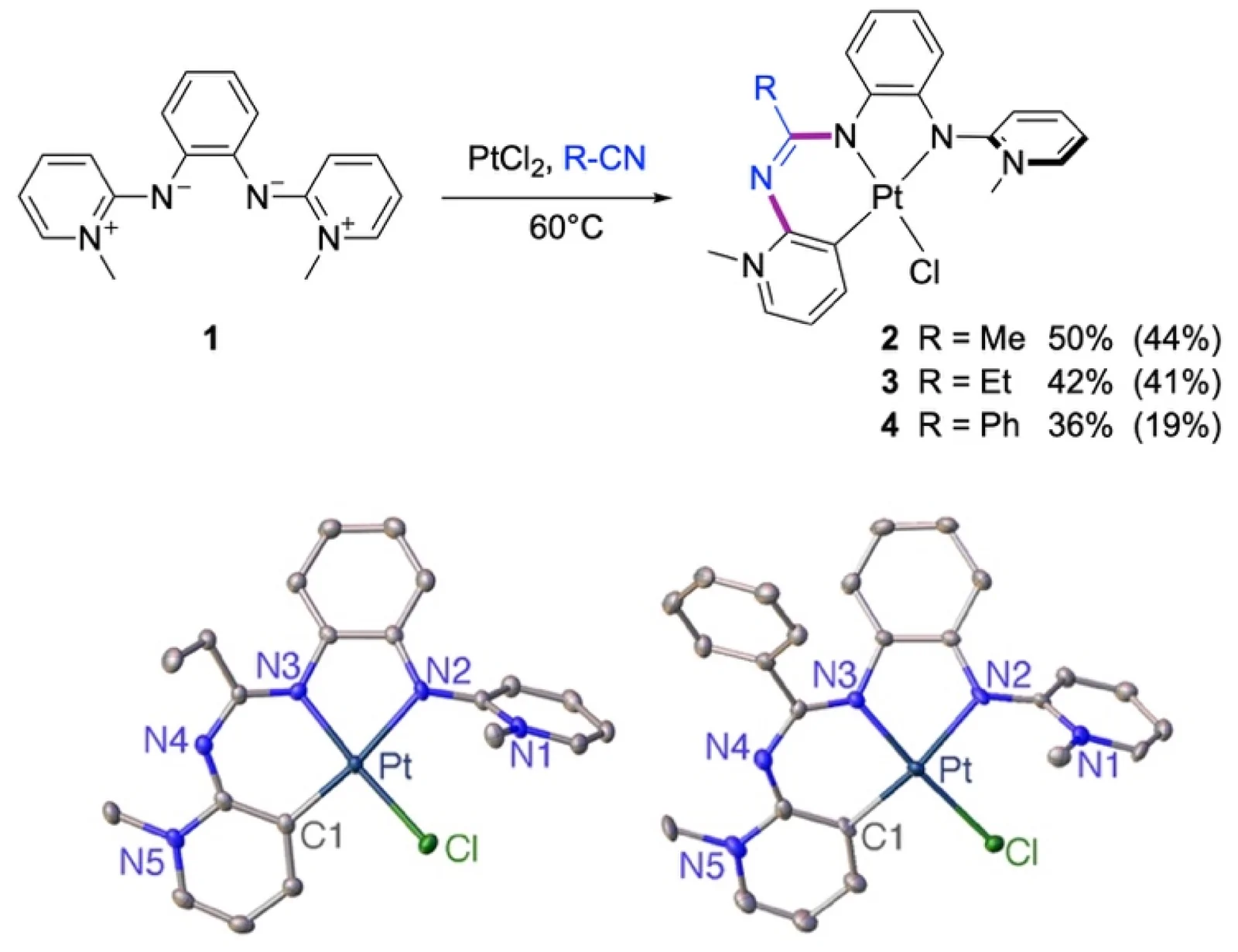
Ligand skeleton expansion of bisPYA 1 with different nitriles with spectroscopic yields (isolated yields in parentheses) and crystallographically determined molecular structures of 3 and 4 (selected bond metrics are given in Tables S10 and S11).

In an attempt to use the platinum center only as a transient template, we investigated the demetallation of complex 2. However, the C,N,N-tridentate ligand coordination imparted remarkable stability under forcing conditions, including aqueous HCl (6 M, 25 °C, 18 h), methanolic NaCN at 60 °C, and aqueous NaOH (2 M). Applying more forcing conditions, such as aqua regia or NaCN at 75 °C for 3 days, decomposed the ligand, with multiple indistinguishable resonances appearing in the ^1^H NMR spectrum, which may point to a high potential of the ligand to hydrolyze when removed from the coordination sphere of the platinum center.

## Conclusions

We observed C–N bond cleavage and acetonitrile insertion into the backbone of a pyridinium amidate ligand through the formation of a new C–N bond as well as a new N–C bond. The nitrile-inserted cyclometalated product was characterized by NMR spectroscopy and XRD and obtained in 50% spectroscopic yield, but further conversion is likely hindered by the formation of the protonated ligand. ^15^N NMR studies revealed that cleavage of the exocyclic C_pyridinium_–N bond takes place in this nitrile insertion reaction rather than C_phenylene_–N bond scission. Kinetic studies suggest that the reaction proceeds through two dissymmetric intermediates, with one of them consisting of two isomers. The reaction also proceeds with propionitrile and benzonitrile. However, the yield is slightly reduced with bulkier groups. This unusual insertion reaction provides a direct way to cleave a C–N bond and selectively insert different nitriles in between. A further understanding of the mechanism and expansion of the reaction to other substrates may offer a methodology for late-stage and modular ligand diversification.

## Author contributions

L. K. B performed all experimental work and data analysis; S. S. performed all crystallographic analyses; M. A. supervised the work; and L. K. B. and M. A. conceptualized the work, analysed the data and wrote the manuscript.

## Conflicts of interest

There are no conflicts to declare.

## Supplementary Material

DT-055-D6DT02010K-s001

## Data Availability

Supplementary information (SI): synthetic procedures, kinetic data, analytical data and spectra of all new compounds, as well as crystallographic details. See DOI: https://doi.org/10.1039/d6dt02010k. CCDC 2571090–2571092 contain the supplementary crystallographic data for this paper.^29*a*–*c*^
