# Supplementary material for: Two atoms at once: ligand skeleton expansion by a –CN– unit via nitrile insertion into the pyridinium amidate backbone of platinum complexes
Source: Dalton Trans. 2026 Sep 4;55(37):13381–5. doi: 10.1039/d6dt02010k (PMC13580393; doi:10.1039/d6dt02010k)
Supplement: Supplementary file 1 [file DT-055-D6DT02010K-s001.pdf]

Supplementary Information (SI) for Dalton Transactions.
This journal is © The Royal Society of Chemistry

Electronic Supporting Information

belonging to

Two atoms at once: Ligand skeleton expansion by a –CN– unit via nitrile insertion into the pyridinium amidate backbone of platinum complexes

Lars K. Burri,[a] Svitlana Shishkina,[b] and Martin Albrecht *[a]

a) *Department of Chemistry, Biochemistry, and Pharmaceutical Sciences, University of Bern, Freiestrasse 3, CH-3012 Bern, Switzerland*

b) Department of Organophosphorus Chemistry, Institute of Organic Chemistry, Academician Kukhar str. 5, 02094, Kyiv-94*, Ukraine*

Email: martin.albrecht@unibe.ch

Table of Contents

1. Synthetic procedures S2
1.1 General information S2
1.2 Synthesis of ligand and complexes S2
2. ^{15}N labeling experiment S5
3. Kinetic experiments S6
3.1 Temperature-dependent reactions S6
3.2 Continuous reaction monitoring at 60 °C and 75 °C S8
4. Synthesis of Complex **2** with 2 equiv. of bisPYA ligand S16
5. X-Ray Crystallographic Data S18
6. NMR Spectra S22
7. References S27
Appendix: full codes and statistical analysis of kinetic fitting of the reaction at 60 °C, at 75 °C considering the two putative isomers of **A** as a single species (termed "intermediate 1 combined"), and as two isomers **A'** and **A''** (termed "intermediate 1 as two isomers")

1. Synthetic procedures

1.1 General information

All reactions were carried out under an inert atmosphere of argon or nitrogen using standard Schlenk techniques unless stated otherwise. Purifications were carried out in air. 2-chloro-1-methylpyridinium triflate was prepared according to a literature procedure.[S1] CH_2Cl_2 and MeCN were dried by passage through a solvent purification column. CD_3CN, PhCN, and EtCN were sparged with nitrogen gas and dried over 3 Å molecular sieves. All other reactants and non-dry solvents were obtained from common commercial suppliers and used without further purification.

NMR spectra were recorded at 25 °C on Bruker spectrometers operating at 300 or 400 MHz (^{1}H NMR) and 75 or 100 MHz (^{13}C{^{1}H} NMR), unless specified otherwise. Chemical shifts (δ) are expressed in ppm downfield from $SiMe_4$ and referenced by the residual protonated solvent. Atoms were assigned based on homo- and heteronuclear shift correlation spectroscopy as well as nuclear Overhauser effect (NOE) measurements. For ^{1}H-^{15}N HMBC measurements, the hmbcgpndqf pulse sequence was used with spectral widths F1 = 4854.4 Hz and F2 = 24342.7 Hz, 16 scans and 1.0 s relaxation delay. High resolution mass spectrometry was carried out by the mass spectroscopy service of the DCBP using a Thermo Scientific LTQ Orbitrap XL (ESI-Orbitrap) as a spectrometer. Elemental analysis was performed by the microanalytic laboratory of the DCBP using a Thermo Scientific Flash 2000 CHNS-O elemental analyzer.

1.2 Synthesis of ligand and complexes

Phenylene bis(pyridinium amidate) (1)

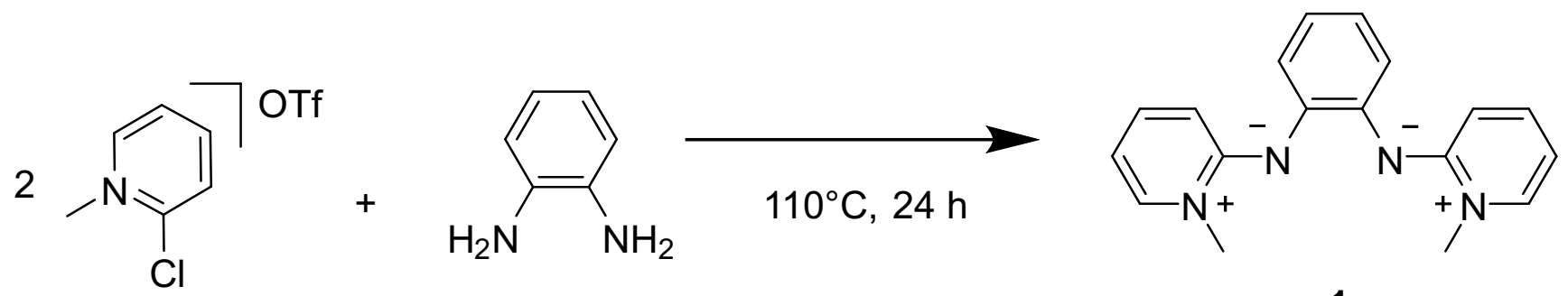

The title compound was prepared similarly to a literature procedure.[S2] Accordingly, 2-chloro-1-methylpyridinium triflate (2.00 g, 7.2 mmol) and 1,2-diamino-benzene (389 mg, 3.6 mmol) were added to a microwave vial. Anhydrous NEt_3 (1.5 mL, 10.8 mmol) and dry MeCN (10 mL) were added. The mixture was stirred at 110 °C for 24 h. The solution was cooled to RT, dried in vacuo and the solid washed with Et_2O (2 x 20 mL). The solid was dried again and then HCl_{aq} (6 M, 2 mL) was added. The mixture was stirred and a solution of NH_4PF_6 (4.7 g, 29 mmol) dissolved in a minium amount of H_2O was added. The mixture was stirred for 15 min, during which an off-white solid precipitated. The mixture was filtered and the solid was washed with ice-cold H_2O (2 x 20 mL) and then with Et_2O (20 mL). The solid was thoroughly dried in vacuo and then stirred with $NaOH_{aq}$ (2 M, 40 mL) for 15 min. A yellow solid precipitated almost immediately. The mixture was extracted with CH_2Cl_2 (3 x 20 mL). The combined organic layers were dried over Na_2SO_4, filtered and then dried in vacuo at 100 °C, affording **1** as a yellow-brown solid (875 mg, 84%). Analytical data are identical to those published.[S2]

^{1}H NMR (400 MHz, $CDCl_3$) δ = 6.98 – 6.87 (m, 6H), 6.75 (ddd, *J* = 9.4, 6.3, 1.8 Hz, 2H), 6.20 (ddd, *J* = 9.4, 1.4, 0.7 Hz, 2H), 5.66 (ddd, *J* = 6.9, 6.3, 1.4 Hz, 2H), 3.28 (s, 6H)

Complex 2

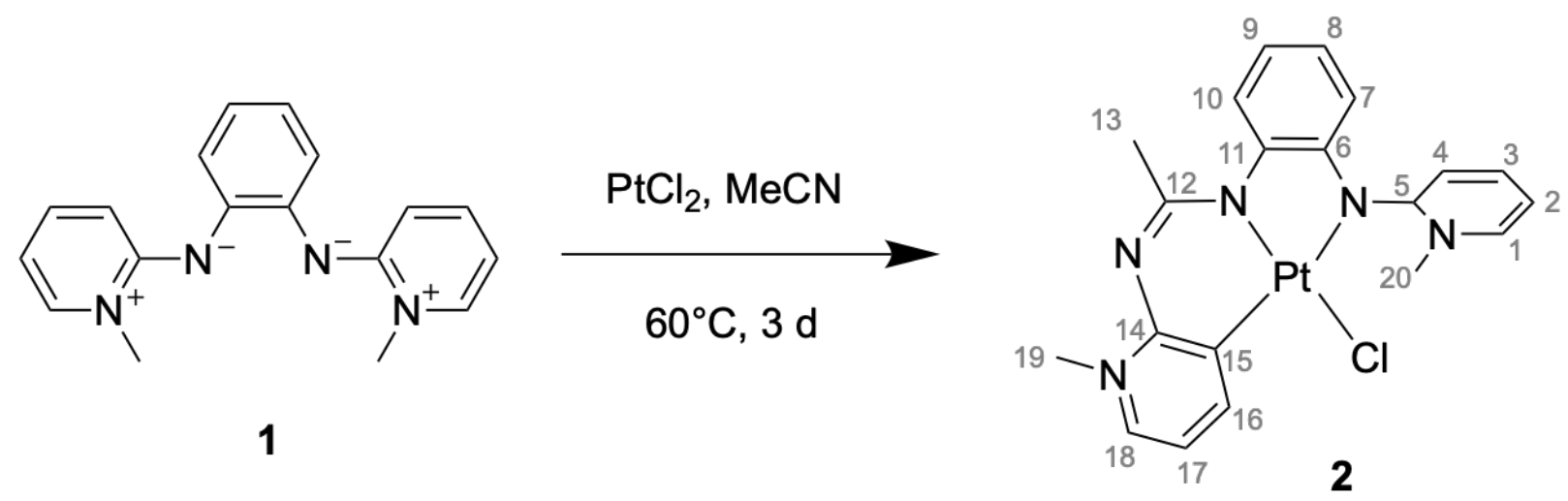

In a vial, $PtCl_2$ (189 mg, 0.71 mmol) was dissolved in dry MeCN (5 mL) at 80 °C for 20 min to give a clear yellow solution. The solution was cooled to 60 °C and a solution of bisPYA **1** (206 mg, 0.71 mmol) dissolved in dry MeCN (10 mL) and preheated to 60 °C was added. The mixture was stirred at 60 °C for 3 days (or 5 h at 80 °C), resulting in a dark red solution. All volatiles were removed in vacuo. The residue was dissolved in CH_2Cl_2 and dried again in vacuo to remove all the rest of MeCN, yielding a dark red solid. The solid was purified over a short Al_2O_3 plug using first CH_2Cl_2 and then MeCN as eluent. Complex **2** eluted with CH_2Cl_2 and was dried in vacuo, yielding a red solid (174 mg, 44 %).

^{1}H NMR (400 MHz, CD_2Cl_2) δ = 8.51 (d, J = 1.7 Hz, $^2J_{PtH}$ = 7.4, 1H, H16), 7.85 (d, J = 6.5 Hz, 1H, H1), 7.71–7.60 (m, 2H, H3 + H4), 7.48 (dd, J = 6.2, 1.8 Hz, 1H, H18), 7.08 (dd, J = 8.1, 1.5 Hz, 1H, H10), 6.95 (dd, J = 8.0, 1.4 Hz, 1H, H7), 6.83–6.73 (m, 2H, H8 + H2), 6.68 (ddd, J = 8.5, 7.2, 1.4 Hz, 1H, H9), 6.54 (dd, J = 7.5, 6.1 Hz, 1H, H17), 4.13 (s, 3H, H20), 3.95 (s, 3H, H19), 2.44 (s, 3H, H13).

^{13}C NMR (100 MHz, CD_2Cl_2) δ = 165.23 (q, C5), 157.44 (q, C12), 150.58 (C16), 148.08(q, C14), 147.14 (q, C6), 145.12(q, C11), 141.28 (C1), 139.50 (C3), 134.44 (C18), 124.49 (C4), 123.23 (C8), 123.05 (C10), 120.66 (q, C15), 118.25(C9), 114.95 (C2), 114.43 (C7), 112.98 (C17), 43.85 (C20), 43.59 (C19), 25.99 (C13)

HR-ESI-MS (m/z): calculated for $C_{20}H_{21}ClN_5Pt$ $[M+H]^+$ = 561.1128; found: 561.1126

Elemental analysis calculated for $C_{20}H_{20}ClN_5Pt$ x 0.4 CH_2Cl_2: C 41.19, H 3.52, N 11.77; found: C 41.50, H 3.58, N 11.46%.

Complex 3

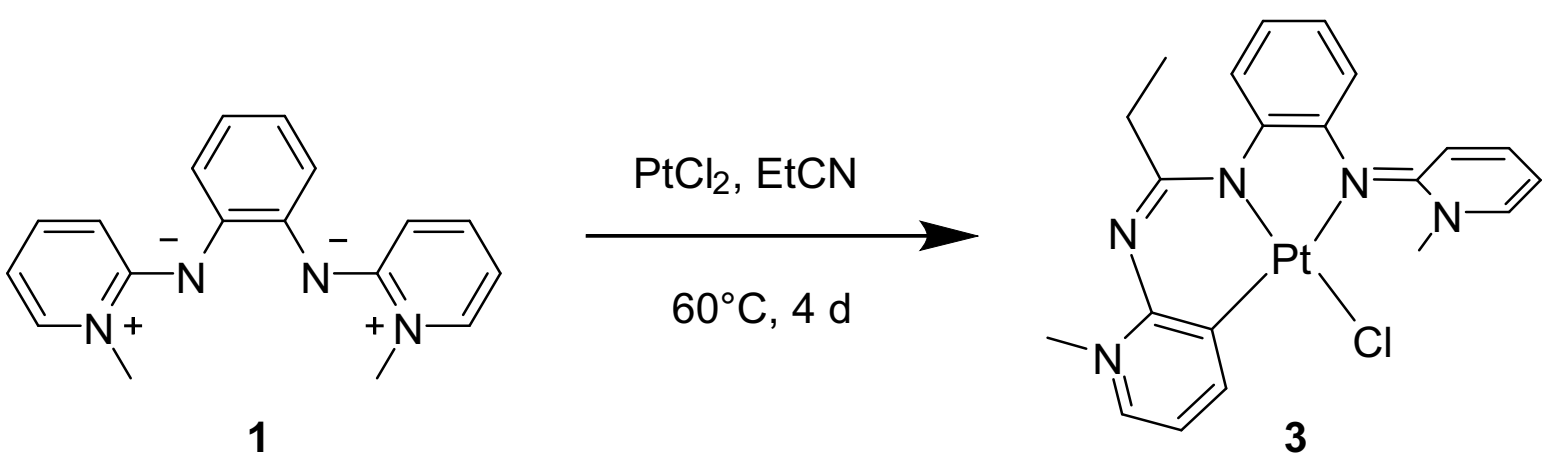

In a vial $PtCl_2$ (110 mg, 0.41 mmol) was dissolved in dry EtCN (5 mL) at 80 °C for 20 min to give a clear yellow solution. The solution was cooled to 70 °C and added to a two-necked round bottom flask with bisPYA **1** (120 mg, 0.41 mmol). The mixture was stirred at 60 °C for 4 days, resulting in a dark red solution. All volatiles were removed in vacuo. The residue was dissolved in CH_2Cl_2 and dried again in vacuo to get rid of the remaining EtCN. A dark red-brown complex mixture was obtained as a solid.

The solid was purified over an Al_2O_3 plug using CH_2Cl_2 as an eluent. The cyclometalated product was dried in vacuo, yielding complex **3** as a red solid (98 mg, 41 %).

^{1}H NMR (400 MHz, CD_2Cl_2) δ = 8.49 (d, 1.8 Hz, $^2J_{PtH}$ = 7.5, 1H), 7.84 (dt, *J* = 6.7, 1.3 Hz, 1H), 7.70–7.62 (m, 2H), 7.48 (dd, *J* = 6.2, 1.7 Hz, 1H), 7.02 (dd, *J* = 8.0, 1.5 Hz, 1H), 6.96 (dd, *J* = 8.0, 1.4 Hz, 1H), 6.81–6.74 (m, 2H), 6.67 (ddd, *J* = 8.0, 7.2, 1.4 Hz, 1H), 6.54 (dd, *J* = 7.4, 6.2 Hz, 1H), 4.14 (s, 3H), 3.98 (s, 3H), 2.77 (q, *J* = 7.6 Hz, 2H), 1.44 (t, *J* = 7.3 Hz, 3H)

^{13}C NMR (100 MHz, CD_2Cl_2) δ = 165.19, 162.08, 150.48, 148.22, 146.93, 144.94, 141.23, 139.41, 134.38, 124.40, 123.13, 122.37, 120.86, 118.31, 114.83, 114.47, 112.94, 43.87, 43.57, 30.24, 13.64

HR-ESI-MS (m/z): calculated for $C_{21}H_{23}ClN_5Pt$ $[M+H]^+$ = 575.1284; found: 575.1286

Elemental analysis calculated for $C_{21}H_{22}ClN_5Pt$ x 0.2 CH_2Cl_2: C 42.94%, H 3.98%, N 11.81%; found: C 43.23%, H 3.85%, N 11.44%

Complex 4

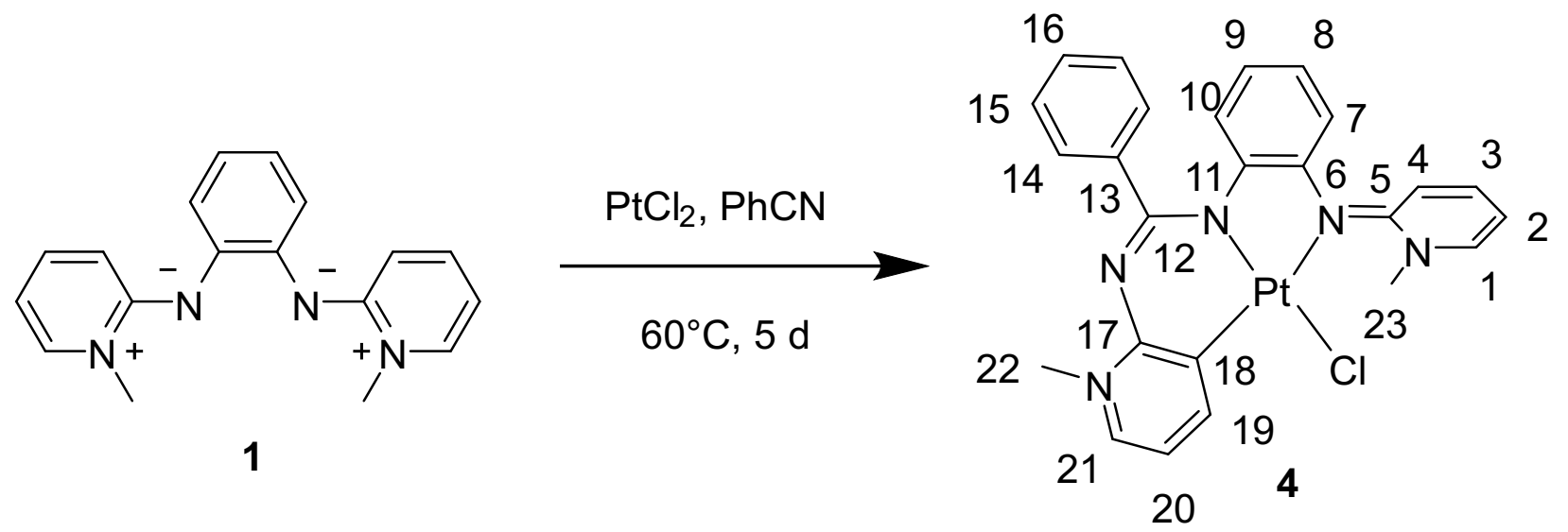

In a vial, $PtCl_2$ (137 mg, 0.52 mmol) was dissolved in dry PhCN (5 mL) at 90 °C for 20 min to give a clear yellow solution. The solution was cooled to 60 °C and a solution of bisPYA **1** (150 mg, 0.52 mmol) in dry PhCN (5 mL) was added. The mixture was stirred and heated to 60 °C for 5 days, resulting in a dark red solution. The solution was cooled to RT and a solid was crushed out by slowly adding Et_2O. The mixture was filtered and the solid was washed with Et_2O, resulting in a dark red solid and red filtrate. The dark red solid was collected, dried in vacuo and purified over an Al_2O_3 plug using CH_2Cl_2 as eluent. The solution was dried in vacuo yielding a red solid (isomer mixture). The red Et_2O filtrate was concentrated to 50 mL in vacuo and then purified by an Al_2O_3 plug using CH_2Cl_2 as eluent. The resulting waxy red solid was dissolved in DCM (5 mL) and layered with 60 mL of pentane. The resulting red solid was washed with pentane and then dried in vacuo to yield the pure cyclometalated complex **4** as a red solid (61 mg, 19%).

^{1}H NMR (400 MHz, CD_2Cl_2) δ = 8.68 (d, *J* = 1.7 Hz, $^2J_{PtH}$ = 7.5, 1H, H19), 7.89 (dt, *J* = 6.7, 1.3 Hz, 1H, H1), 7.74–7.65 (m, 2H, H3 + H4), 7.62 (d, *J* = 7.0 Hz, 2H, H14), 7.56 (dd, *J* = 6.2, 1.7 Hz, 1H, H21), 7.40 - 7.33 (m, 1H, H16), 7.33–7.27 (m, 2H, H15), 6.94 (dd, *J* = 8.1, 1.4 Hz, 1H, H10), 6.80 (td, *J* = 6.3, 2.3 Hz, 1H, H2), 6.69 – 6.56 (m, 2H, H20 + H9), 6.39 (dd, *J* = 8.1, 1.4 Hz, 1H, H7), 6.24 (ddd, *J* = 8.3, 7.1, 1.4 Hz, 1H, C8-H), 4.23 (s, 3H, H23), 4.06 (s, 3H, H22)

^{13}C NMR (100 MHz, CD_2Cl_2) δ = δ 165.21 (C5), 156.36 (C12), 151.55 (C19), 148.13 (C17), 146.82 (C11), 145.33 (C6), 141.28 (C1 + C13), 139.43 (C3), 134.93 (C21), 129.91 (C14), 129.09 (C16), 128.36 (C15), 124.64 (C7), 124.61 (C4), 122.49 (C9), 122.02 (C18), 117.82 (C8), 114.93 (C20), 114.15 (C10), 113.45 (C2), 44.14 (C23), 43.64 (C22)

HR-ESI-MS (m/z): calculated for $C_{25}H_{23}N_5ClPt$ $[M+H]^+$ = 623.1284; found: 623.1285

Elemental analysis calculated for $C_{25}H_{22}N_5ClPt$ x 0.5 CH_2Cl_2: C 46.02%, H 3.48%, N 10.52%; found: C 46.10%, H 3.47%, N 10.46%

2. ^{15}N labeling experiment

Synthesis of complex 2-^{15}N

bisPYA **1** (30 mg, 0.10 mmol, 1.0 equiv.) and $PtCl_2$ (28mg, 0.10 mmol, 1.0 equiv.) were added to a 10 mL Schlenk flask. of $MeC^{15}N$ (0.5 mL) was added. The mixture was stirred at 60 °C for 3 days, resulting in a dark solution. All volatiles were evaporated in vacuo. The residue was dissolved in CH_2Cl_2 and dried again in vacuo, yielding a dark green solid. The solid was purified over a short Al_2O_3 plug using first CH_2Cl_2 and then MeCN as an eluent. The CH_2Cl_2 fraction was collected and dried in vacuo, yielding complex **2-^{15}N** as an orange solid (10 mg, 17%).

^{1}H-^{15}N HMBC NMR coupling (400 MHz, 40 MHz, CD_2Cl_2)**:** 7.48 (H18 weak), 3.95 (H19, strong), 2.44 (H13, very strong).

HR-ESI-MS (m/z): calculated for $C_{20}H_{21}ClN_4{}^{15}N$ Pt $[M+H]^+$ = 562.1098; found: 562.1109

The resulting complex was obtained as a mixture of 2 species containing **2-^{15}N** and a non MeCN containing side product, which was also observed during the one pot synthesis of **2**. The NMR shifts of **2-^{15}N** were mostly identical to **2** and further checked by 2D-NMR of the product mixture. The second reaction species was also isolated on small scale during the synthesis of **2** with 2.5 % v/v H_2O added to the acetonitrile. The H-NMR spectra of the product mixture of **2-^{15}N**, **2** and the isolated second species from the H_2O reaction are shown in Fig. 1.

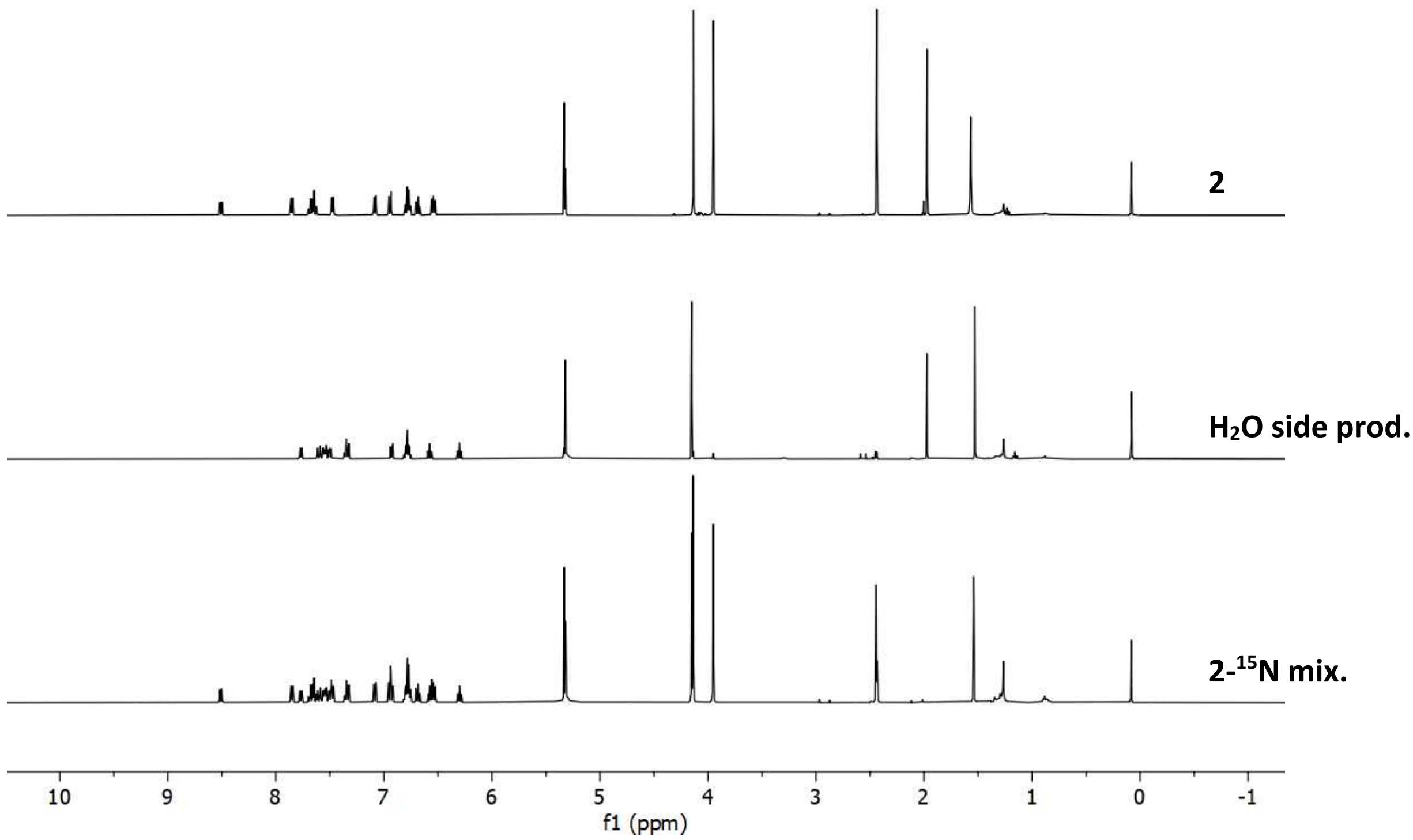

Figure S1: ^{1}H NMR spectra of the **2-^{15}N** product mixture, **2** and the isolated second species from the one pot reaction of **2** with H_2O in CD_2Cl_2

3. Kinetic experiments

3.1 Temperature-dependent reactions

A stock solution of $Pt(CD_3CN)_2Cl_2$ (43 mM) was prepared by adding $PtCl_2$ (46 mg) and CD_3CN (4.0 mL) to a microwave vial. The mixture was stirred at 80 °C for 16 h. Three J-Young NMR tubes loaded with bisPYA **1** (7.4 to 12.5 mg) were prepared. An equivalent molar amount of $Pt(CD_3CN)_2Cl_2$ was added via the stock solution. The NMR tubes were heated to 50, 60 and 80 °C, respectively, and ^{1}H NMR spectra were measured at 25 °C after 5 min, 30 min, 1 h, 2 h, 3 h, 4 h, 5 h, 20 h, 24 h, 28 h, 44 h and 48 h of reaction time. Analysis was restricted to the aliphatic area (3.0–4.5 ppm) featuring the characteristic N–CH_3 resonances. The aromatic section was too complex, as the presence of signals of the protonated ligand, the dissymmetric product, and two dissymmetric intermediates (one as a mixture of two isomers) yielded >60 independent aromatic protons that resonate all in in the 6–9 ppm range at various intensities.

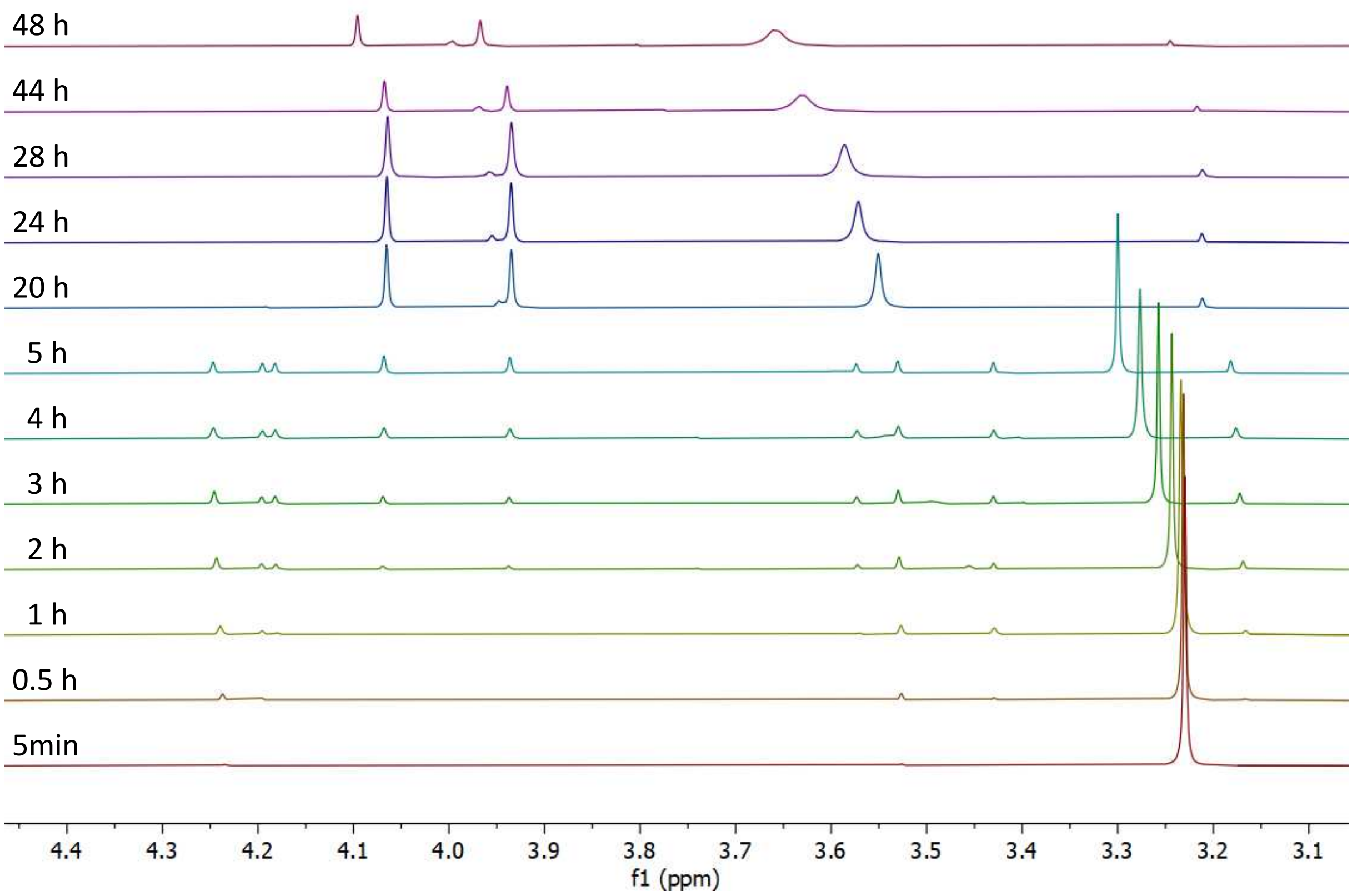

Figure S2: Stacked ^{1}H NMR spectra of the PYA methyl region over 48 h at 50°C.

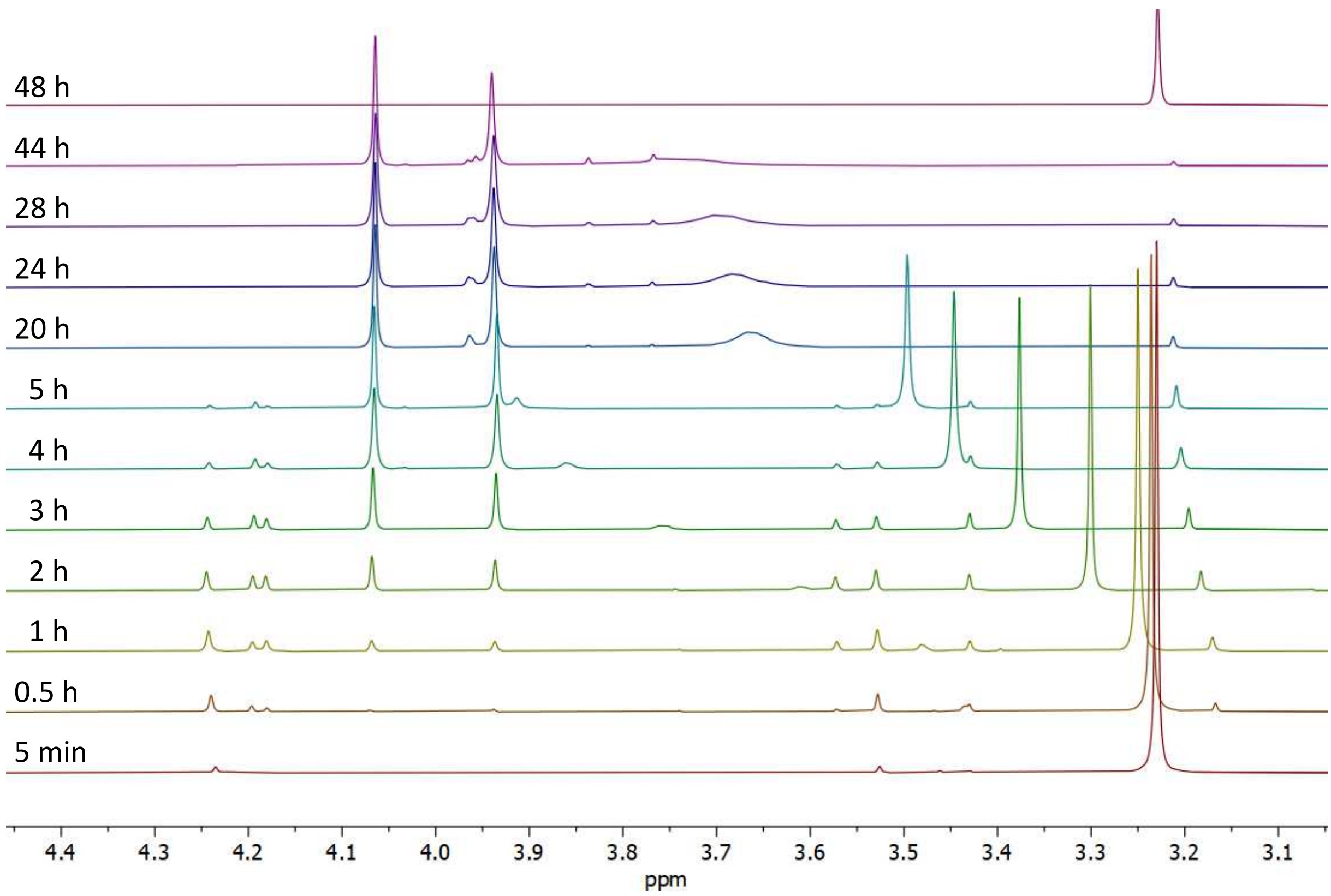

Figure S3: Stacked ^{1}H NMR spectra of the PYA methyl region over 48 h at 60 °C

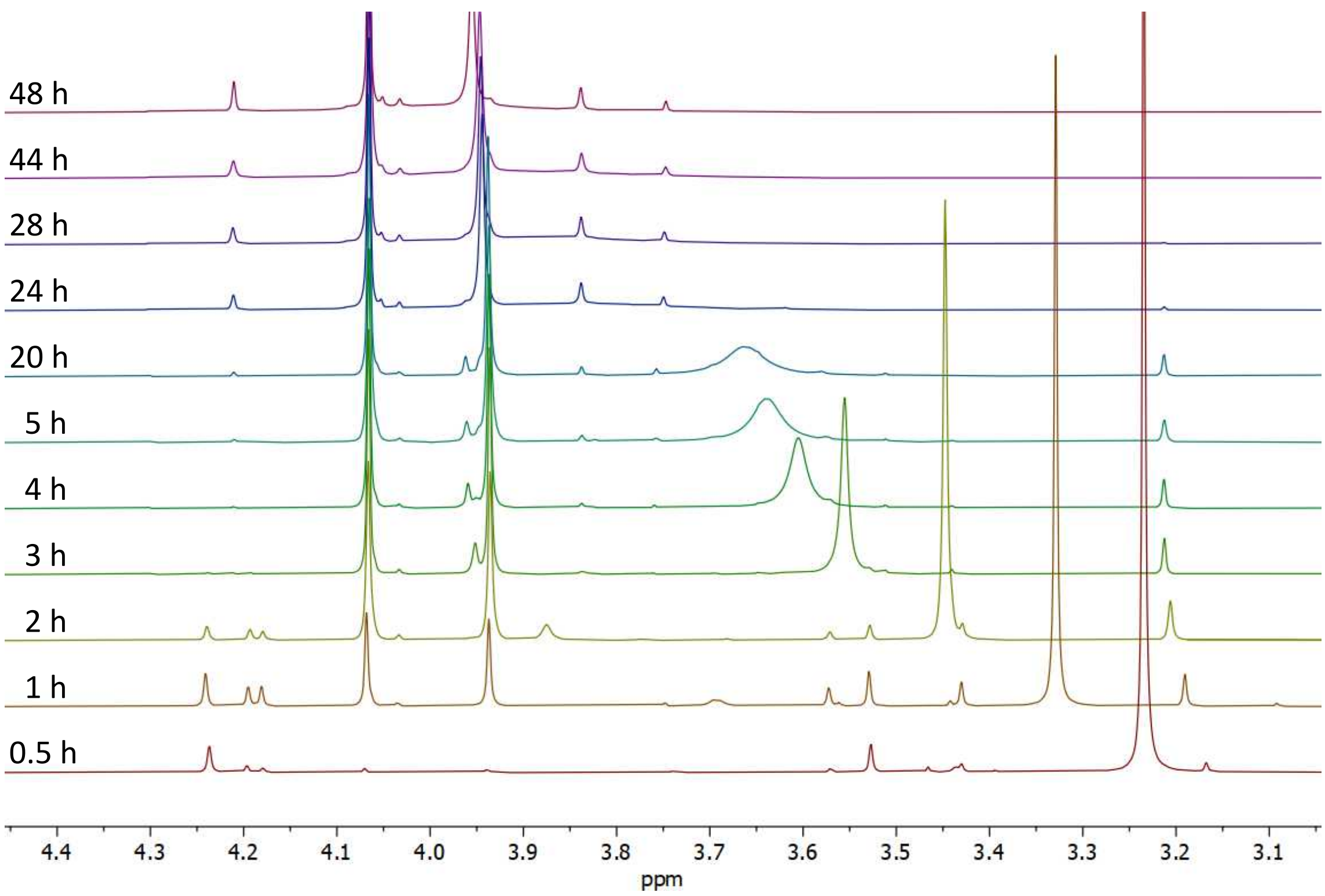

Figure S4: Stacked ^{1}H NMR spectra of the PYA methyl region over 48 h at 80 °C

3.2 Continuous reaction monitoring at 60 °C and 75 °C

Under argon atmosphere, dry CD_3CN (502 mg) was added to a J-Young NMR tube loaded with $PtCl_2$ (8.60 mg, 32 μmol) and hexamethylbenzene (1.36 mg, 8.4 μmol) as internal standard. The mixture was heated to 75 °C for 14 h, then cooled to RT and thermostated at 60 °C in a Bruker 400 MHz spectrometer. A first ^1H NMR was recorded. Then bisPYA **1** (9.39 mg, 32 μmol) was rapidly added and the tube placed back in the spectrometer at 60 °C. After 3 min, consecutive ^1H NMR spectra were measured every 10 min for the first 3 h and then in 20 min intervals up until 7 h.

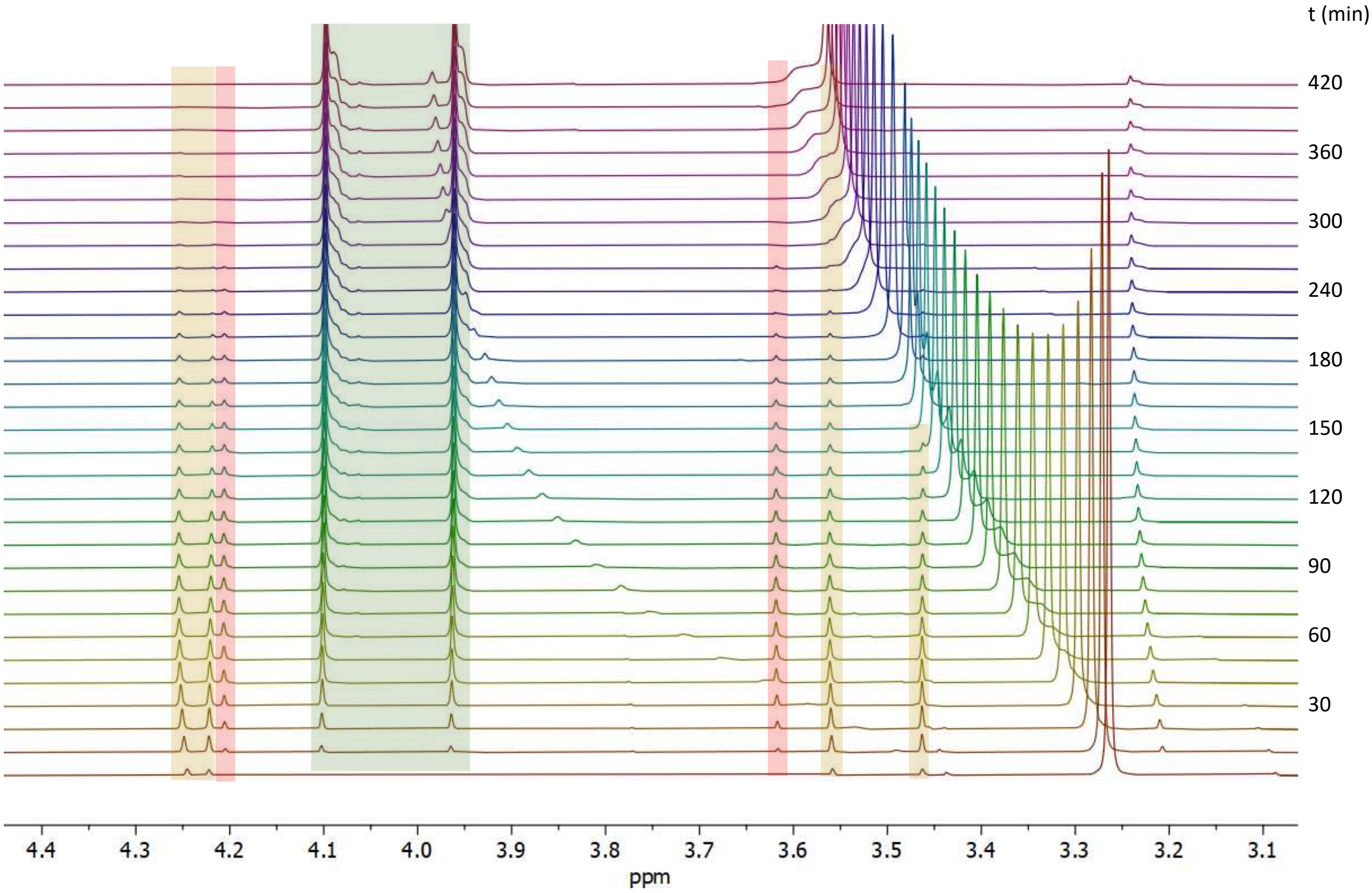

Figure S5: Stacked ^1H NMR spectra of the PYA methyl region from 0 to 420 min at 60°C. Intermediates **A** (yellow), **B** (red) and the cyclometalated product **2** (green) are highlighted

The experiment was repeated at 75 °C starting from $PtCl_2$ (7.96 mg, 30 μmol), dry CD_3CN (552 mg), hexamethylbenzene (4.05 mg, 25.0 μmol) and bisPYA **1** (8.07 mg, 28 μmol) at 75 °C. Consecutive ^1H NMR spectra were recorded in 3 min intervals for the first 60 min, then in 5 min intervals up until 2 h.

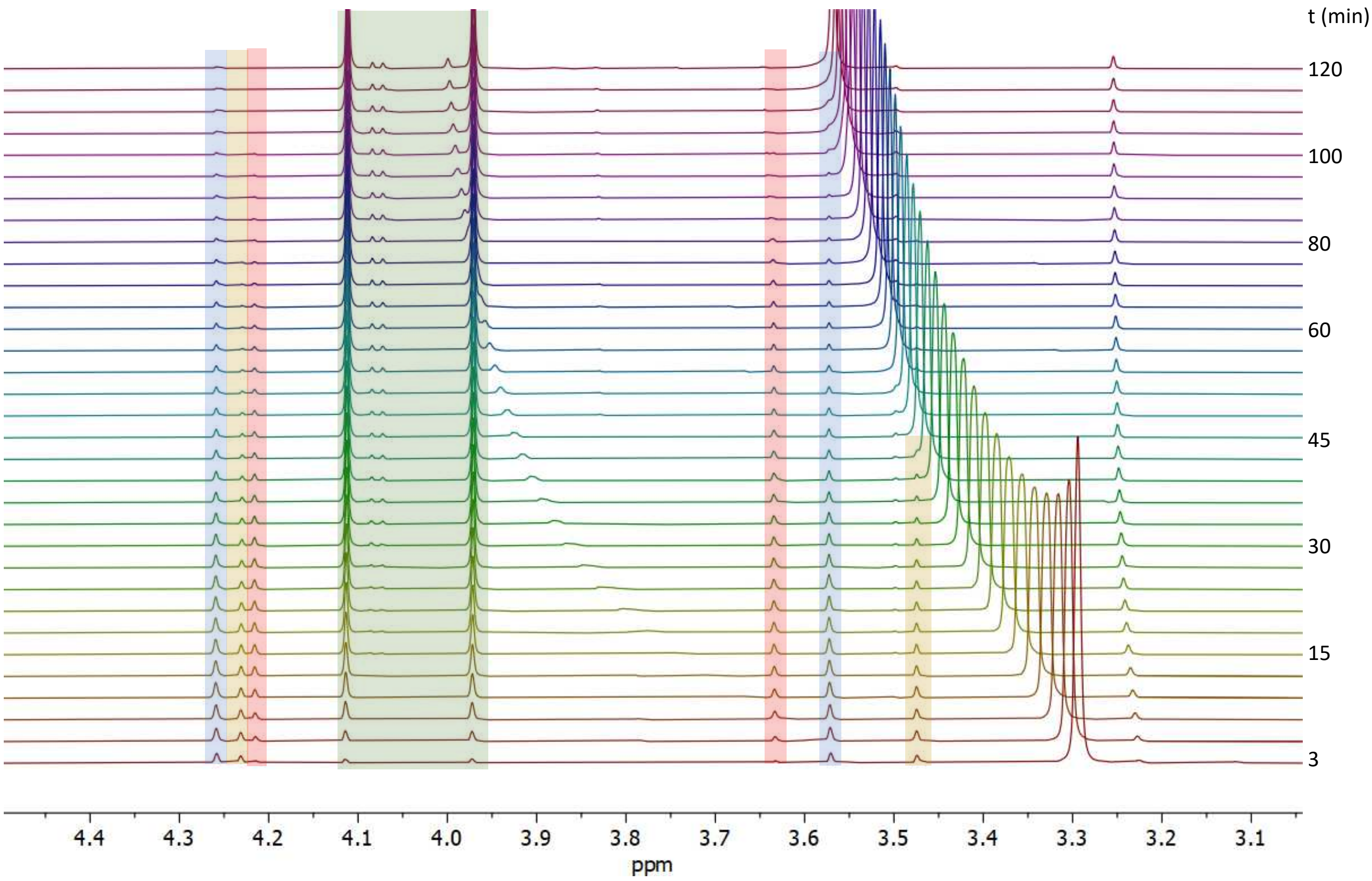

Figure S6: Stacked ^{1}H NMR spectra of the PYA methyl region from 0 to 120 min at 75 °C. Intermediate **A** isomers (yellow and blue), intermediate **B** (red) and the cyclometalated product **2** (green) are highlighted

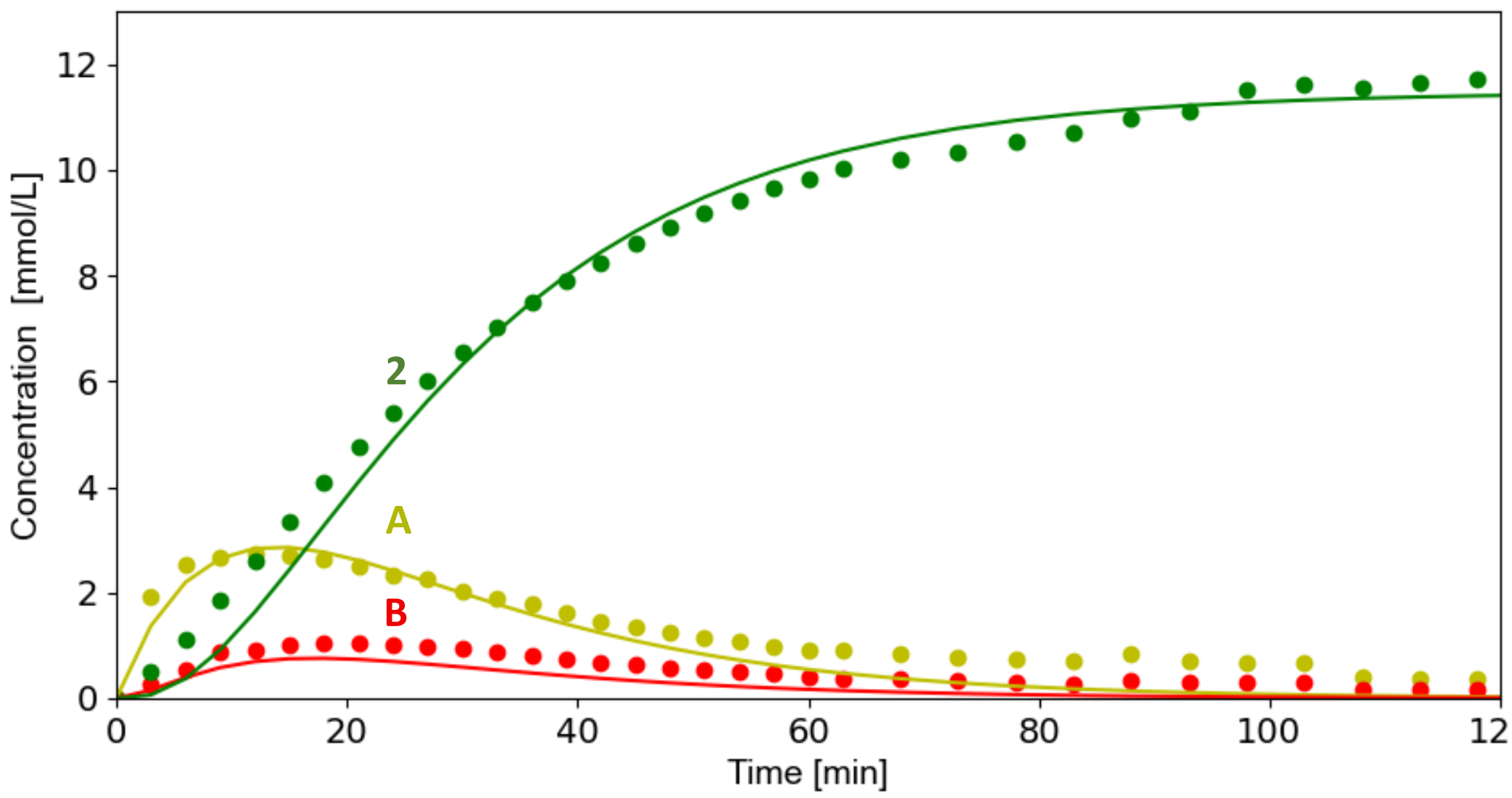

Figure S7: Time-dependent concentration of intermediates **A** (yellow as sum of both isomers), **B** (red) and the cyclometalated product **2** (green) at 75 °C; full circles are measured concentrations, solid lines are fitted concentrations

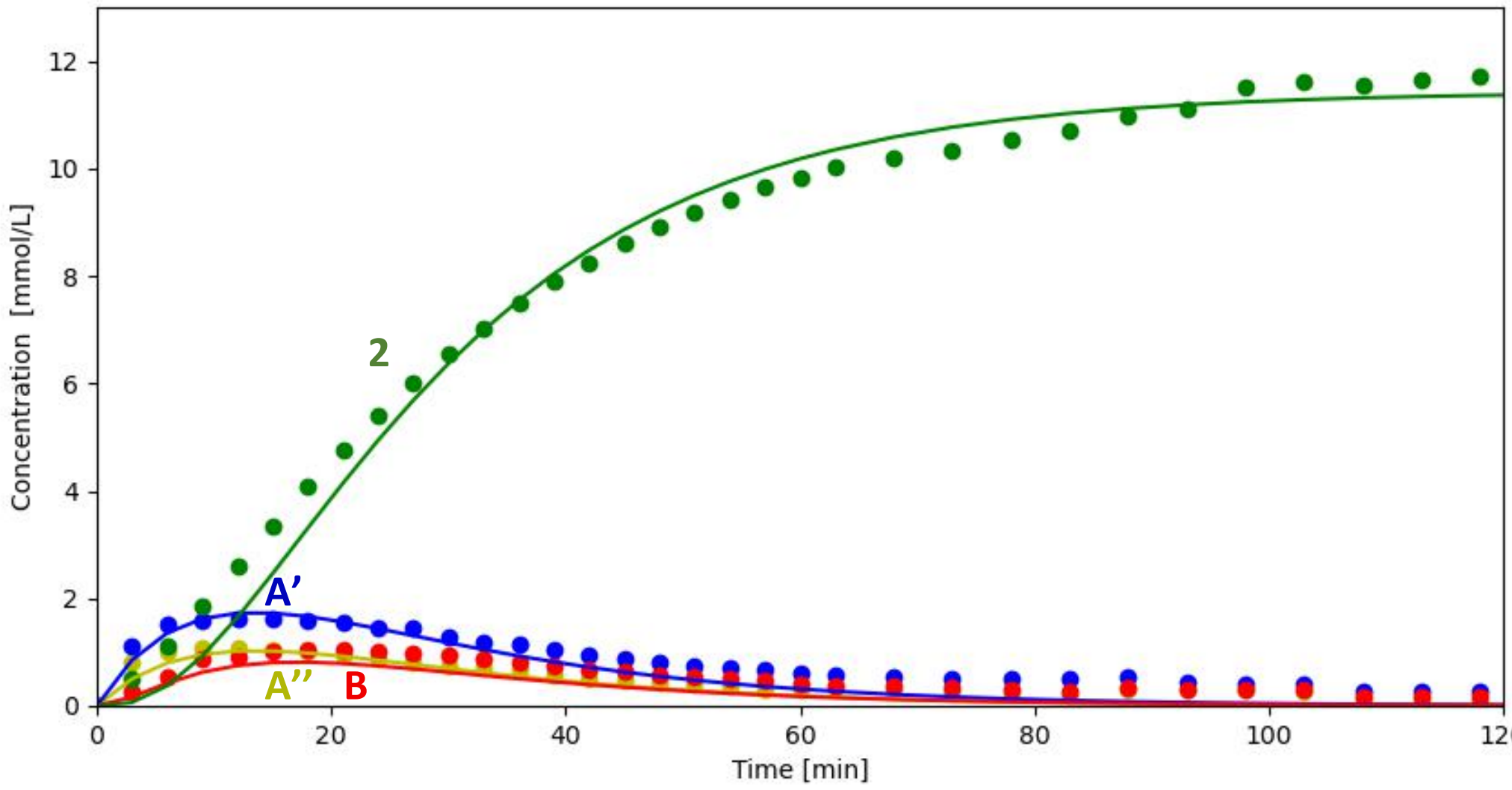

Figure S8: Time-dependent concentration of intermediates **A** isomers (yellow and blue), intermediate **B** (red) and the cyclometalated product **2** (green) at 75 °C

Tables of raw integrals and concentrations from time-dependent reaction monitoring

Due to overlap of the shifting ligand signal and a shifting side reaction signal (3.5 to 4 ppm), the concentrations of several species were overlap corrected. Integrals of the species were replaced with another methyl signal of the same species in case of overlap. Overlapping species were also subtracted from the free ligand signal, if they were larger than 2% of its integral. Concentrations were only overlap corrected when there was a visible overlap of the two signals in the NMR spectrum, these entries are marked in the Tables by blue background.

Table S1: Raw integrals for hexamethylbenzene standard, ligand (as a mixture of **1** and **1.HX**), **2** (green), intermediate A (yellow), intermediate B (red) of the experiment at 60 °C

time [min]	Standard	**1** and **1.HX**	**2** (4.09 ppm)	**2** (3.96 ppm)	4.24 ppm	4.22 ppm	4.20 ppm	3.61 ppm	3.56 ppm	3.46 ppm
0	10.00	9.12	0.01	0.01	0.10	0.09	0.01	0.01	0.10	0.10
10	10.00	8.14	0.09	0.09	0.24	0.23	0.06	0.05	0.22	0.22
20	10.00	7.44	0.21	0.21	0.29	0.29	0.11	0.11	0.28	0.28
30	10.00	6.88	0.35	0.35	0.31	0.31	0.16	0.16	0.31	0.29
40	10.00	6.48	0.50	0.50	0.31	0.31	0.20	0.24	0.30	0.29
50	10.00	6.20	0.65	0.65	0.31	0.30	0.22	0.21	0.28	0.30
60	10.00	5.96	0.79	0.79	0.29	0.28	0.23	0.22	0.26	0.25
70	10.00	5.77	0.93	0.93	0.28	0.26	0.24	0.21	0.25	0.23
80	10.00	5.64	1.06	1.05	0.26	0.24	0.23	0.21	0.23	0.21
90	10.00	5.56	1.18	1.16	0.24	0.22	0.22	0.20	0.23	0.19

100	10.00	5.55	1.29	1.27	0.22	0.19	0.21	0.20	0.20	0.18
110	10.00	5.63	1.40	1.38	0.20	0.17	0.20	0.18	0.18	0.16
120	10.00	5.79	1.49	1.46	0.18	0.16	0.18	0.16	0.16	0.15
130	10.00	5.77	1.58	1.55	0.16	0.14	0.17	0.17	0.15	0.15
140	10.00	5.80	1.68	1.63	0.16	0.13	0.16	0.17	0.14	0.19
150	10.00	5.70	1.75	1.70	0.13	0.11	0.14	0.13	0.11	2.78
160	10.00	5.70	1.82	1.76	0.13	0.10	0.13	0.13	0.11	3.13
170	10.00	5.68	1.88	1.82	0.12	0.09	0.12	0.12	0.10	0.54
180	10.00	5.61	1.94	1.88	0.11	0.08	0.11	0.11	0.09	0.10
200	10.00	5.50	2.05	2.00	0.09	0.06	0.09	0.09	0.07	0.05
220	10.00	5.50	2.14	2.22	0.07	0.05	0.07	0.08	0.07	0.05
240	10.00	5.43	2.22	2.38	0.06	0.04	0.06	0.07	0.07	0.03
260	10.00	5.48	2.29	2.47	0.05	0.03	0.05	0.06	0.11	0.03
280	10.00	5.44	2.33	2.52	0.05	0.03	0.04	0.05	0.22	0.02
300	10.00	5.43	2.40	2.55	0.05	0.03	0.04	0.05	0.62	0.02
320	10.00	5.39	2.44	2.52	0.04	0.02	0.03	0.03	0.90	0.02
340	10.00	5.37	2.48	2.48	0.04	0.02	0.03	0.04	0.98	0.02
360	10.00	5.33	2.53	2.52	0.03	0.02	0.02	0.05	1.30	0.01
380	10.00	5.34	2.58	2.57	0.04	0.02	0.03	0.08	3.12	0.02
400	10.00	5.26	2.60	2.58	0.03	0.01	0.02	0.11	3.14	0.01
420	10.00	5.20	2.63	2.62	0.03	0.01	0.01	0.16	2.59	0.01

Table S2: Concentrations [mmol/L] derived from hexamethylbenzene standard for ligand (as a mixture of **1** and **1.HX**), **2** (green), intermediate A (yellow, two putative isomers), intermediate B (red) of the experiment at 60 °C

time [min]	Standard	**1** and **1.HX**	**2** (4.09 ppm)	**2** (3.96 ppm)	4.24 ppm	4.22 ppm	4.20 ppm	3.61 ppm	3.56 ppm	3.46 ppm
0	14.09	38.53	0.07	0.06	0.87	0.73	0.10	0.09	0.83	0.83
10	14.09	34.39	0.77	0.77	1.99	1.94	0.51	0.46	1.87	1.84
20	14.09	31.42	1.80	1.80	2.48	2.44	0.96	0.92	2.35	2.37
30	14.09	29.07	2.96	2.99	2.65	2.59	1.35	1.36	2.60	2.45
40	14.09	27.40	4.25	4.26	2.66	2.59	1.65	1.99	2.53	2.44
50	14.09	26.21	5.50	5.49	2.59	2.50	1.85	1.79	2.38	2.50
60	14.09	25.20	6.71	6.66	2.45	2.34	1.93	1.83	2.24	2.13
70	14.09	24.40	7.90	7.82	2.34	2.19	1.99	1.80	2.08	1.95
80	14.09	23.83	8.92	8.83	2.18	2.01	1.95	1.76	1.92	1.77
90	14.09	23.48	9.95	9.83	2.01	1.83	1.88	1.72	1.96	1.62
100	14.09	23.45	10.93	10.73	1.85	1.63	1.80	1.65	1.68	1.49
110	14.09	23.77	11.80	11.67	1.65	1.45	1.66	1.52	1.48	1.34
120	14.09	24.48	12.62	12.36	1.55	1.31	1.56	1.39	1.38	1.25
130	14.09	24.39	13.37	13.09	1.37	1.15	1.41	1.46	1.23	1.23
140	14.09	24.53	14.20	13.82	1.33	1.07	1.37	1.46	1.19	1.65
150	14.09	24.09	14.76	14.36	1.13	0.89	1.19	1.12	0.95	23.51
160	14.09	24.09	15.39	14.88	1.09	0.83	1.12	1.12	0.93	26.41
170	14.09	24.01	15.92	15.42	0.98	0.74	1.01	1.02	0.88	4.60
180	14.09	23.72	16.43	15.91	0.89	0.65	0.91	0.91	0.79	0.84

200	14.09	23.24	17.31	16.88	0.73	0.51	0.75	0.75	0.62	0.45
220	14.09	23.26	18.12	18.73	0.63	0.42	0.61	0.68	0.60	0.40
240	14.09	22.95	18.77	20.15	0.53	0.33	0.50	0.59	0.59	0.27
260	14.09	23.14	19.32	20.87	0.46	0.26	0.41	0.54	0.95	0.26
280	14.09	22.99	19.71	21.32	0.39	0.22	0.34	0.43	1.90	0.21
300	14.09	22.95	20.26	21.59	0.39	0.21	0.31	0.38	5.22	0.19
320	14.09	22.79	20.66	21.27	0.37	0.19	0.28	0.26	7.60	0.15
340	14.09	22.71	20.99	20.97	0.31	0.14	0.22	0.34	8.30	0.13
360	14.09	22.51	21.38	21.28	0.27	0.13	0.19	0.43	11.02	0.08
380	14.09	22.55	21.83	21.69	0.33	0.17	0.23	0.65	26.38	0.16
400	14.09	22.21	21.99	21.84	0.23	0.09	0.14	0.89	26.53	0.12
420	14.09	21.97	22.24	22.14	0.23	0.09	0.13	1.33	21.90	0.12

Table S3: Overlap-corrected concentrations [mmol/L] for hexamethylbenzene standard, ligand (as a mixture of **1** and **1.HX**), **2** (green), intermediate A (yellow), intermediate B (red) of the experiment at 60 °C. Overlap corrected concentrations are marked in blue.

time [min]	Standard	**1** and **1.HX**	**2** (4.09 ppm)	**2** (3.96 ppm)	4.24 ppm	4.22 ppm	4.20 ppm	3.61 ppm	3.56 ppm	3.46 ppm
0	14.09	38.53	0.07	0.06	0.87	0.73	0.10	0.09	0.83	0.83
10	14.09	34.39	0.77	0.77	1.99	1.94	0.51	0.46	1.87	1.84
20	14.09	31.42	1.80	1.80	2.48	2.44	0.96	0.92	2.35	2.37
30	14.09	29.07	2.96	2.99	2.65	2.59	1.35	1.36	2.60	2.45
40	14.09	27.40	4.25	4.26	2.66	2.59	1.65	1.99	2.53	2.44
50	14.09	26.21	5.50	5.49	2.59	2.50	1.85	1.79	2.38	2.50
60	14.09	25.20	6.71	6.66	2.45	2.34	1.93	1.83	2.24	2.13
70	14.09	24.40	7.90	7.82	2.34	2.19	1.99	1.80	2.08	1.95
80	14.09	23.83	8.92	8.83	2.18	2.01	1.95	1.76	1.92	1.77
90	14.09	23.48	9.95	9.83	2.01	1.83	1.88	1.72	1.96	1.62
100	14.09	23.45	10.93	10.73	1.85	1.63	1.80	1.65	1.68	1.49
110	14.09	23.77	11.80	11.67	1.65	1.45	1.66	1.52	1.48	1.34
120	14.09	23.86	12.62	12.36	1.55	1.31	1.56	1.39	1.38	1.25
130	14.09	23.77	13.37	13.09	1.37	1.15	1.41	1.46	1.23	1.23
140	14.09	23.93	14.20	13.82	1.33	1.07	1.37	1.46	1.19	1.19
150	14.09	23.61	14.76	14.36	1.13	0.89	1.19	1.12	0.95	0.95
160	14.09	23.62	15.39	14.88	1.09	0.83	1.12	1.12	0.93	0.93
170	14.09	23.57	15.92	15.42	0.98	0.74	1.01	1.02	0.88	0.88
180	14.09	23.72	16.43	15.91	0.89	0.65	0.91	0.91	0.79	0.84
200	14.09	23.24	17.31	16.88	0.73	0.51	0.75	0.75	0.62	0.45
220	14.09	23.26	18.12	18.12	0.63	0.42	0.61	0.68	0.60	0.40
240	14.09	22.95	18.77	18.77	0.53	0.33	0.50	0.59	0.59	0.27
260	14.09	23.14	19.32	19.32	0.46	0.26	0.41	0.54	0.26	0.26
280	14.09	22.99	19.71	19.71	0.39	0.22	0.34	0.43	0.21	0.21
300	14.09	22.95	20.26	20.26	0.39	0.21	0.31	0.38	0.19	0.19
320	14.09	22.79	20.66	20.66	0.37	0.19	0.28	0.26	0.15	0.15
340	14.09	22.71	20.99	20.97	0.31	0.14	0.22	0.22	0.13	0.13
360	14.09	22.51	21.38	21.28	0.27	0.13	0.19	0.19	0.08	0.08

380	14.09	22.55	21.83	21.69	0.33	0.17	0.23	0.23	0.16	0.16
400	14.09	22.21	21.99	21.84	0.23	0.09	0.14	0.14	0.12	0.12
420	14.09	21.97	22.24	22.14	0.23	0.09	0.13	0.13	0.12	0.12

Table S4: Raw integrals for hexamethylbenzene standard, ligand (as a mixture of **1** and **1.HX**), **2** (green), intermediate A' (blue), intermediate A'' (yellow), intermediate B (red) of the experiment at 75 °C

time [min]	Standard	**1** and **1.HX**	**2** (4.11 ppm)	**2** (3.97 ppm)	4.26 ppm	4.23 ppm	4.21 ppm	3.63 ppm	3.57 ppm	3.47 ppm
0	10.00	2.36	0.02	0.02	0.05	0.04	0.01	0.01	0.05	0.04
3	10.00	2.21	0.05	0.05	0.06	0.04	0.02	0.02	0.07	0.05
6	10.00	2.08	0.08	0.08	0.07	0.05	0.03	0.04	0.07	0.05
9	10.00	1.98	0.11	0.11	0.07	0.05	0.04	0.04	0.07	0.05
12	10.00	1.89	0.15	0.15	0.07	0.05	0.04	0.04	0.07	0.05
15	10.00	1.81	0.18	0.18	0.07	0.05	0.05	0.05	0.07	0.04
18	10.00	1.75	0.21	0.21	0.07	0.04	0.05	0.05	0.07	0.04
21	10.00	1.69	0.24	0.24	0.06	0.04	0.04	0.04	0.06	0.04
24	10.00	1.65	0.26	0.26	0.06	0.04	0.04	0.04	0.07	0.04
27	10.00	1.62	0.29	0.29	0.05	0.03	0.04	0.04	0.06	0.03
30	10.00	1.58	0.31	0.31	0.05	0.03	0.04	0.04	0.05	0.03
33	10.00	1.55	0.33	0.33	0.05	0.03	0.03	0.04	0.05	0.03
36	10.00	1.52	0.35	0.34	0.04	0.03	0.03	0.04	0.05	0.03
39	10.00	1.51	0.36	0.36	0.04	0.02	0.03	0.04	0.04	0.05
42	10.00	1.49	0.38	0.37	0.04	0.02	0.03	0.04	0.04	0.29
45	10.00	1.47	0.39	0.39	0.03	0.02	0.03	0.03	0.04	1.36
48	10.00	1.47	0.40	0.40	0.03	0.02	0.02	0.03	0.03	1.26
51	10.00	1.46	0.41	0.41	0.03	0.02	0.02	0.03	0.03	0.16
54	10.00	1.45	0.42	0.42	0.03	0.01	0.02	0.03	0.03	0.04
57	10.00	1.43	0.43	0.43	0.03	0.01	0.02	0.02	0.03	0.02
60	10.00	1.42	0.44	0.45	0.02	0.01	0.02	0.02	0.03	0.02
65	10.00	1.40	0.45	0.48	0.02	0.01	0.02	0.02	0.03	0.01
70	10.00	1.39	0.45	0.50	0.02	0.01	0.01	0.02	0.02	0.01
75	10.00	1.38	0.46	0.51	0.02	0.01	0.01	0.02	0.02	0.01
80	10.00	1.36	0.47	0.52	0.02	0.01	0.01	0.02	0.03	0.01
85	10.00	1.36	0.48	0.52	0.02	0.01	0.01	0.02	0.03	0.02
90	10.00	1.34	0.49	0.50	0.02	0.01	0.01	0.02	0.03	0.01
95	10.00	1.37	0.50	0.51	0.02	0.01	0.01	0.02	0.04	0.01
100	10.00	1.35	0.51	0.51	0.02	0.01	0.01	0.02	0.07	0.01
105	10.00	1.32	0.50	0.51	0.01	0.01	0.01	0.01	0.12	0.00
110	10.00	1.31	0.51	0.51	0.01	0.01	0.01	0.01	0.19	0.00
115	10.00	1.30	0.51	0.51	0.01	0.01	0.01	0.01	0.64	0.00
120	10.00	1.30	0.52	0.52	0.01	0.01	0.01	0.01	1.10	0.01

Table S5: Concentrations [mmol/L] for hexamethylbenzene standard, ligand (as a mixture of **1** and **1.HX**), **2** (green), intermediate A’ (blue), intermediate A’’ (yellow), intermediate B (red) of the experiment at 75 °C.

time [min]	Standard	**1** and **1.HX**	**2** (4.11 ppm)	**2** (3.97 ppm)	4.26 ppm	4.23 ppm	4.21 ppm	3.63 ppm	3.57 ppm	3.47 ppm
0	38.16	27.03	0.51	0.51	1.03	0.83	0.31	0.26	1.17	1.02
3	38.16	25.28	1.13	1.12	1.37	1.00	0.56	0.51	1.65	1.24
6	38.16	23.82	1.85	1.84	1.56	1.08	0.77	0.97	1.61	1.19
9	38.16	22.63	2.60	2.58	1.61	1.09	0.92	0.90	1.67	1.22
12	38.16	21.62	3.35	3.33	1.60	1.06	1.01	0.98	1.67	1.08
15	38.16	20.70	4.09	4.06	1.58	1.03	1.04	1.03	1.63	1.01
18	38.16	19.98	4.76	4.74	1.52	0.96	1.03	1.04	1.56	0.95
21	38.16	19.39	5.42	5.39	1.42	0.88	1.01	1.03	1.47	0.86
24	38.16	18.90	6.01	5.98	1.34	0.83	0.97	0.99	1.56	0.81
27	38.16	18.53	6.55	6.53	1.24	0.75	0.91	0.95	1.31	0.77
30	38.16	18.09	7.05	7.03	1.16	0.68	0.85	0.90	1.24	0.73
33	38.16	17.78	7.50	7.47	1.08	0.64	0.79	0.87	1.19	0.70
36	38.16	17.46	7.92	7.90	1.02	0.58	0.74	0.82	1.05	0.80
39	38.16	17.28	8.27	8.25	0.92	0.52	0.68	0.84	0.95	1.05
42	38.16	17.10	8.61	8.58	0.86	0.47	0.63	0.88	0.90	6.61
45	38.16	16.86	8.91	8.89	0.80	0.43	0.59	0.74	0.84	31.20
48	38.16	16.88	9.18	9.17	0.74	0.39	0.53	0.67	0.77	28.96
51	38.16	16.75	9.44	9.43	0.69	0.36	0.50	0.64	0.73	3.77
54	38.16	16.54	9.64	9.66	0.64	0.32	0.46	0.60	0.69	0.93
57	38.16	16.35	9.83	9.89	0.58	0.29	0.41	0.56	0.65	0.51
60	38.16	16.23	10.03	10.30	0.56	0.29	0.39	0.55	0.62	0.36
65	38.16	16.08	10.19	11.08	0.51	0.26	0.36	0.54	0.58	0.33
70	38.16	15.95	10.34	11.48	0.48	0.24	0.33	0.51	0.56	0.31
75	38.16	15.78	10.53	11.68	0.46	0.24	0.31	0.49	0.56	0.21
80	38.16	15.58	10.71	11.80	0.41	0.22	0.28	0.46	0.59	0.19
85	38.16	15.54	10.98	11.88	0.45	0.28	0.34	0.47	0.61	0.35
90	38.16	15.38	11.11	11.45	0.42	0.28	0.31	0.43	0.72	0.32
95	38.16	15.64	11.51	11.65	0.39	0.27	0.29	0.37	0.88	0.31
100	38.16	15.48	11.63	11.69	0.40	0.29	0.30	0.35	1.53	0.28
105	38.16	15.14	11.56	11.60	0.28	0.15	0.18	0.27	2.73	0.10
110	38.16	14.98	11.63	11.66	0.27	0.14	0.16	0.24	4.31	0.10
115	38.16	14.85	11.71	11.71	0.27	0.14	0.16	0.21	14.67	0.07
120	38.16	14.85	11.84	11.86	0.24	0.12	0.14	0.17	25.08	0.12

Table S6: Overlap-corrected concentrations [mmol/L] for hexamethylbenzene standard, ligand (as a mixture of **1** and **1.HX**), **2** (green), intermediate A' (blue), intermediate A'' (yellow), intermediate B (red) of the experiment at 75 °C. Overlap corrected concentrations are marked in light blue.

time [min]	Standard	**1** and **1.HX**	**2** (4.11 ppm)	**2** (3.97 ppm)	4.26 ppm	4.23 ppm	4.21 ppm	3.63 ppm	3.57 ppm	3.47 ppm
0	38.16	27.03	0.51	0.51	1.03	0.83	0.31	0.26	1.17	0.83
3	38.16	25.28	1.13	1.12	1.37	1.00	0.56	0.51	1.65	1.00
6	38.16	23.82	1.85	1.84	1.56	1.08	0.77	0.97	1.61	1.08
9	38.16	22.63	2.60	2.58	1.61	1.09	0.92	0.90	1.67	1.09
12	38.16	21.62	3.35	3.33	1.60	1.06	1.01	0.98	1.67	1.06
15	38.16	20.70	4.09	4.06	1.58	1.03	1.04	1.03	1.63	1.01
18	38.16	19.98	4.76	4.74	1.52	0.96	1.03	1.04	1.56	0.95
21	38.16	19.39	5.42	5.39	1.42	0.88	1.01	1.03	1.47	0.86
24	38.16	18.90	6.01	5.98	1.34	0.83	0.97	0.99	1.56	0.81
27	38.16	18.53	6.55	6.53	1.24	0.75	0.91	0.95	1.31	0.77
30	38.16	18.09	7.05	7.03	1.16	0.68	0.85	0.90	1.24	0.73
33	38.16	17.78	7.50	7.47	1.08	0.64	0.79	0.79	1.19	0.70
36	38.16	17.46	7.92	7.90	1.02	0.58	0.74	0.74	1.05	0.58
39	38.16	17.28	8.27	8.25	0.92	0.52	0.68	0.68	0.95	0.52
42	38.16	17.10	8.61	8.58	0.86	0.47	0.63	0.63	0.90	0.47
45	38.16	16.86	8.91	8.89	0.80	0.43	0.59	0.59	0.84	0.43
48	38.16	16.88	9.18	9.17	0.74	0.39	0.53	0.53	0.77	0.39
51	38.16	16.75	9.44	9.43	0.69	0.36	0.50	0.50	0.73	0.36
54	38.16	16.54	9.64	9.66	0.64	0.32	0.46	0.46	0.69	0.32
57	38.16	16.35	9.83	9.83	0.58	0.29	0.41	0.41	0.65	0.29
60	38.16	16.23	10.03	10.03	0.56	0.29	0.39	0.39	0.62	0.36
65	38.16	16.08	10.19	10.19	0.51	0.26	0.36	0.36	0.58	0.33
70	38.16	15.95	10.34	10.34	0.48	0.24	0.33	0.33	0.56	0.31
75	38.16	15.78	10.53	10.53	0.46	0.24	0.31	0.31	0.56	0.21
80	38.16	15.58	10.71	10.71	0.41	0.22	0.28	0.28	0.59	0.19
85	38.16	15.54	10.98	10.98	0.45	0.28	0.34	0.34	0.61	0.35
90	38.16	15.38	11.11	11.11	0.42	0.28	0.31	0.31	0.42	0.32
95	38.16	15.64	11.51	11.51	0.39	0.27	0.29	0.29	0.39	0.31
100	38.16	15.48	11.63	11.63	0.40	0.29	0.30	0.30	0.40	0.28
105	38.16	15.14	11.56	11.56	0.28	0.15	0.18	0.18	0.28	0.10
110	38.16	14.98	11.63	11.63	0.27	0.14	0.16	0.16	0.27	0.10
115	38.16	14.85	11.71	11.71	0.27	0.14	0.16	0.16	0.27	0.07
120	38.16	14.85	11.84	11.86	0.24	0.12	0.14	0.14	0.24	0.12

Fitting of concentrations

Concentrations were fitted in Python using Anaconda[S3] and SciPy.[S4] Rate constants were calculated using a least square regression for the concentrations of intermediates **A**, **B** and product **2**. Input files including residual analysis, profile likelihood and correlation matrix for the parameters are compiled at the end of the supporting information. Both k_1 and k_4 show a strong correlation to each other, which can be is explained by the fact that both deplete the same free ligand. In addition to that there has to be some correlation as the major side product generated by k_4 (protonated ligand) is expected to come from the HCl generated from the cyclometalation which proceeds over k_1.

Table S7: Calculated rate constants

Temperature	60 °C	75 °C
k_1	$10^{-4.1}$ s^{-1}	$10^{-3.6}$ s^{-1}
k_2	$10^{-3.3}$ s^{-1}	$10^{-2.8}$ s^{-1}
k_3	$10^{-2.9}$ s^{-1}	$10^{-2.2}$ s^{-1}
k_4	$10^{-3.9}$ s^{-1}	$10^{-3.2}$ s^{-1}

4. Synthesis of Complex 2 with 2 equiv. of bisPYA ligand

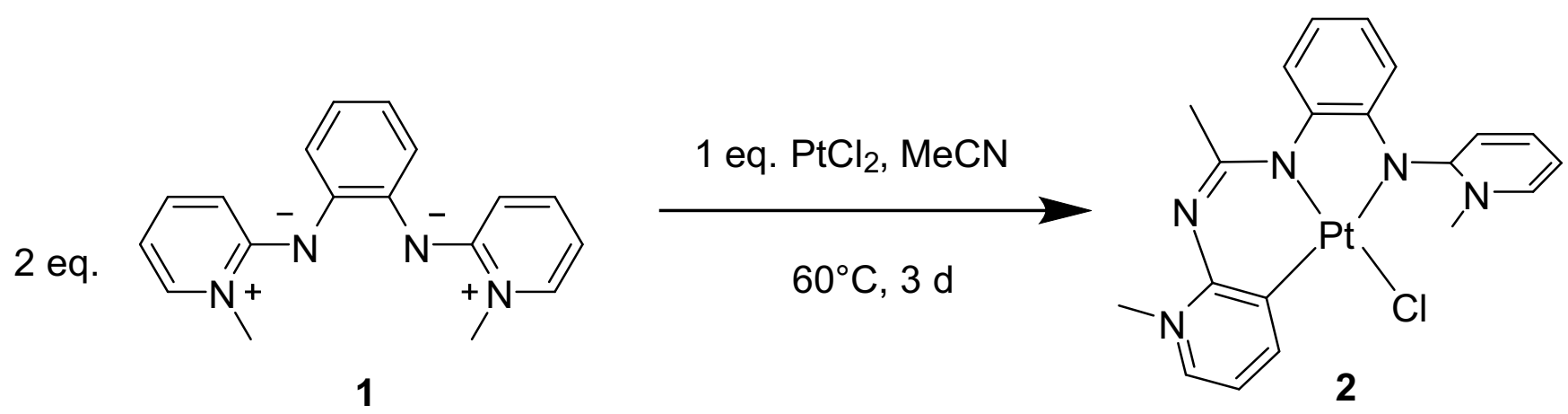

To a vial with $PtCl_2$ (100 mg, 0.37 mmol, 1.0 equiv.) and hexamethylbenzene (40 mg, 0.25 mmol, 0.66 equiv.) as standard dry MeCN (10 mL) was added. The mixture was heated to 80 °C and stirred for 20 min until a clear yellow solution formed. A solution of bisPYA **1** (240 mg, 0.74 mmol) dissolved in dry MeCN (10 mL) was added. The mixture was stirred and heated to 80 °C for 7 h, resulting in a dark red solution. The mixture was dried in vacuo, dissolved in CH_2Cl_2 (5 ml). 0.2ml of the solution was dried in vacuo and analyzed by ^{1}H-NMR in CH_2Cl_2 (67 % yield of **2** relative to platinum observed). The rest of the solution was dried in vacuo to yield a dark red-brown solid (368 mg), which resolved into a mixture of product **2** and free ligand upon addition of *t*BuOK (Fig. S9), supporting the formation of **1-H.X** as the by-product upon formation of **2**.

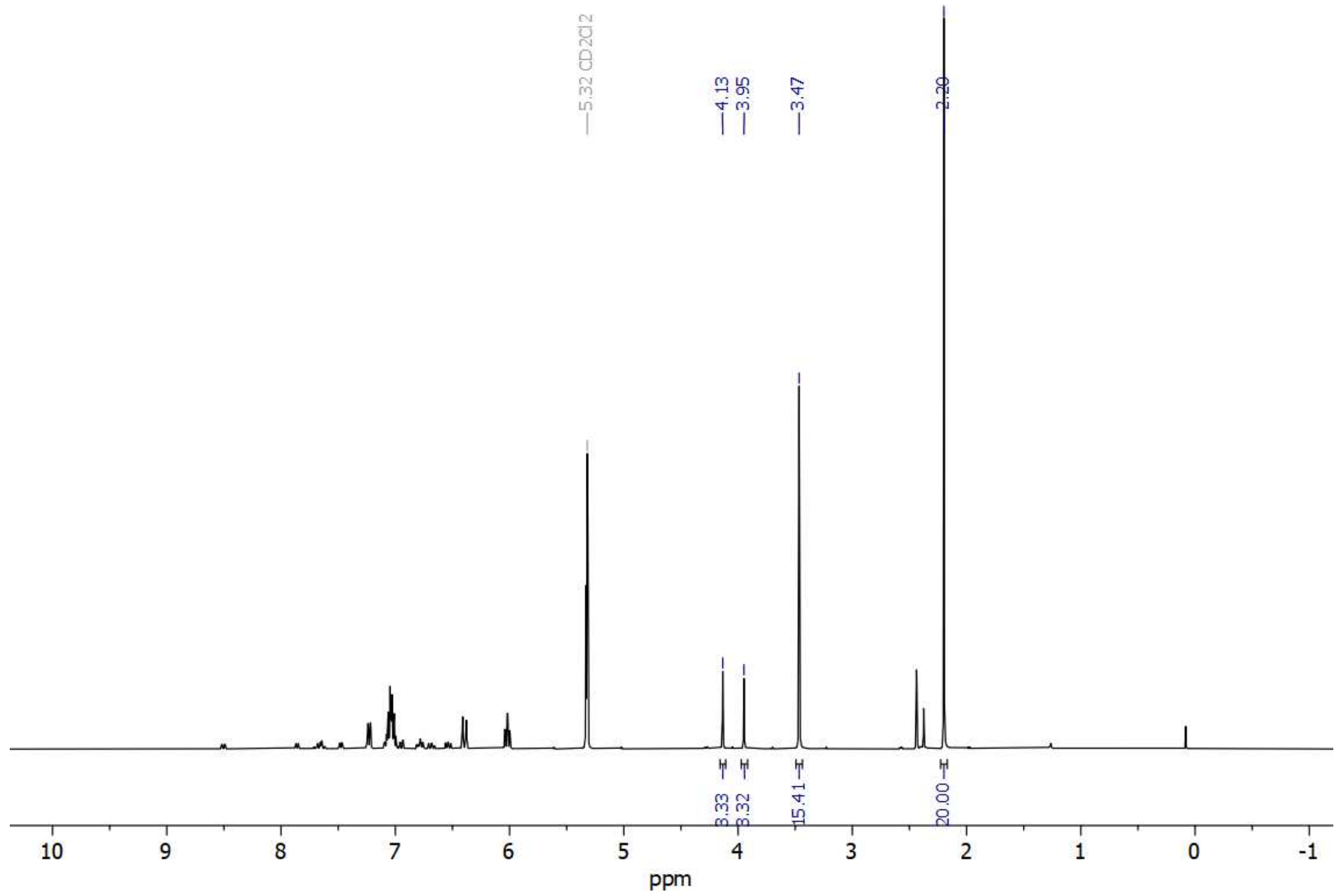

Figure S8: ^{1}H NMR spectra of the product mixture with 2 equivalent ligand and hexamethylbenzene as standard (δ_H = 2.20)

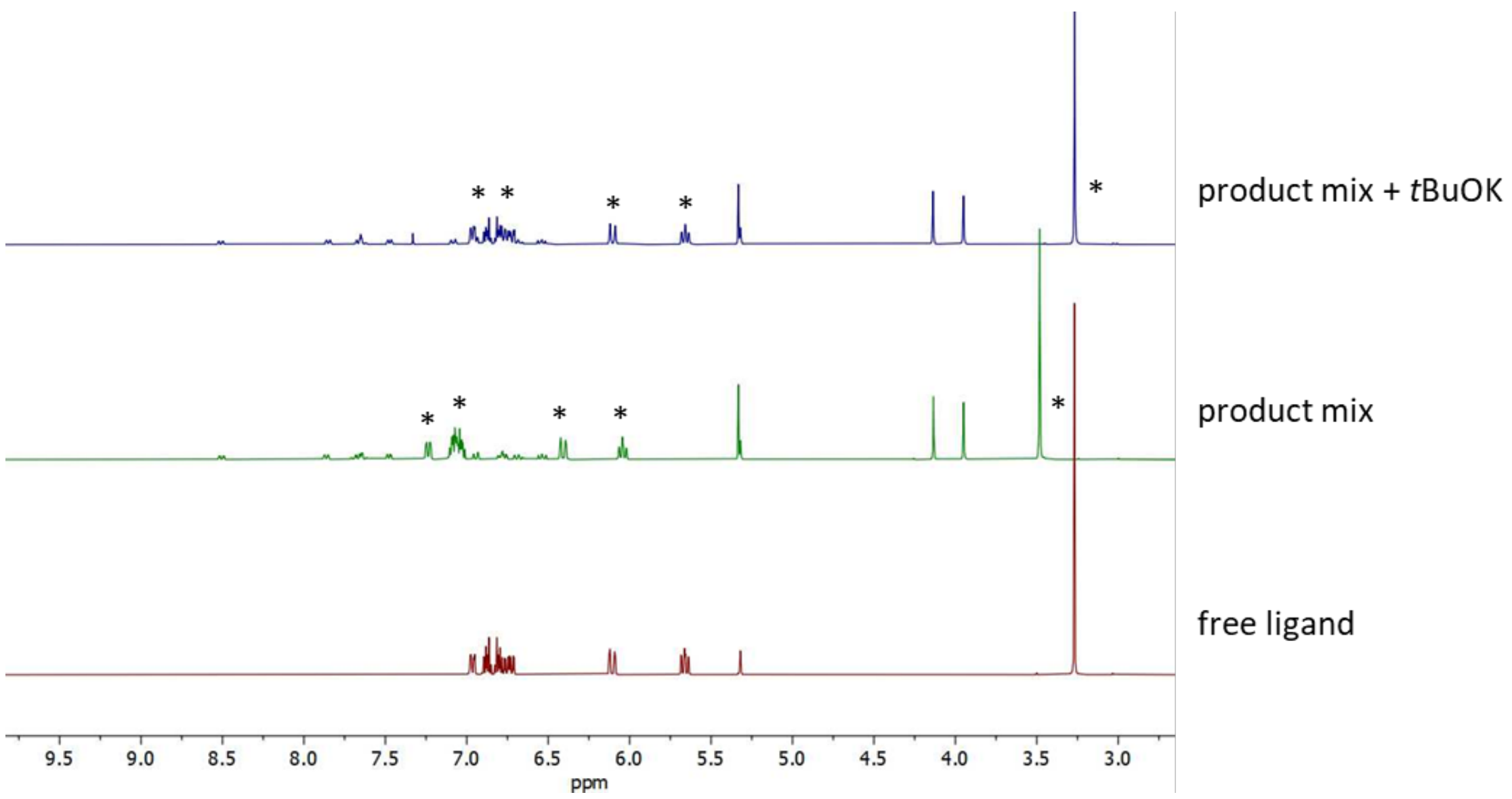

Figure S9: ^{1}H NMR spectra of the free bisPYA ligand **1**, 2 equiv. ligand product mixture and mixture with excess tBuOK added. * = shifting protons not belonging to the cyclometalated species **2**

5. X-Ray Crystallographic Data

Crystal-Structure Determination. Suitable single crystals of **2**, **3** and **4** were immersed in parabar oil and mounted at ambient conditions, then transferred into the stream of nitrogen (100 K for **3** and **4**, 173 K for **2**). All measurements for **2** were made on a *RIGAKU XtaLAB Synergy R, HyPix-Arc 100* Detector diffractometer[S5] using mirror optics monochromated MoK_{α} radiation (λ = 0.71073 Å). Crystals of **3** and **4** were measured on a *RIGAKU Synergy S* area-detector diffractometer[S5] using mirror optics monochromated Cu *K*α radiation (λ = 1.54184 Å).

Data reduction was performed using the *CrysAlisPro*[S5] program. The intensities were corrected for Lorentz and polarization effects, and an analytical numeric absorption correction using a multifaceted crystal model[S6] with additional empirical absorption correction using spherical harmonics, as implemented in SCALE3 ABSPACK scaling algorithm in *CrysAlisPro*[S5] was applied.

The structures were solved by intrinsic phasing using *SHELXT,*[S7] which revealed the positions of all non-hydrogen atoms. All non-hydrogen atoms were refined anisotropically. H-atoms were assigned in geometrically calculated positions and refined using a riding model where each H-atom was assigned a fixed isotropic displacement parameter with a value equal to 1.2U_{eq} of its parent atom (1.5U_{eq} for methyl groups).

Refinement of the structures was carried out on F^2 using full-matrix least-squares procedures, which minimized the function $\Sigma w(F_o^2 - F_c^2)^2$. The weighting scheme was based on counting statistics and included a factor to downweight the intense reflections. All calculations were performed using the *SHELXL-2014/7*[S8] program in OLEX2.[S9]

Disorder model was used for parts of the structure of **4** where the occupancies of each disorder component was refined through the use of a free variable. The sum of equivalent components was constrained to 1, i.e. 100%. Final occupancies are listed in the associated crystallographic data. Data collection and refinement parameters are given in Tables S8 and S9, and selected bond lengths and angles in Tables S10 and S11. Crystallographic data for all structures reported in this paper have been deposited with the Cambridge Crystallographic Data Centre (CCDC) as supplementary publication numbers 2571090–2571092. The X-ray crystal structure determination service unit of the Department of Chemistry and Biochemistry of the University of Bern is acknowledged for measuring, solving, refining and summarizing the structures with equipment partially funded by the Swiss National Science Foundation (SNSF; grant 206021_177033).

Table S8: Crystal data and structure refinement for complexes **2** and **3**

CCDC No.	2571092	2571091
Empirical formula	$C_{41}H_{42}Cl_4N_{10}Pt_2$	$C_{43}H_{46}Cl_4N_{10}Pt_2$
Formula weight	1206.82	1234.88
Temperature/K	173.00(10)	100.01(10)
Crystal system	triclinic	triclinic
Space group	P–1	P–1
a/Å	9.7265(2)	9.45802(5)
b/Å	11.3337(2)	11.57010(7)
c/Å	18.8958(3)	20.01668(10)
α/°	85.2570(10)	80.9996(4)
β/°	80.6350(10)	83.9421(4)
γ/°	87.5690(10)	87.5430(5)
Volume/$Å^3$	2047.33(6)	2150.60(2)
Z	2	2
ρ_{calc}g/cm^3	1.958	1.907
μ/mm^{-1}	7.132	14.636
F(000)	1164.0	1196.0
Crystal size/mm^3	0.318 × 0.177 × 0.1	0.19 × 0.147 × 0.035
Radiation	Mo Kα (λ = 0.71073)	Cu Kα (λ = 1.54184)
2Θ range for data collection/°	3.608 to 59.996	7.74 to 148.964
Index ranges	–13 ≤ h ≤ 13, –15 ≤ k ≤ 15, –26 ≤ l ≤ 26	–11 ≤ h ≤ 11, –13 ≤ k ≤ 14, –24 ≤ l ≤ 25
Reflections collected	123056	88258
Independent reflections	11931 [R_{int} = 0.0542, R_{sigma} = 0.0212]	8777 [R_{int} = 0.0279, R_{sigma} = 0.0119]
Data/restraints/parameters	11931/0/514	8777/0/539
Goodness-of-fit on F^2	1.083	1.092
Final R indexes [I≥2σ (I)]	R_1 = 0.0236, wR_2 = 0.0598	R_1 = 0.0209, wR_2 = 0.0557
Final R indexes [all data]	R_1 = 0.0272, wR_2 = 0.0612	R_1 = 0.0211, wR_2 = 0.0559
Largest diff. peak/hole / e $Å^{-3}$	2.10/–0.99	1.46/–0.97

Table S9: Crystal data and structure refinement for **4**

CCDC No.	2571090
Empirical formula	$C_{26}H_{24}Cl_3N_5Pt$
Formula weight	707.94
Temperature/K	99.99(10)
Crystal system	monoclinic
Space group	$P2_1$
a/Å	11.66920(8)
b/Å	8.98058(6)
c/Å	12.40714(9)
α/°	90
β/°	91.7792(6)
γ/°	90
Volume/Å^3	1299.594(15)
Z	2
ρ_{calc}g/cm^3	1.809
μ/mm^{-1}	13.133
F(000)	688.0
Crystal size/mm^3	0.218 × 0.126 × 0.049
Radiation	Cu Kα (λ = 1.54184)
2Θ range for data collection/°	7.128 to 148.902
Index ranges	–14 ≤ h ≤ 14, –10 ≤ k ≤ 11, –15 ≤ l ≤ 15
Reflections collected	52630
Independent reflections	5290 [R_{int} = 0.0451, R_{sigma} = 0.0167]
Data/restraints/parameters	5290/17/329
Goodness-of-fit on F^2	1.112
Final R indexes [I≥2σ (I)]	R_1 = 0.0189, wR_2 = 0.0514
Final R indexes [all data]	R_1 = 0.0190, wR_2 = 0.0514
Largest diff. peak/hole / e Å^{-3}	0.96/-0.72
Flack parameter	-0.033(5)

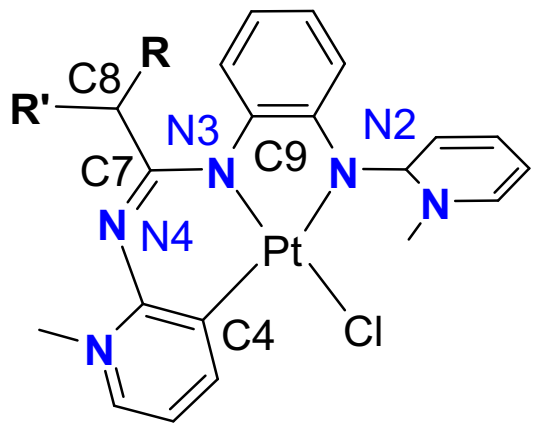

Table S10: Selected bond lenghts (Å) around the platinum center and the inserted –CN– fragment (N4, C7, C8) of complexes **2**, **3** and **4**

Bond	Complex **2**	Complex **3**	Complex **4**
Pt–Cl	2.3330(8)	2.3266(7)	2.3393(11)
Pt–N4	2.142(3)	2.134(2)	2.124(3)
Pt–N3	1.983(3)	1.993(2)	1.988(4)
Pt–C4	1.985(3)	1.982(3)	1.987(4)
N3–C9	1.415(4)	1.414(4)	1.434(6)
N3–C7	1.342(4)	1.336(4)	1.323(6)
N4–C7	1.328(4)	1.330(4)	1.337(6)
C7–C8	1.507(5)	1.523(4)	1.497(7)

Table S11: Selected bond angles (deg) around the platinum center and the inserted –CN– fragment (N4, C7, C8) of complexes **2**, **3** and **4**

angle	Complex **2**	Complex **3**	Complex **4**
N3–Pt–Cl	174.83(8)	95.48(7)	174.86(11)
N3–Pt–N2	80.22(10)	175.09(7)	80.36(15)
N3–Pt–C4	90.93(12)	80.07(10)	94.60(11)
N2–Pt–Cl	97.06(7)	92.36(8)	93.26(15)
C4–Pt–Cl	92.17(10)	92.25(11)	91.69(18)
C4–Pt–N2	169.65(12)	170.89(10)	171.11(17)
C7–N2–C5	122.9(3)	124.5(3)	122.7(4)
C7–N3–Pt	121.6(2)	122.20(19)	123.1(3)
C9–N3–Pt	114.2(2)	114.01(18)	112.7(3))
N4–C7–N3	125.2(3)	124.4(3)	126.1(4)
N3–C7–C8	121.9(3)	121.8(3)	121.0(4)
N4–C7–C8	112.9(3)	113.7(2)	112.9(4)

6. NMR Spectra

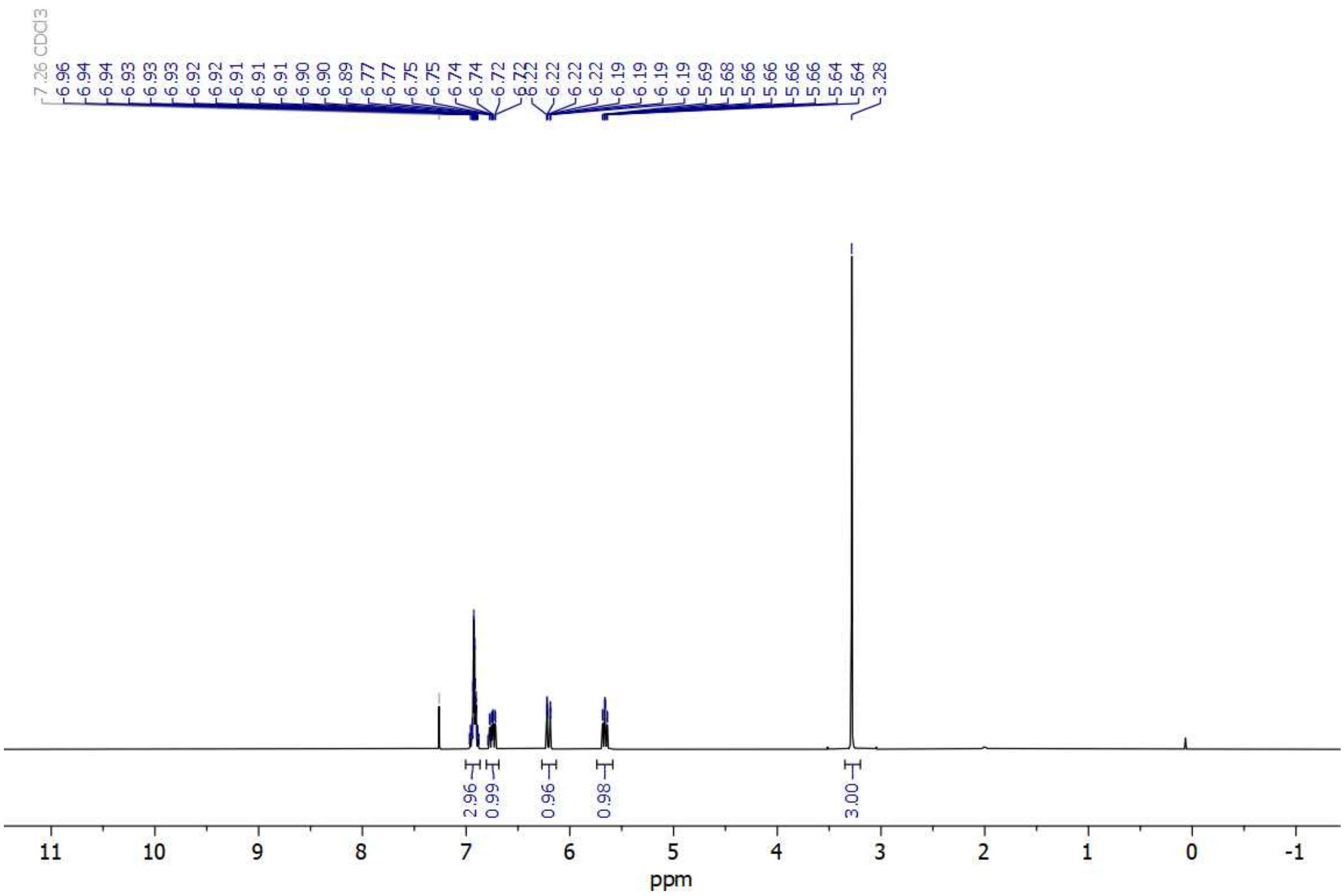

Figure S9: ^{1}H NMR (300 MHz) spectrum of bisPYA **(1)** in $CDCl_3$

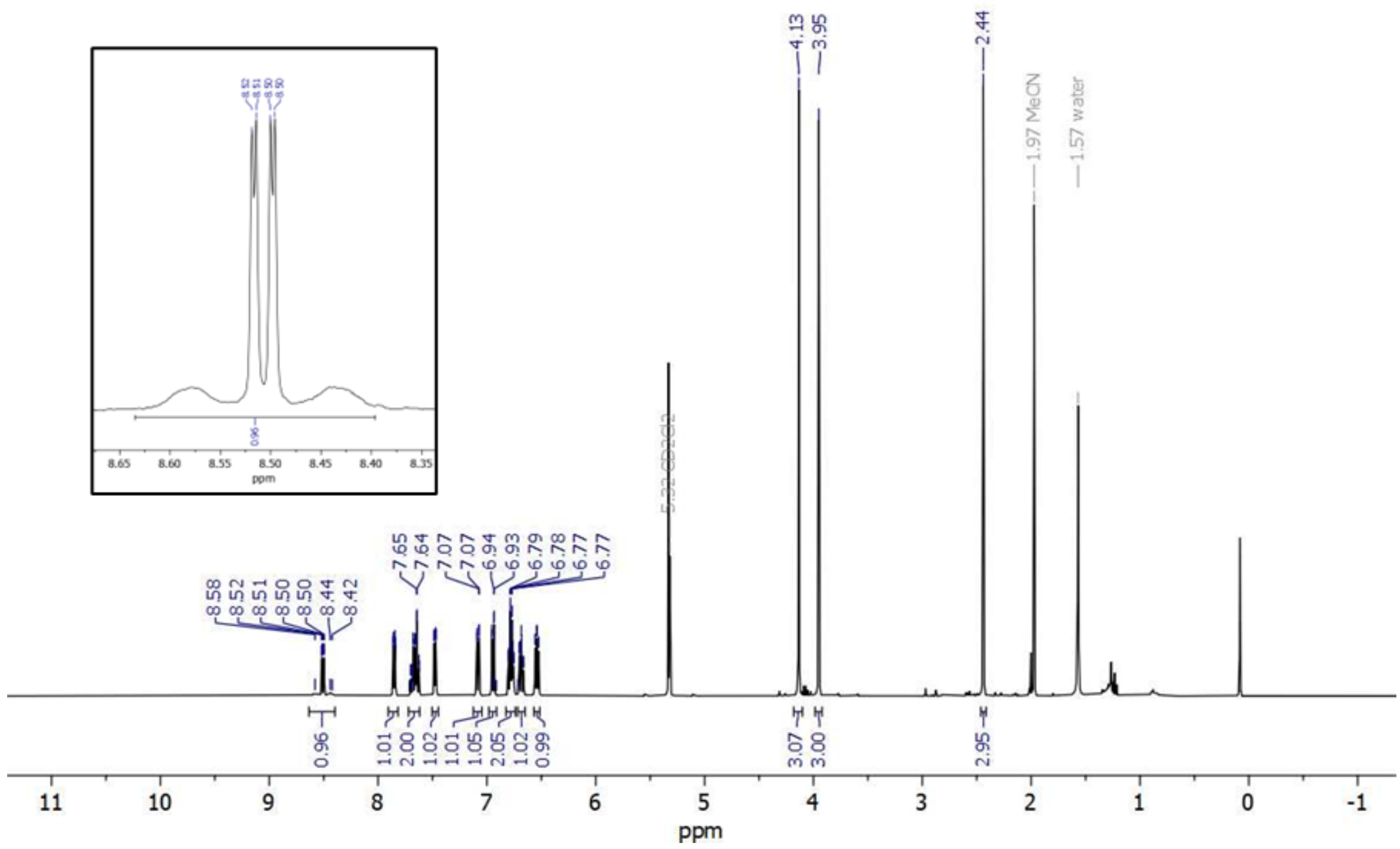

Figure S10: ^{1}H NMR (400 MHz) spectrum of complex **2** in CD_2Cl_2

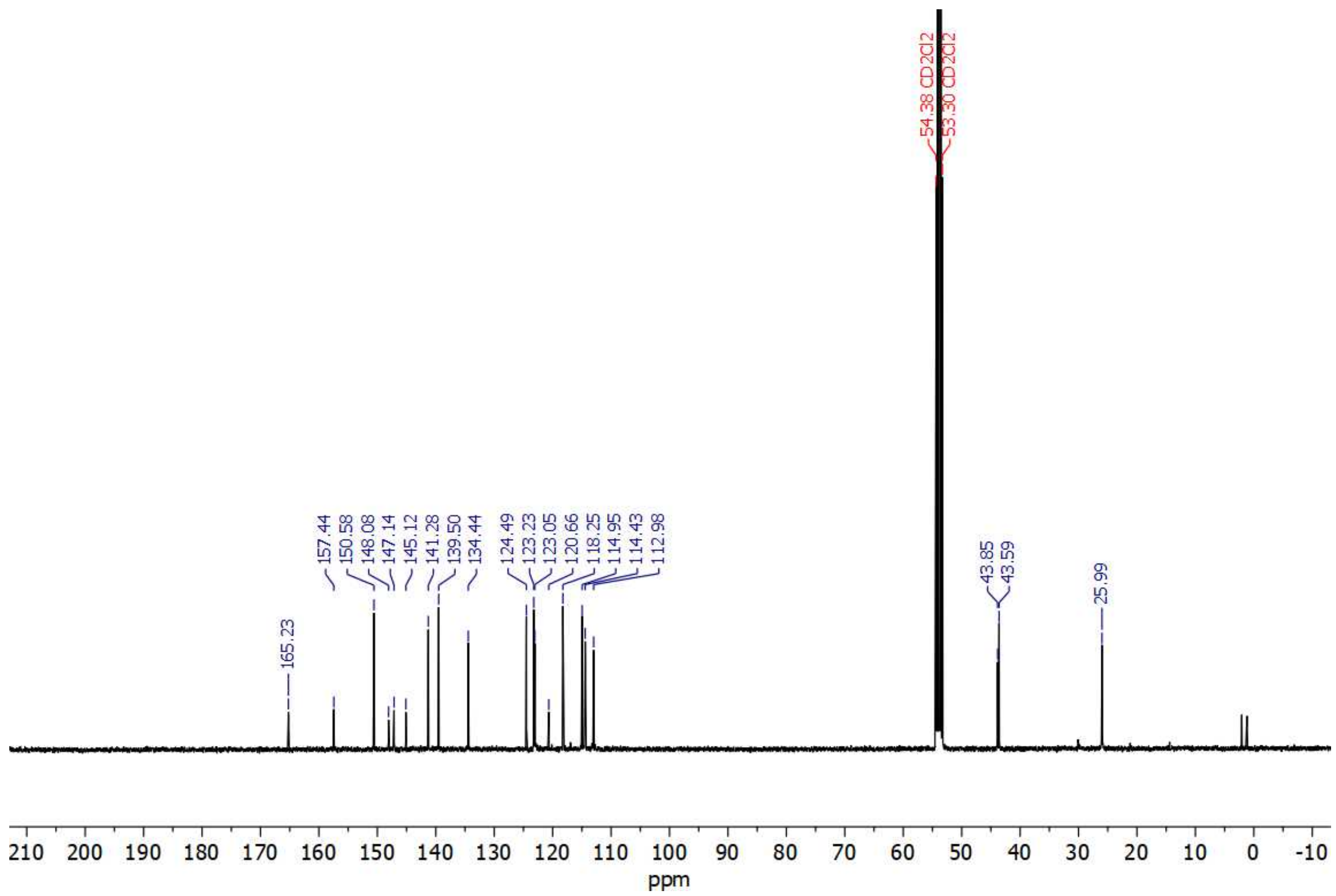

Figure S11: $^{13}C\{^1H\}$ (100 MHz) NMR spectrum of complex **2** in CD_2Cl_2

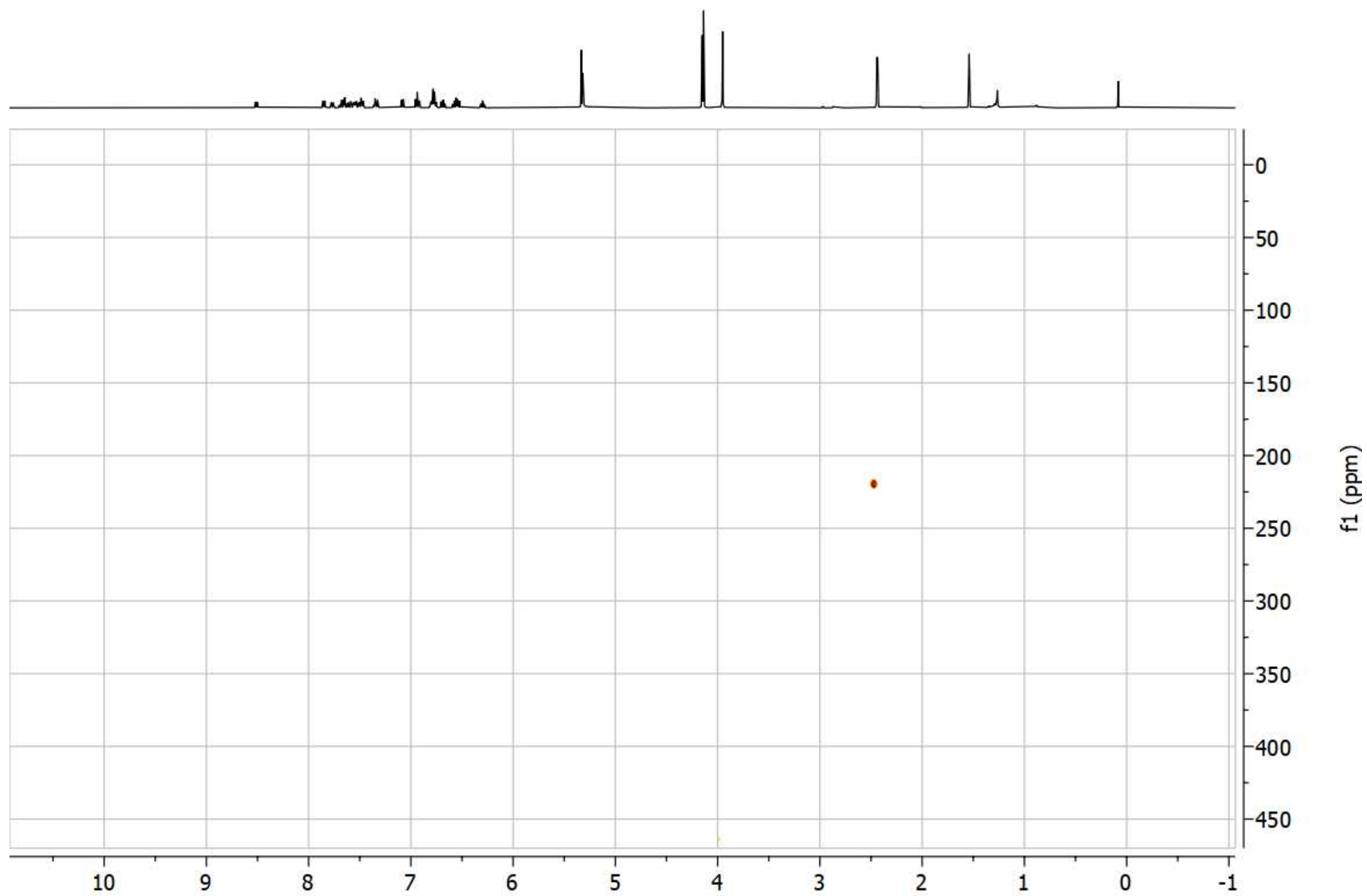

Figure S12: ^1H-^{15}N HSQC (400 MHz, 40 MHz) spectrum of complex **2-^{15}N** in CD_2Cl_2

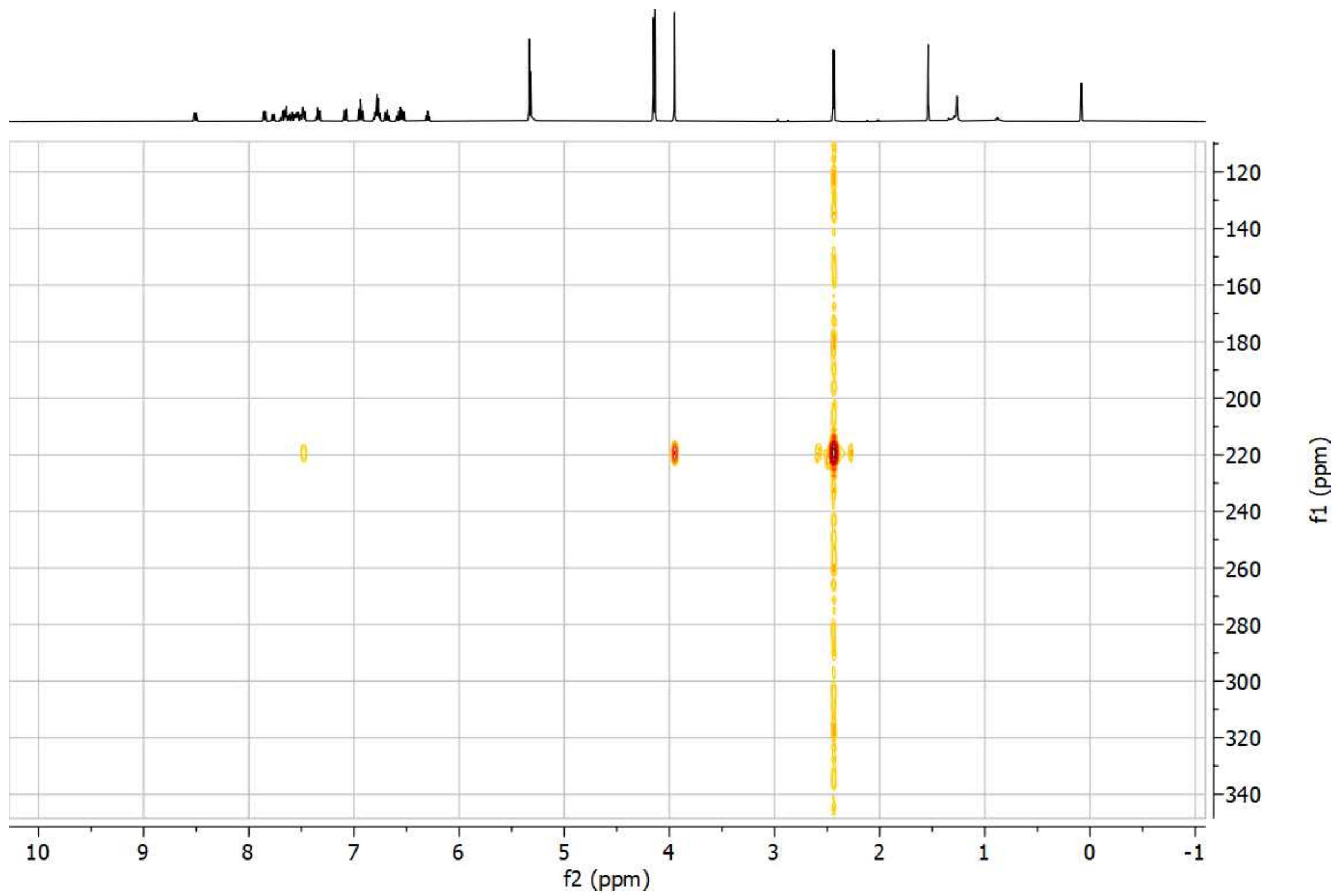

Figure S13: ^{1}H-^{15}N HMBC (400 MHz, 40 MHz) spectrum of complex **2-^{15}N** in CD_2Cl_2

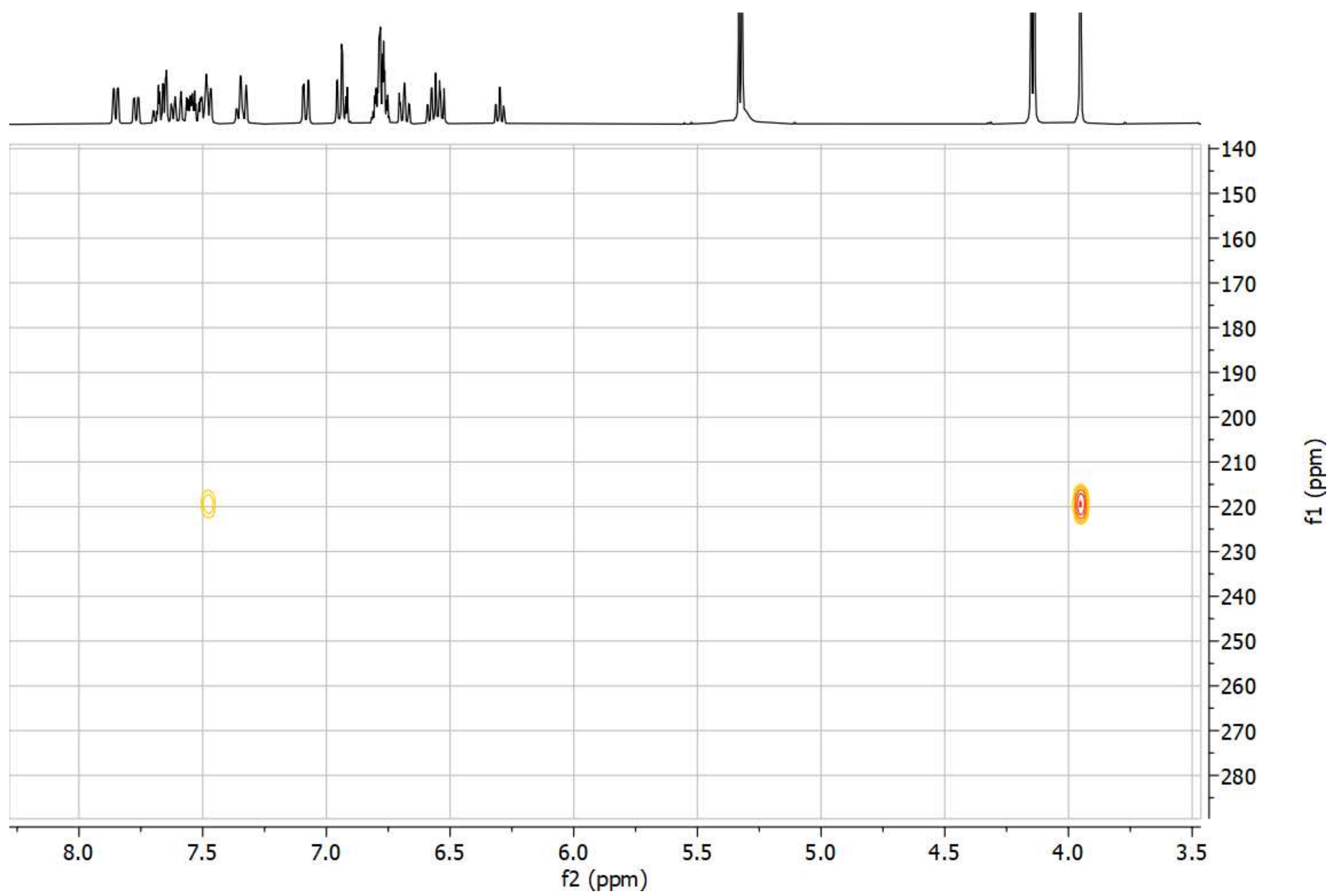

Figure S14: ^{1}H-^{15}N HMBC (400 MHz, 40 MHz) spectrum of complex **2-^{15}N** in CD_2Cl_2

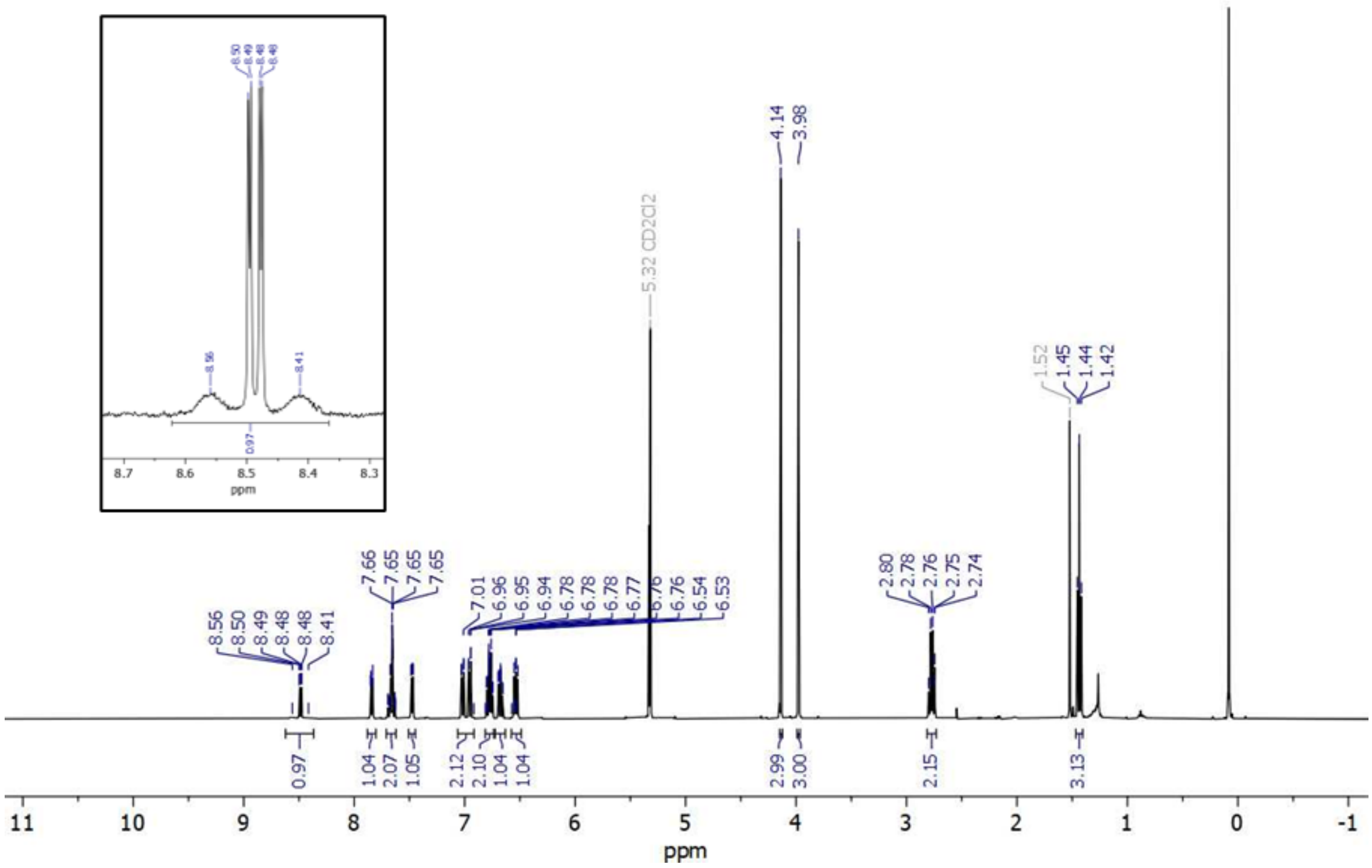

Figure S15: ^{1}H NMR (400 MHz) spectrum of complex **3** in CD_2Cl_2

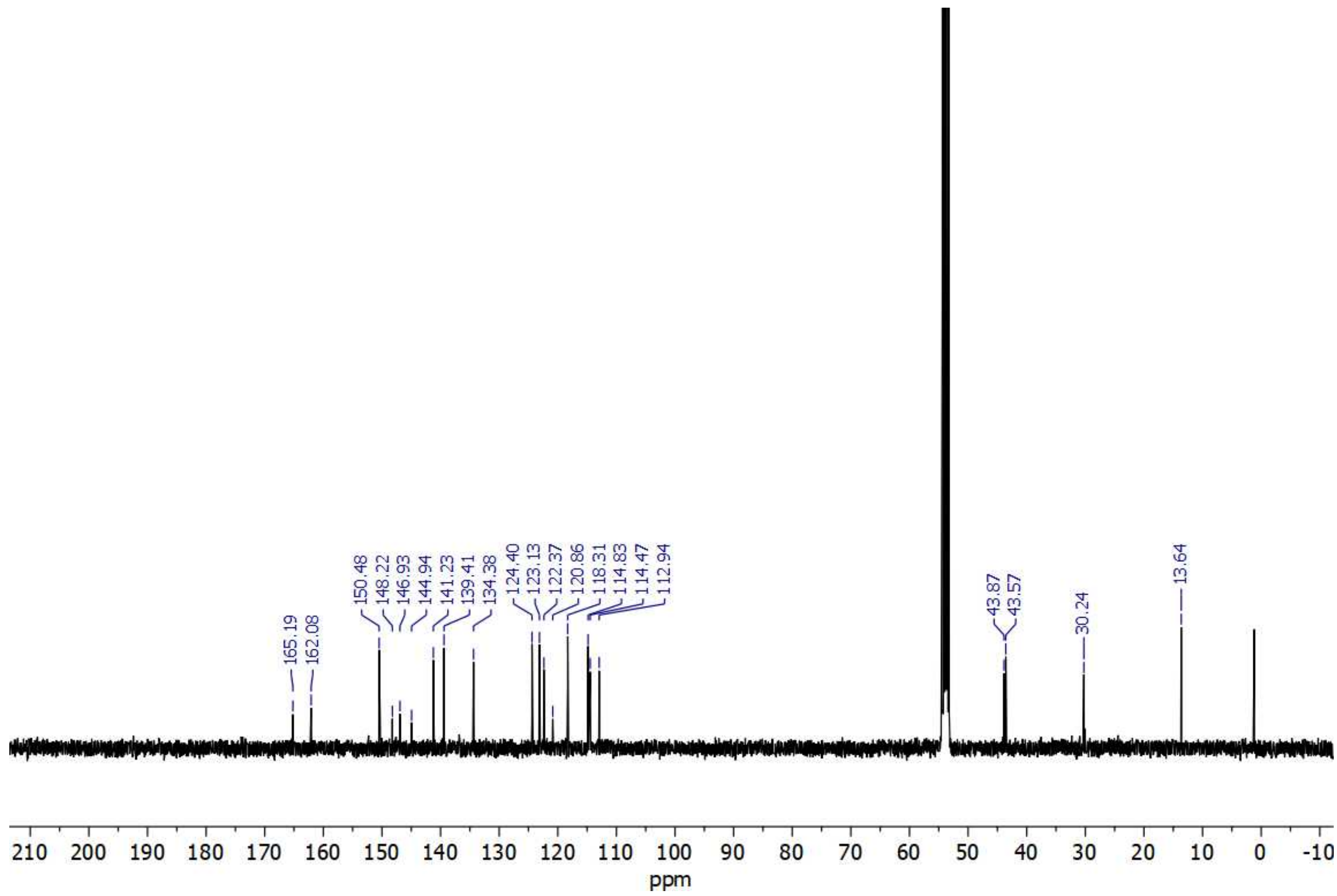

Figure S16: $^{13}C\{^1H\}$ (100 MHz) NMR spectrum of complex **3** in CD_2Cl_2

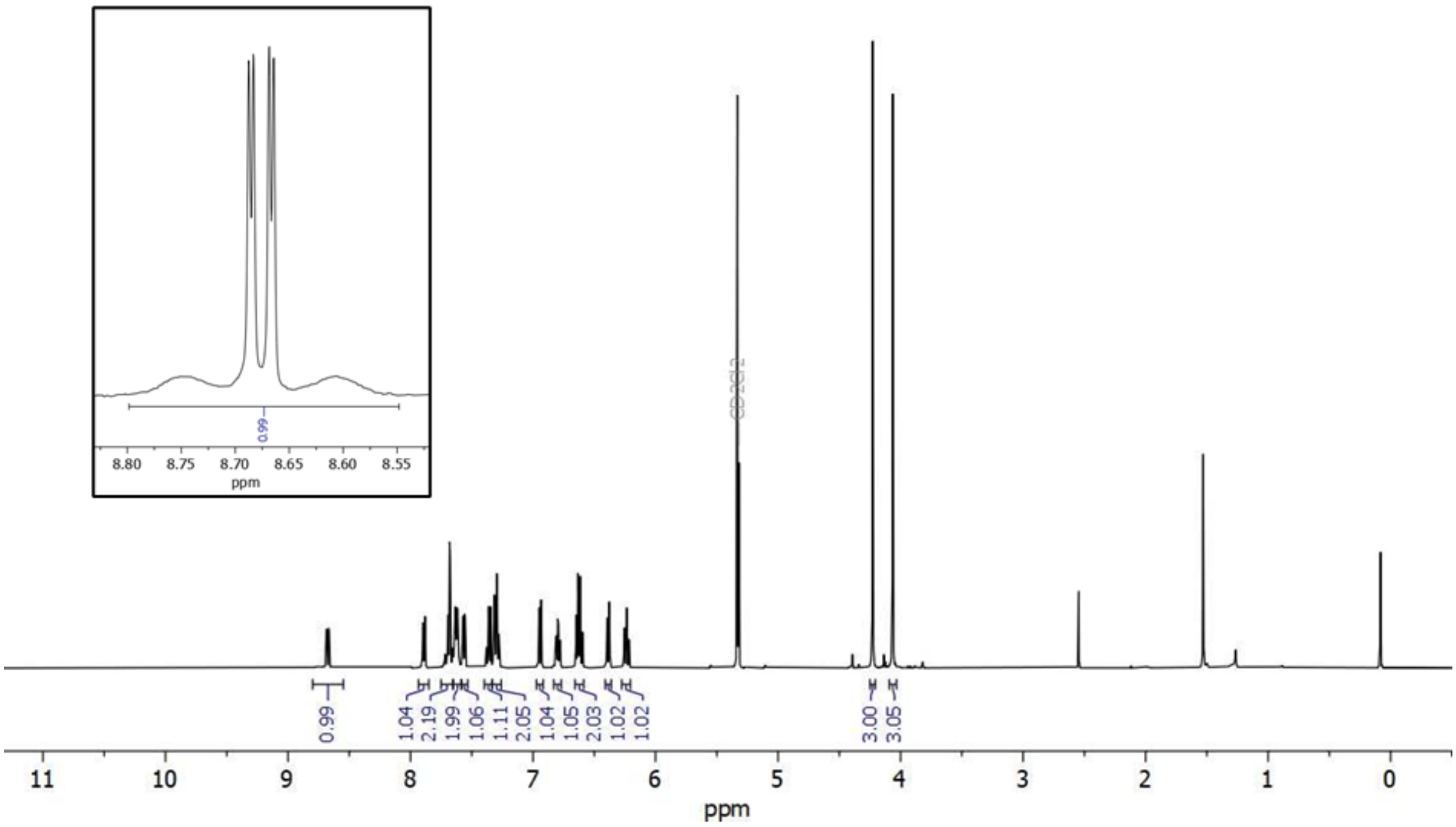

Figure S17: ^{1}H NMR (400 MHz) spectrum of complex **4** in CD_2Cl_2

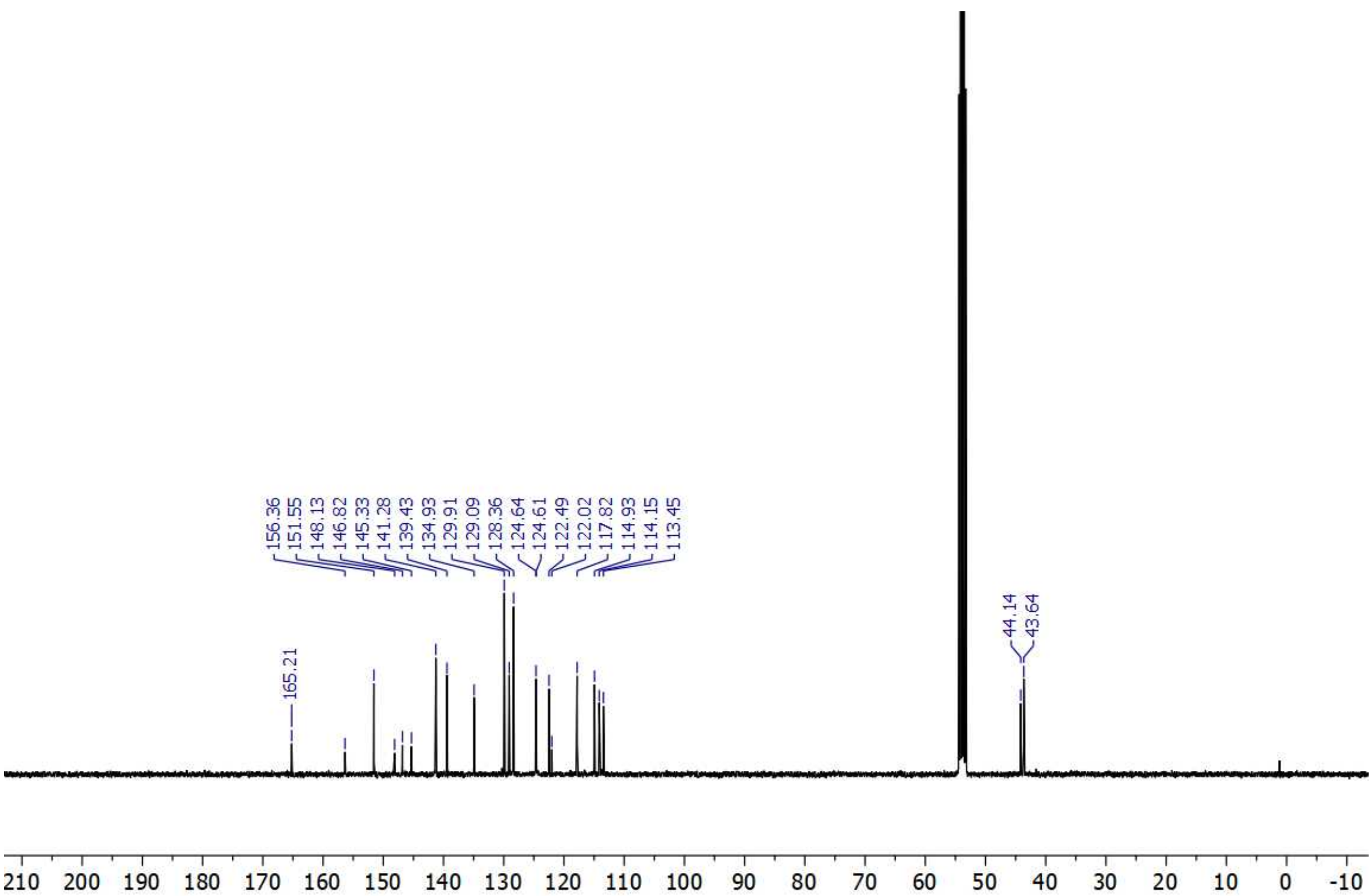

Figure S18: $^{13}C\{^{1}H\}$ (100 MHz) NMR spectrum of complex **4** in CD_2Cl_2

7. References

S1 N. Munir, S. Masood, F. Liaqat, M. N. Tahir, S. Yousuf, S. Kalsoom, E. U. Mughal, S. H. Sumrra, A. Maalik and M. N. Zafar, *RSC Adv.*, 2019, **9**, 37986–38000.

S2 N. S. Y. Abdolla, D. L. Davies and K. Singh, *European Journal of Inorganic Chemistry*, 2021, **2021**, 939–950.

S3 Inc. Anaconda Software Distribution, Version 2024.10, 2024. https://www.anaconda.com/.

S4 Jones, E.; Oliphant, T.; Peterson, P.; others. SciPy: Open Source Scientific Tools for Python. 2001

S5 Oxford Diffraction (2018). CrysAlisPro (Version 1.171.40.37a). Oxford Diffraction Ltd., Yarnton, Oxfordshire, UK.

S6 R. C. Clark and J. S. Reid, *Acta Crystallographica Section A*, 1995, **51**, 887–897.

S7 G. M. Sheldrick, *Acta Cryst.*, 2015, **A71**, 3–8.

S8 G. M. Sheldrick, *Acta Cryst.*, 2015, **C71**, 3–8.

S9 O. V. Dolomanov, L. J. Bourhis, R. J. Gildea, J. A. K. Howard and H. Puschmann, *Journal of Applied Crystallography*, 2009, **42**, 339–341.

Kinetic Model Complex 2 Formation 60 deg

In [50]:
```
import numpy as np
import pandas as pd
import matplotlib.pyplot as plt
import math
```

NMR Data

In [53]:
```
#Data arrays, c in mmol/L, first concentration values for t = 0 (in dead time) was fixed at added starting conditions
Time = np.array([-3,
0,
10,
20,
30,
40,
50,
60,
70,
80,
90,
100,
110,
120,
130,
140,
150,
160,
170,
180,
200,
220,
240,
260,
280,
300,
320,
340,
360,
380,
400,
420])

Time = Time + 3


Free_Lig_corr = np.array([54.35,
38.5253084,
34.3852352,
31.4210228,
29.0700608,
27.3994472,
26.2138804,
25.2003208,
24.3998712,
23.8322812,
23.4797424,
23.4529032,
23.7718508,
23.8555756,
23.7746782,
23.9304595,
23.6135375,
23.62163146,
23.56828222,
23.7177504,
23.2446884,
23.2607244,
22.9456592,
23.14248,
22.9865088,
22.9486132,
22.7869028,
22.7117868,
22.5096488,
22.5519332,
22.2085096,
21.9742152])

S_3_46 = np.array([0,
0.830839508,
1.83615576,
2.370796,
2.44836804,
2.43542952,
2.50396232,
2.12634828,
1.94698984,
1.76791836,
1.61504464,
1.49344112,
1.33739396,
1.248276,
1.2302988,
1.1891538,
0.9530026,
```

```
0.93158188,
0.87505076,
0.83904994,
0.451876756,
0.395267988,
0.270155116,
0.257858036,
0.20553932,
0.19252906,
0.146277016,
0.127855872,
0.083360867,
0.15616532,
0.118419952,
0.119146636])

Cyclomet1 = np.array([0,
0.074044289,
0.771156892,
1.79950084,
2.95839724,
4.25165,
5.50280404,
6.71066932,
7.89729112,
8.918126,
9.9548956,
10.9326696,
11.8043528,
12.616112,
13.3665968,
14.1963332,
14.7561584,
15.3894116,
15.9242544,
16.433946,
17.3135628,
18.1242248,
18.7661712,
19.3180628,
19.7101008,
20.2584476,
20.657744,
20.9907864,
21.3790264,
21.8343644,
21.9913484,
22.2428604])

Cyclomet2 = np.array([0,
0.060815264,
0.7662676,
1.80398248,
2.98563312,
4.255026,
5.48610972,
6.6616498,
7.82317104,
8.8344856,
9.8320092,
10.7319664,
11.667878,
12.3643468,
13.0898492,
13.8155204,
14.3604068,
14.8753312,
15.4218212,
15.9071212,
16.8789872,
18.1242248,
18.7661712,
19.3180628,
19.7101008,
20.2584476,
20.657744,
20.9669012,
21.279772,
21.687846,
21.837656,
22.137276])

S_4_24 = np.array([0,
0.87321928,
1.99460832,
2.4824994,
2.65365416,
2.66054964,
2.58957768,
2.4519044,
2.3422266,
2.17685324,
2.0065256,
1.84514436,
1.65422312,
1.54982032,
1.37334836,
1.32580584,
1.12890064,
1.08772188,
```

```
0.97947044,
0.89353436,
0.73142306,
0.633698832,
0.533207128,
0.456741572,
0.393297248,
0.392111428,
0.36884066,
0.308352868,
0.271723268,
0.332792576,
0.225540432,
0.230130948])

S_4_22 = np.array([0,
0.730564712,
1.9421284,
2.44335468,
2.59312248,
2.5889278,
2.49964948,
2.34242916,
2.18838228,
2.00919264,
1.83104112,
1.6320006,
1.45403476,
1.3141924,
1.14997532,
1.07225136,
0.8936272,
0.827312432,
0.737509144,
0.649156692,
0.508726064,
0.420997328,
0.332563008,
0.2624629,
0.218229704,
0.214028272,
0.192523996,
0.144953624,
0.13127576,
0.173118748,
0.093020616,
0.091099672])


S_4_20 = np.array([0,
0.1019341,
0.505356816,
0.95607476,
1.34502372,
1.64844172,
1.85352528,
1.9303968,
1.99050648,
1.94773256,
1.88244072,
1.80254768,
1.6589664,
1.55727284,
1.4126872,
1.36992172,
1.19004,
1.12151564,
1.00682448,
0.9126594,
0.74526888,
0.613290068,
0.497292396,
0.413486572,
0.33908122,
0.30937664,
0.275248656,
0.218374872,
0.19065116,
0.230847504,
0.14024748,
0.125952652])

S_3_56 = np.array([0,
0.825816864,
1.8725828,
2.35403416,
2.60362184,
2.52721452,
2.38219844,
2.23600076,
2.07587708,
1.923687,
1.9595992,
1.68096104,
1.48448628,
1.38182212,
1.2302988,
1.1891538,
0.9530026,
0.93158188,
```

```
0.87505076,
0.785753872,
0.615635544,
0.599527804,
0.593041664,
0.257858036,
0.20553932,
0.19252906,
0.146277016,
0.127855872,
0.083360867,
0.15616532,
0.118419952,
0.119146636])

S_3_61 = np.array([0,
0.086850976,
0.457404112,
0.92175772,
1.36236792,
1.99492904,
1.788014,
1.83499948,
1.80022668,
1.75744432,
1.71713488,
1.65275456,
1.51796776,
1.3882534,
1.4614704,
1.46044916,
1.11826624,
1.11698336,
1.02085176,
0.9052744,
0.75339238,
0.68294792,
0.587115096,
0.53653502,
0.43211534,
0.38269914,
0.256168348,
0.218374872,
0.19065116,
0.230847504,
0.14024748,
0.125952652])
```

In [ ]:

In [56]:
```
#Combined concentrations (methyl concentrations of same species averaged)


Int1_combined = (S_4_24 + S_4_22+ S_3_56 + S_3_46) / 2

Int2_combined = (S_4_20 + S_3_61) / 2


Cyclomet = (Cyclomet1 + Cyclomet2) / 2
```

In [ ]:

In [59]:
```
plt.figure(figsize=(6, 4))
plt.title("Experminetal data zoomed out")
plt.xlabel("Time (h)")
plt.ylabel("Concentration / mmol/L")

#plt.grid(True)
plt.plot(Time, Free_Lig_corr, 'darkblue', label = "Free_Lig")
plt.plot(Time, Cyclomet, 'red', label = "Cyclometalated")
plt.plot(Time, S_3_46, 'darkkhaki', label = "3_46")
plt.plot(Time, S_3_56, 'darkorange', label = "3_56")
plt.plot(Time, S_4_22, 'purple', label = "4_22")
plt.plot(Time, S_4_24, 'darkolivegreen', label = "4_24")
plt.plot(Time, S_4_20, 'lightblue', label = "4_20")
plt.plot(Time, S_3_61, 'limegreen', label = "3_61")

plt.legend(loc='best')
plt.show()
```

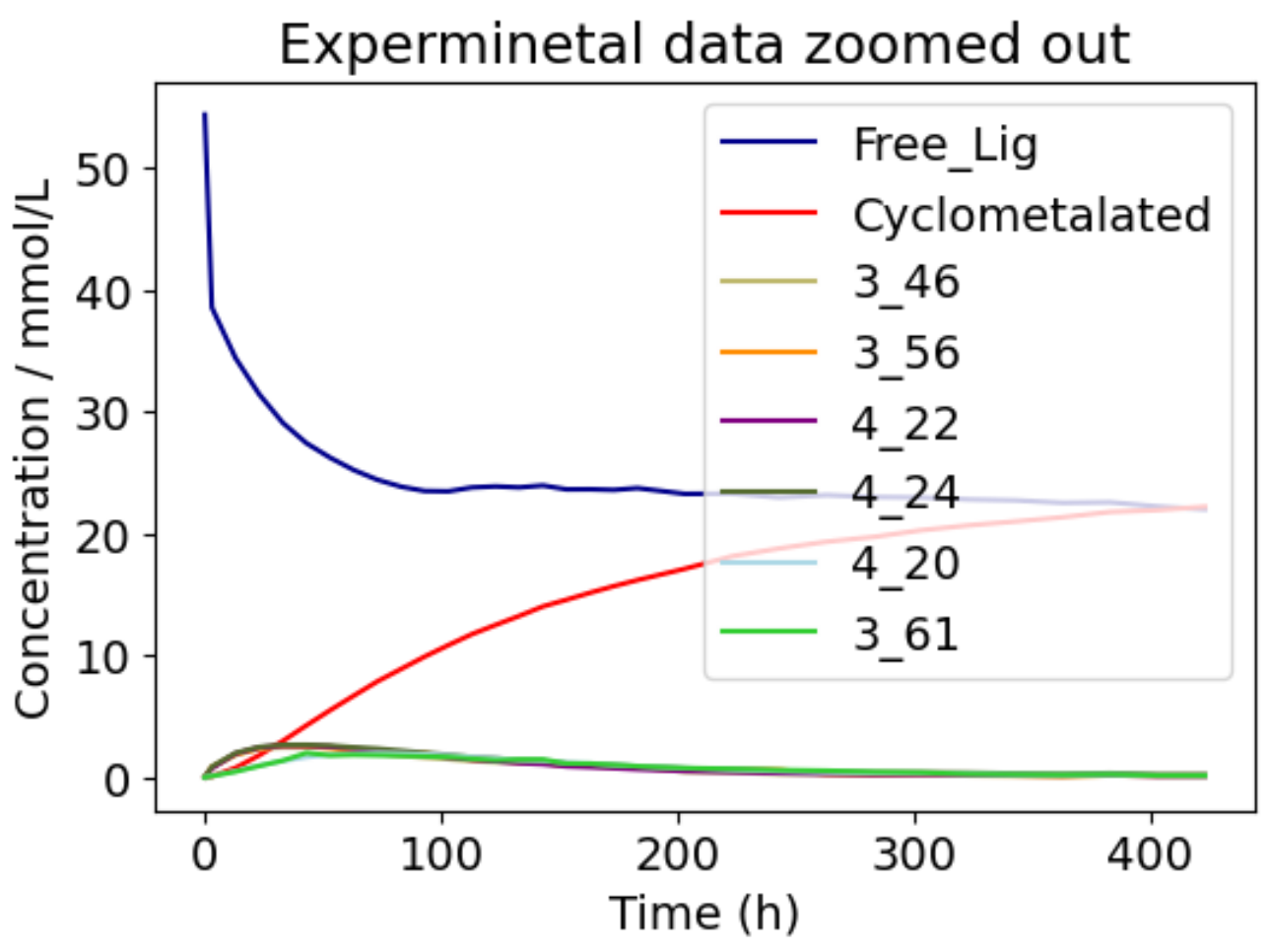

In [61]:
```
fig, ax = plt.subplots(figsize=(8, 5))
plt.title("Experminetal data with overlap correction")
plt.xlabel("Time / min")
plt.ylabel("Concentration / mmol/L")

#plt.grid(True)
plt.plot(Time, Free_Lig_corr, 'darkblue', label = "Free Lig")
plt.plot(Time, Cyclomet, 'red', label = "Cyclometalated")
plt.plot(Time, S_3_46, 'darkkhaki', label = "3_46")
plt.plot(Time, S_3_56, 'darkorange', label = "3_56")
plt.plot(Time, S_4_22, 'purple', label = "4_22")
plt.plot(Time, S_4_24, 'darkolivegreen', label = "4_24")
plt.plot(Time, S_4_20, 'lightblue', label = "4_20")
plt.plot(Time, S_3_61, 'limegreen', label = "3_61")

ax.set_ylim([0, 3])

plt.legend(loc='best')
plt.show()
```

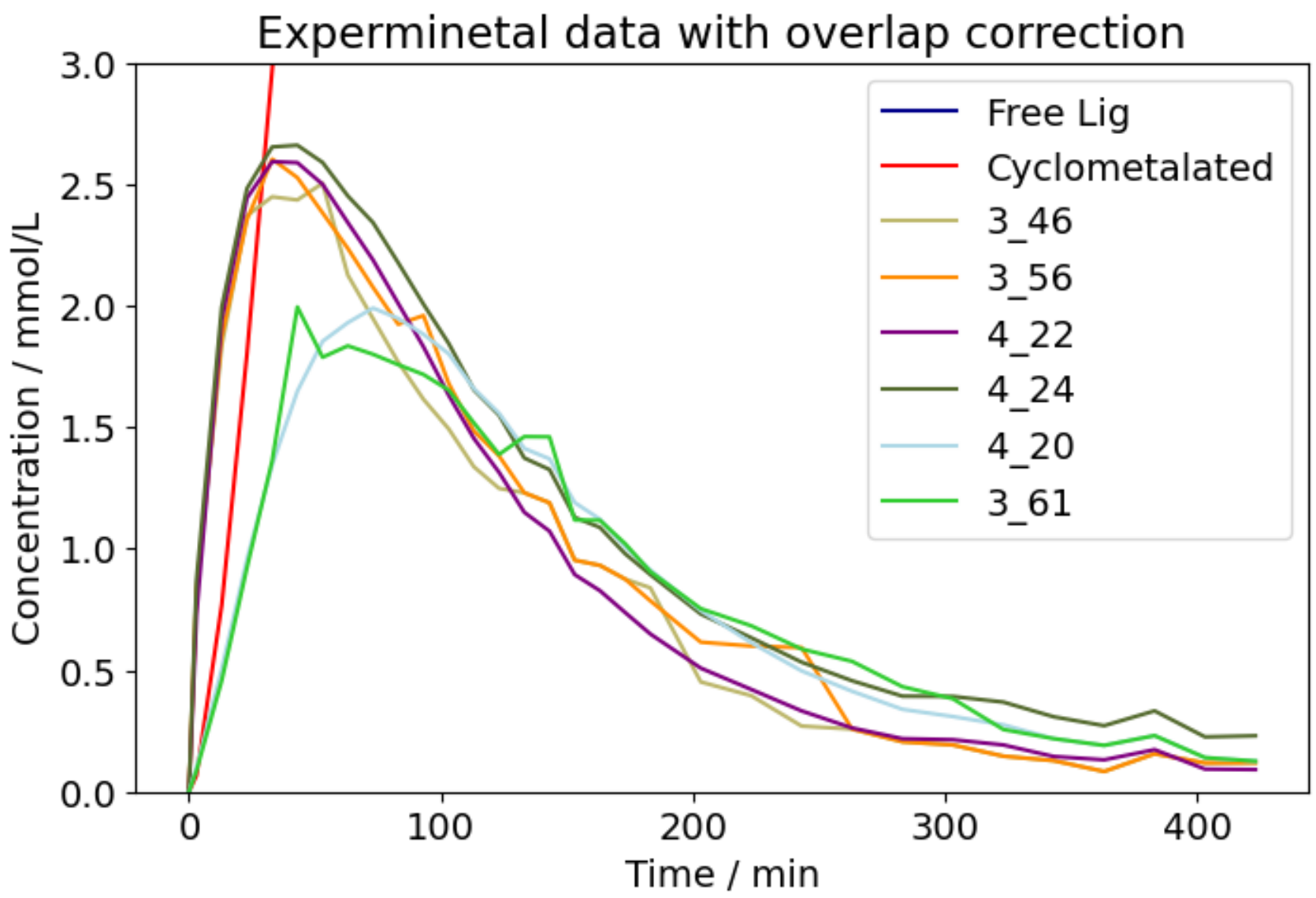

In [63]:
```
fig, ax = plt.subplots(figsize=(8, 5))
plt.title("Experminetal data with overlap correction + combined species")
plt.xlabel("Time / min")
plt.ylabel("Concentration / mmol/L")

#plt.grid(True)
plt.plot(Time, Free_Lig_corr, 'darkblue', label = "Free Lig")
plt.plot(Time, Cyclomet, 'red', label = "Cyclometalated")
plt.plot(Time, Int1_combined, 'darkorange', label = "Int1")
plt.plot(Time, Int2_combined, 'darkolivegreen', label = "Int2")

ax.set_ylim([0, 60])


plt.legend(loc='best')
plt.show()
```

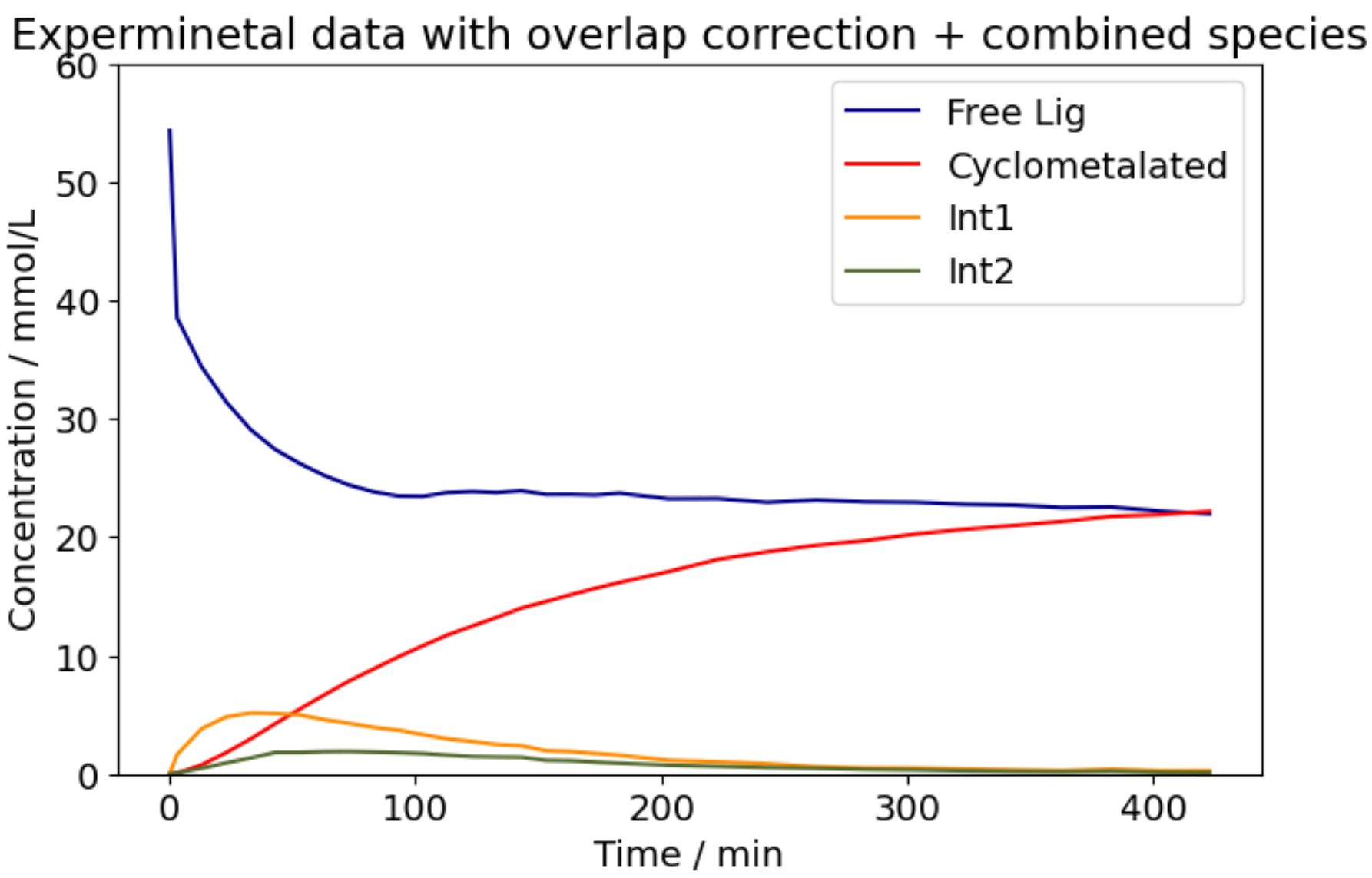

Free Lig (A) -> Int1 (B) -> Int2 (C) -> cyclomet (D)

In [ ]:

In [67]:
```
#same as before but without Free Lig with no A + another A side reaction
import numpy as np
import matplotlib.pyplot as plt
from scipy.integrate import odeint
import ipywidgets as widgets
from ipywidgets import interactive

# Define the system of ODEs
def model(y, t, k1, k2, k3, k4, A0):
    A, B, C, D = y      ### Change here

    # Rate equations
    dA_dt = -k1 * A -k4 * A
    dB_dt = k1 * A -k2 * B
    dC_dt = k2 * B -k3 * C
    dD_dt = k3 * C

    return [dA_dt, dB_dt, dC_dt, dD_dt] ### Change here

# Initial conditions: [A0, B0, C0, D0]
A0 = 64.4 * 0.844 # Initial concentration of A = 64.4 mmol/g (max) - all species recorded
B0 = 0   # Initial concentration of B
C0 = 0  # Initial concentration of C
D0 = 0   # Initial concentration of D
#E0 = 0.0     ### Change here

# Time points where solution is computed
t = np.linspace(0, 420, 500)  # from t=0 to t=50, with 500 time points

# Initial values vector
y0 = [A0, B0, C0, D0]     ### Change here
```

Optimization of the variables

In [70]:
```
from scipy.optimize import minimize

def objective(params, t_exp, data):
    k1, k2, k3, k4 = params

    # Solve ODE system with current parameters
    solution = odeint(model, y0, t_exp, args=(k1, k2, k3, k4, A0))

    #A_t = solution[:, 0]
    B_t = solution[:, 1]
    C_t = solution[:, 2]
    D_t = solution[:, 3]

    # Experimental data
    A_exp = Free_Lig_corr
    B_exp = Int1_combined
    C_exp = Int2_combined
    D_exp = Cyclomet

    # Compute sum of squared errors (SSE)
    sse = np.sum((C_exp - C_t)**2 + (D_exp - D_t)**2 + (B_exp - B_t)**2)
    # sse = np.sum((A_exp - A_t)**2 + (C_exp - C_t)**2 + (D_exp - D_t)**2)

    return sse
```

In [72]:
```
# Experimental time points
t_exp = Time

# Initial guess for k1, k2, k3, k4
```

```
initial_guess = [0.01, 0.002, 0.002, 0.002]
df_data = pd.DataFrame ({"Time (h)":Time, "A_exp": Free_Lig_corr, "B_exp": Int1_combined, "C_exp": Int2_combined, "D_exp": Cyclomet})


# Run optimization
result = minimize(objective, initial_guess, args=(t_exp, df_data), method='Nelder-Mead')


SSE_min = result.fun
best_k1, best_k2, best_k3, best_k4 = result.x
print("Optimized k1 =", best_k1)
print("Optimized k2 =", best_k2)
print("Optimized k3 =", best_k3)
print("Optimized k4 =", best_k4)
print("SSE_min =", SSE_min)
```

```
Optimized k1 = 0.004922103839896792
Optimized k2 = 0.03053111441591949
Optimized k3 = 0.08255820640676707
Optimized k4 = 0.0076187384093521085
SSE_min = 26.281661987946595
```

In [74]:

```
# Confidence intervals for the optimized parameters (k1-k4)
# Nelder-Mead doesn't return a Hessian/covariance, so it's estimated numerically

from scipy import stats

best_params = result.x  # [best_k1, best_k2, best_k3, best_k4]

# Degrees of freedom: 3 fitted series (B, C, D) x len(Time) points, minus 4 parameters
n_points = 3 * len(Time)
p_params = len(best_params)
dof = n_points - p_params

# Residual variance estimate (reduced chi-square)
sigma2 = result.fun / dof

def sse_func(params):
    return objective(params, t_exp, df_data)

def numerical_hessian(f, x0, rel_step=1e-4):
    n = len(x0)
    H = np.zeros((n, n))
    steps = np.maximum(np.abs(x0) * rel_step, 1e-8)
    for i in range(n):
        for j in range(n):
            xpp = x0.copy(); xpp[i] += steps[i]; xpp[j] += steps[j]
            xpm = x0.copy(); xpm[i] += steps[i]; xpm[j] -= steps[j]
            xmp = x0.copy(); xmp[i] -= steps[i]; xmp[j] += steps[j]
            xmm = x0.copy(); xmm[i] -= steps[i]; xmm[j] -= steps[j]
            H[i, j] = (f(xpp) - f(xpm) - f(xmp) + f(xmm)) / (4 * steps[i] * steps[j])
    return H

# Hessian of SSE at the optimum
H = numerical_hessian(sse_func, best_params)

# For SSE = sum(residuals^2): Hessian(SSE) ~= 2 * J^T J  (Gauss-Newton approx)
JTJ = H / 2.0
JTJ_inv = np.linalg.inv(JTJ)

# Parameter covariance matrix
cov = sigma2 * JTJ_inv
se = np.sqrt(np.diag(cov))

alpha = 0.05
t_crit = stats.t.ppf(1 - alpha / 2, dof)

names = ["k1", "k2", "k3", "k4"]
print(f"dof = {dof}, sigma^2 = {sigma2:.6g}, t_crit (95%) = {t_crit:.4f}\n")
print("95% Confidence Intervals:")
for name, val, s in zip(names, best_params, se):
    lo, hi = val - t_crit * s, val + t_crit * s
    print(f"{name} = {val:.6f}  ±  {t_crit*s:.6f}   (95% CI: [{lo:.6f}, {hi:.6f}])")

print("\nCovariance matrix:")
print(cov)
```

```
dof = 92, sigma^2 = 0.28567, t_crit (95%) = 1.9861

95% Confidence Intervals:
k1 = 0.004922  ±  0.000340   (95% CI: [0.004582, 0.005262])
k2 = 0.030531  ±  0.002285   (95% CI: [0.028246, 0.032817])
k3 = 0.082558  ±  0.015958   (95% CI: [0.066600, 0.098517])
k4 = 0.007619  ±  0.000729   (95% CI: [0.006890, 0.008348])

Covariance matrix:
[[ 2.93187623e-08 -9.04561691e-08 -5.57141182e-07  6.01689867e-08]
 [-9.04561691e-08  1.32421277e-06 -1.45465458e-06 -1.72755335e-07]
 [-5.57141182e-07 -1.45465458e-06  6.45631055e-05 -1.00611726e-06]
 [ 6.01689867e-08 -1.72755335e-07 -1.00611726e-06  1.34750746e-07]]
```

Identifiability analysis of k1-k4

In [37]:

```
# Identifiability analysis of the fitted parameters (k1-k4)
# Two checks:
#  1) Parameter correlation matrix (from the covariance matrix above) -> pairwise identifiability
#  2) Profile likelihood for each parameter

# 1) Correlation matrix
D_ = np.diag(1 / se)
```

```python
corr = D_ @ cov @ D_
corr_df = pd.DataFrame(corr, index=names, columns=names)
print("Correlation matrix:")
print(corr_df.round(3))
print("\n|corr| > 0.9 flags strong pairwise correlation (practical non-identifiability):")
strong_pairs = []
for i in range(len(names)):
    for j in range(i + 1, len(names)):
        if abs(corr[i, j]) > 0.9:
            strong_pairs.append((names[i], names[j], corr[i, j]))
            print(f"  -> {names[i]} & {names[j]}: corr = {corr[i, j]:.3f}  (STRONGLY CORRELATED)")
if not strong_pairs:
    print("  (none)")


#  2) Profile likelihood
alpha = 0.05
delta_chi2 = stats.chi2.ppf(1 - alpha, df=1)      # 1 parameter of interest at a time
threshold = SSE_min * (1 + delta_chi2 / dof)
print(f"\nProfile-likelihood SSE threshold (95%, 1 dof): {threshold:.4f}  (SSE_min = {SSE_min:.4f})")

def profile_param(idx, span=0.6, npts=13):
    val0 = best_params[idx]
    grid = val0 * np.linspace(1 - span, 1 + span, npts)
    grid = grid[grid > 0]
    sses = []
    for v in grid:
        def obj_fixed(p_other):
            p = np.insert(p_other, idx, v)
            return objective(p, t_exp, df_data)
        p0_other = np.delete(best_params, idx)
        r = minimize(obj_fixed, p0_other, method='Nelder-Mead',
                     options={'xatol': 1e-10, 'fatol': 1e-10, 'maxiter': 2000})
        sses.append(r.fun)
    return grid, np.array(sses)

def lin_interp(x0, y0, x1, y1, ytarget):
    if y1 == y0:
        return x0
    return x0 + (ytarget - y0) * (x1 - x0) / (y1 - y0)

def find_crossings(grid, sses, val0, threshold):
    idx0 = np.argmin(np.abs(grid - val0))
    left_grid, left_sse = grid[:idx0 + 1], sses[:idx0 + 1]
    right_grid, right_sse = grid[idx0:], sses[idx0:]
    lo = hi = None
    for i in range(len(left_grid) - 1, 0, -1):
        if (left_sse[i] - threshold) * (left_sse[i - 1] - threshold) <= 0 and left_sse[i - 1] != left_sse[i]:
            lo = lin_interp(left_grid[i], left_sse[i], left_grid[i - 1], left_sse[i - 1], threshold)
            break
    for i in range(len(right_grid) - 1):
        if (right_sse[i] - threshold) * (right_sse[i + 1] - threshold) <= 0 and right_sse[i + 1] != right_sse[i]:
            hi = lin_interp(right_grid[i], right_sse[i], right_grid[i + 1], right_sse[i + 1], threshold)
            break
    return lo, hi

profiles = {}
print("\nRunning profile likelihood scans (re-optimizing 3 parameters at ~13 grid points, 4x)...")
for idx, name in enumerate(names):
    grid, sses = profile_param(idx)
    profiles[name] = (grid, sses)

print("\nProfile-likelihood 95% CIs (vs. linearized CI from the previous cell):")
for idx, (name, val0, s) in enumerate(zip(names, best_params, se)):
    grid, sses = profiles[name]
    lo, hi = find_crossings(grid, sses, val0, threshold)
    lo_s = f"{lo:.5f}" if lo is not None else "< scanned range (flat/unbounded)"
    hi_s = f"{hi:.5f}" if hi is not None else "> scanned range (flat/unbounded)"
    lin_lo, lin_hi = val0 - t_crit * s, val0 + t_crit * s
    print(f"{name}: best={val0:.5f}")
    print(f"    profile CI    ~= [{lo_s}, {hi_s}]")
    print(f"    linearized CI ~= [{lin_lo:.5f}, {lin_hi:.5f}]")

# ---------- Plot the profiles ----------
fig, axes = plt.subplots(1, 4, figsize=(18, 4))
for ax, name in zip(axes, names):
    grid, sses = profiles[name]
    ax.plot(grid, sses, 'o-', color='steelblue')
    ax.axhline(threshold, color='red', linestyle='--', label='95% threshold')
    ax.axvline(best_params[names.index(name)], color='gray', linestyle=':')
    ax.set_title(name)
    ax.set_xlabel(name)
    ax.set_ylabel("SSE")
    ax.legend(fontsize=8)
plt.tight_layout()
plt.show()
```

```
Correlation matrix:
       k1     k2     k3     k4
k1  1.000 -0.459 -0.405  0.957
k2 -0.459  1.000 -0.157 -0.409
k3 -0.405 -0.157  1.000 -0.341
k4  0.957 -0.409 -0.341  1.000

|corr| > 0.9 flags strong pairwise correlation (practical non-identifiability):
  -> k1 & k4: corr = 0.957  (STRONGLY CORRELATED)

Profile-likelihood SSE threshold (95%, 1 dof): 27.3791  (SSE_min = 26.2817)

Running profile likelihood scans (re-optimizing 3 parameters at ~13 grid points, 4x)...

Profile-likelihood 95% CIs (vs. linearized CI from the previous cell):
k1: best=0.00492
    profile CI    ~= [0.00472, 0.00518]
    linearized CI ~= [0.00458, 0.00526]
k2: best=0.03053
    profile CI    ~= [0.02916, 0.03252]
    linearized CI ~= [0.02825, 0.03282]
k3: best=0.08256
    profile CI    ~= [0.07031, 0.10178]
    linearized CI ~= [0.06660, 0.09852]
k4: best=0.00762
    profile CI    ~= [0.00700, 0.00836]
    linearized CI ~= [0.00689, 0.00835]
```

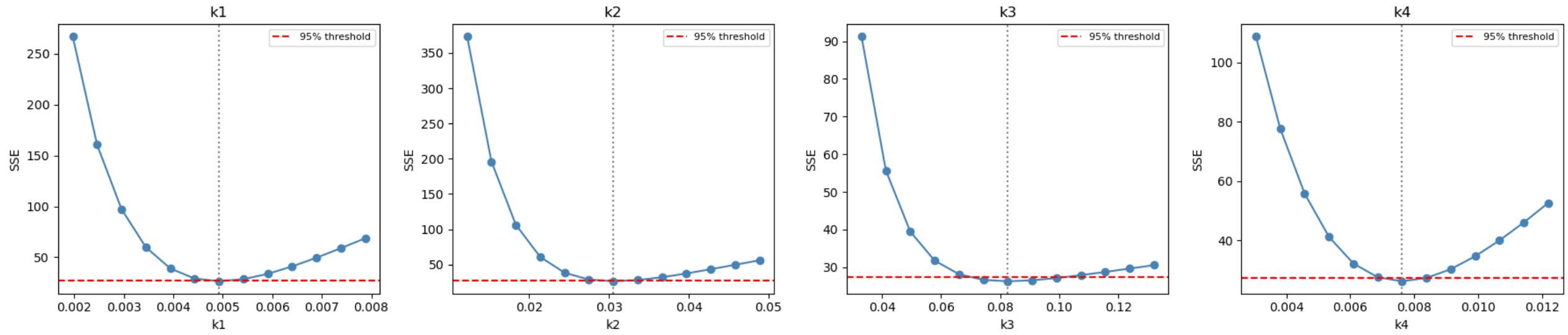

In [38]:
```
#Residual analysis (r squared for indivivual concentrations and combined)
solution_opt = odeint(model, y0, t_exp, args=(best_k1, best_k2, best_k3, best_k4, A0))

A_fit = solution_opt[:, 0]
B_fit = solution_opt[:, 1]
C_fit = solution_opt[:, 2]
D_fit = solution_opt[:, 3]

res_B = Int1_combined - B_fit #intermediate A residuals
res_C = Int2_combined - C_fit #intermediate B residuals
res_D = Cyclomet - D_fit #cyclometalated product residuals


rmse_B = np.sqrt(np.mean(res_B**2)) #root mean square errors
rmse_C = np.sqrt(np.mean(res_C**2))
rmse_D = np.sqrt(np.mean(res_D**2))
print(rmse_B, rmse_C,rmse_D)

#individial R squared calculation
ss_res_B = np.sum(res_B**2)
ss_tot_B = np.sum((Int1_combined - np.mean(Int1_combined))**2)
r2_B = 1 - ss_res_B / ss_tot_B
print("R squared Int 1 = ",r2_B)

ss_res_C = np.sum(res_C**2)
ss_tot_C = np.sum((Int2_combined - np.mean(Int2_combined))**2)
r2_C = 1 - ss_res_C / ss_tot_C
print("R squared Int 2 = ",r2_C)

ss_res_D = np.sum(res_D**2)
ss_tot_D = np.sum((Cyclomet - np.mean(Cyclomet))**2)
r2_D = 1 - ss_res_D / ss_tot_D
print("R squared Cyclomet = ",r2_D)

#R squared combined
ss_res_comb = ss_res_B + ss_res_C + ss_res_D
ss_tot_comb = ss_tot_B + ss_tot_C + ss_tot_D
r2_comb = 1 - ss_res_comb / ss_tot_comb
print("R squared combined = ",r2_comb)
```

```
0.37740212065839396 0.2391293614793921 0.7884711313195534
R squared Int 1 =  0.9500268393660646
R squared Int 2 =  0.8622826605674122
R squared Cyclomet =  0.9876863443432765
R squared combined =  0.9847208111153875
```

In [39]:
```
# Solve ODE with optimized parameters
solution_opt = odeint(model, y0, t_exp, args=(best_k1, best_k2, best_k3, best_k4, A0))


#ax.tick_params(axis='both', which='major', labelsize=14)
#ax.tick_params(axis='both', which='minor', labelsize=8)

A_fit = solution_opt[:, 0]
B_fit = solution_opt[:, 1]
C_fit = solution_opt[:, 2]
D_fit = solution_opt[:, 3]

# Plot results
```

```
fig, ax = plt.subplots(figsize=(10, 6))
plt.plot(Time, df_data["A_exp"], 'bo', label=" Free Ligand")
plt.plot(Time, df_data["B_exp"], 'yo', label="Intermed. 1")
plt.plot(Time, df_data["C_exp"], 'ro', label="Intermed. 2")
plt.plot(Time, df_data["D_exp"], 'go', label="Cyclomet.")

#plt.plot(t_exp, A_fit, 'b-', label="A_fit")
plt.plot(t_exp, B_fit, 'y-', label="Intermed. 1 fit")
plt.plot(t_exp, C_fit, 'r-', label="Intermed. 2 fit")
plt.plot(t_exp, D_fit, 'g-', label="Cyclomet. fit")

ax.set_ylim([0, 60])
ax.set_xlim([0, 450])

plt.xlabel("time / min")
plt.ylabel("concentration / mmol/L")
plt.legend()
plt.savefig('PTbisPYA_MeCN_fit_4.png')
```

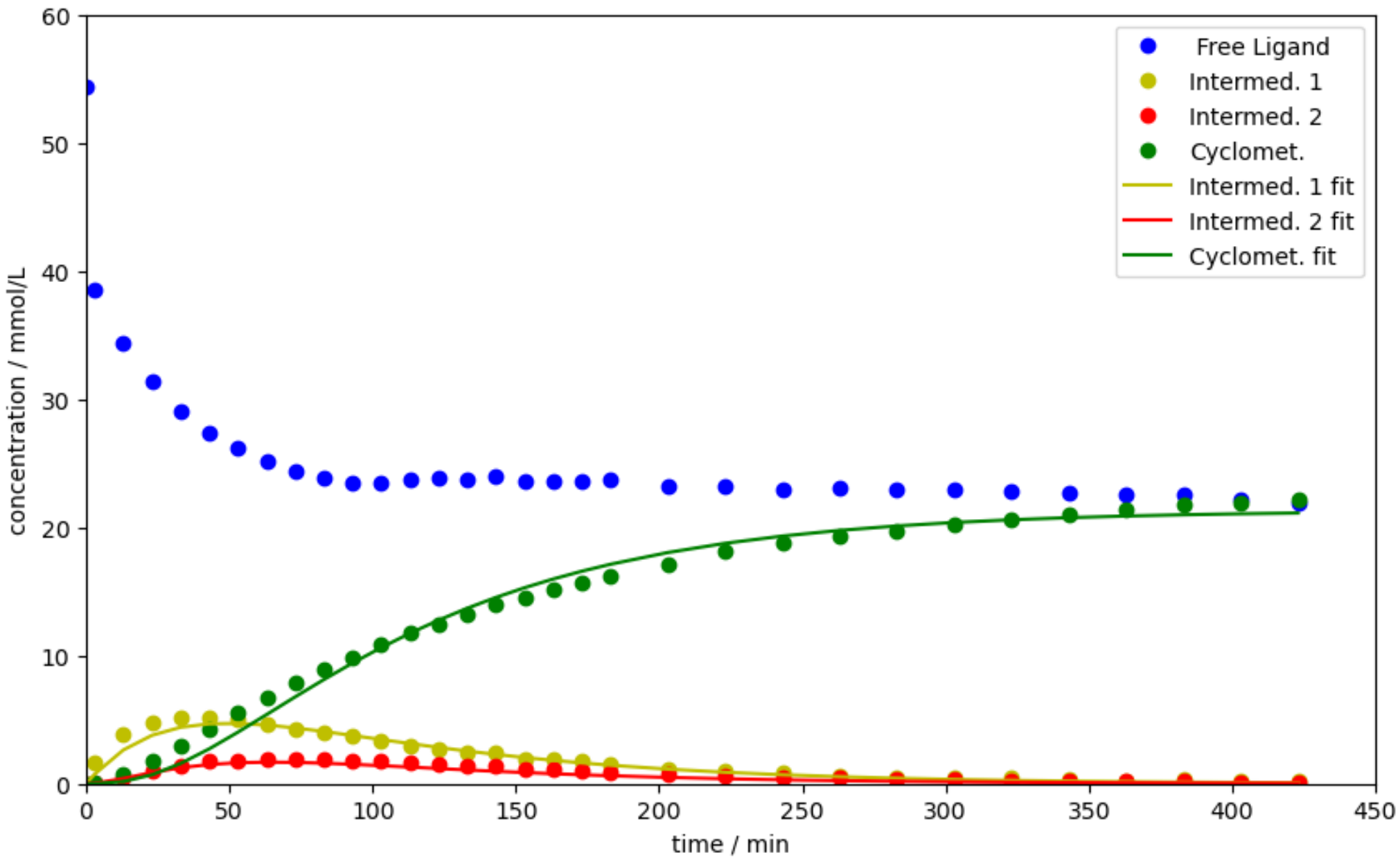

In [ ]:

In [41]:
```
# Solve ODE with optimized parameters
solution_opt = odeint(model, y0, t_exp, args=(best_k1, best_k2, best_k3, best_k4, A0))

A_fit = solution_opt[:, 0]
B_fit = solution_opt[:, 1]
C_fit = solution_opt[:, 2]
D_fit = solution_opt[:, 3]


# Plot results
plt.rcParams.update({'font.size': 14})
#ax.tick_params(axis='both', which='major', labelsize=14)
#ax.tick_params(axis='both', which='minor', labelsize=8)
csfont = {'fontname':'Arial'}
hfont = {'fontname':'Arial'}



#fig, ax = plt.subplots(figsize=(6, 4))
fig, ax = plt.subplots(figsize=(10, 5))
#plt.plot(Time, df_data["A_exp"], 'bo', label=" Free Ligand")
plt.plot(Time, df_data["B_exp"], 'yo', label="Intermediate A")
plt.plot(Time, df_data["C_exp"], 'ro', label="Intermediate B")
plt.plot(Time, df_data["D_exp"], 'go', label="Complex 2")

#plt.plot(t_exp, A_fit, 'b-', label="A_fit")
plt.plot(t_exp, B_fit, 'y-')
plt.plot(t_exp, C_fit, 'r-')
plt.plot(t_exp, D_fit, 'g-')

ax.set_ylim([0, 35])
ax.set_xlim([0, 430])

plt.xlabel("Time [min]",  **hfont)
plt.ylabel("Concentration  [mmol/L]",  **hfont)
#plt.legend()
plt.savefig('PTbisPYA_MeCN_fit_3.png')
```

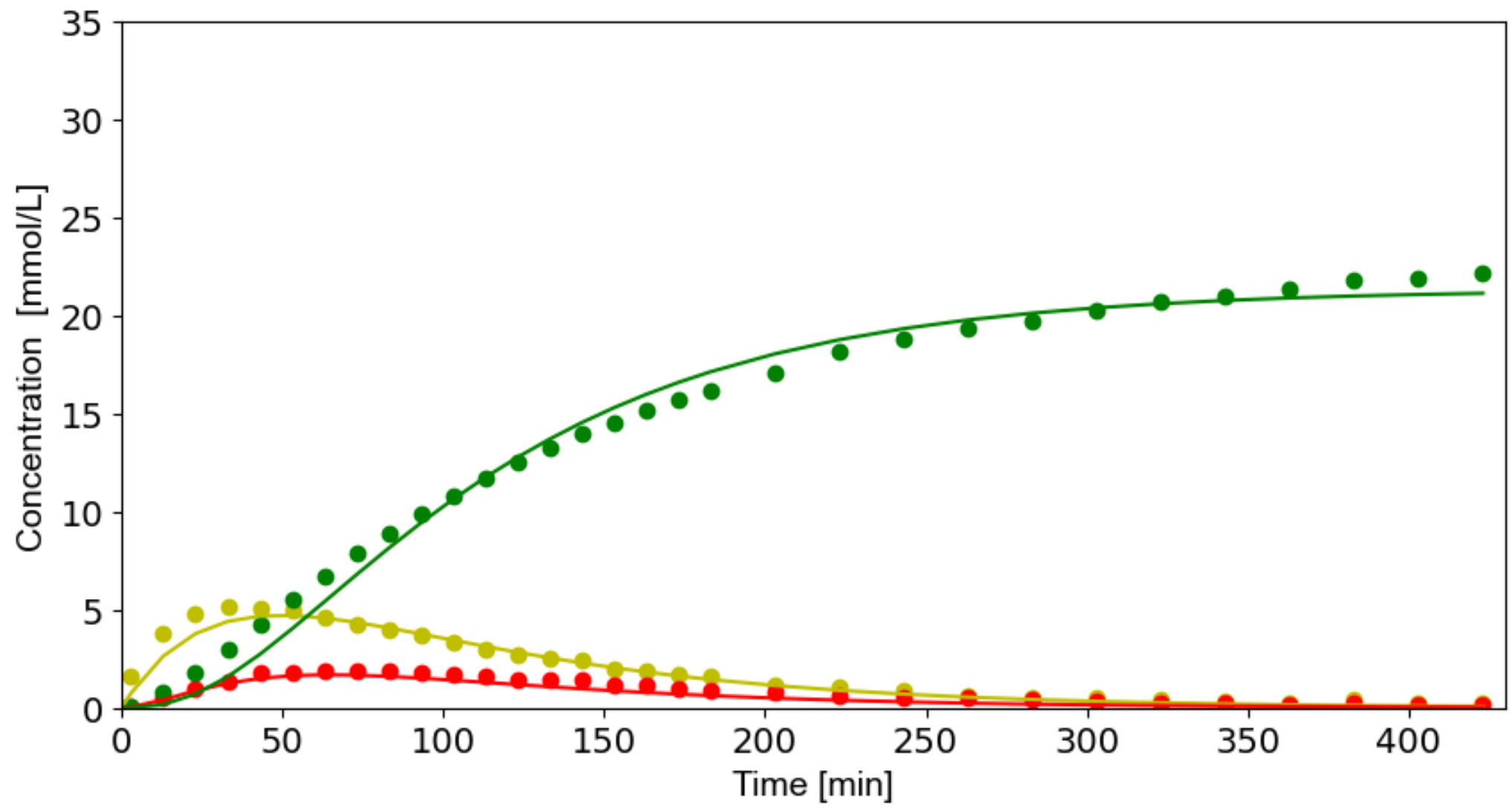
35
30
25
20
15
10
5
0
Concentration [mmol/L]
0
50
100
150
200
250
300
350
400
Time [min]

Kinetic Model Complex 2 Formation 75 deg (Int. 1 Combined)

In [35]:
```
import numpy as np
import pandas as pd
import matplotlib.pyplot as plt
import math
```

NMR Data

In [38]:
```
#Data arrays, c in mmol/L, first concentration values for t = 0 (in dead time) was fixed at added starting conditions

Time = np.array([0,
3,
6,
9,
12,
15,
18,
21,
24,
27,
30,
33,
36,
39,
42,
45,
48,
51,
54,
57,
60,
63,
68,
73,
78,
83,
88,
93,
98,
103,
108,
113,
118,
123])


Free_Lig = np.array([42.5,
27.03264201,
25.27791983,
23.82243319,
22.62724681,
21.61839678,
20.70383426,
19.97688107,
19.39223704,
18.90188053,
18.53135871,
18.08906776,
17.77517111,
17.45666544,
17.27836197,
17.09795151,
16.86038912,
16.88426826,
16.74924574,
16.54416601,
16.35089417,
16.22825018,
16.07882993,
15.95030394,
15.78257927,
15.58259143,
15.54277822,
15.3842716,
15.63921661,
15.48150011,
15.14332926,
14.97766769,
14.84786873,
14.85159984])

S_3_47 = np.array([0,
0.825445897,
1.004925773,
1.079082813,
1.086378243,
1.06256933,
1.013090334,
0.948010882,
0.860550875,
0.806698133,
0.770329843,
0.726048073,
0.699984687,
0.57576572,
```

```
0.519938559,
0.471918649,
0.434723368,
0.39149509,
0.363834786,
0.320126292,
0.2872275,
0.364422986,
0.32975784,
0.306591556,
0.207845142,
0.193154199,
0.352245499,
0.32119822,
0.308004991,
0.277686377,
0.104180676,
0.101778715,
0.072472243,
0.116416105])


Cyclomet1 = np.array([0,
0.511858277,
1.126050986,
1.847833296,
2.603099084,
3.345512274,
4.086942204,
4.758279817,
5.419811178,
6.008230211,
6.54813595,
7.048184585,
7.498332828,
7.923838142,
8.265555747,
8.61352407,
8.913330262,
9.18407764,
9.439461596,
9.639010491,
9.826532021,
10.02959255,
10.19033784,
10.33870459,
10.53316161,
10.70918252,
10.97782291,
11.10608553,
11.50913392,
11.62738836,
11.55724337,
11.63327036,
11.71078979,
11.84221289])

Cyclomet2 = np.array([0,
0.512653663,
1.124321504,
1.844075842,
2.583223207,
3.328349137,
4.059156359,
4.738017659,
5.386775432,
5.979584015,
6.531701479,
7.029485107,
7.471591696,
7.897167243,
8.246074929,
8.580839489,
8.887607505,
9.170118874,
9.433052855,
9.655251822,
9.826532021,
10.02959255,
10.19033784,
10.33870459,
10.53316161,
10.70918252,
10.97782291,
11.10608553,
11.50913392,
11.62738836,
11.55724337,
11.63327036,
11.71289678,
11.86468738])

S_3_57 = np.array([0,
1.169068566,
1.653244592,
1.610218233,
1.667317486,
1.665201723,
1.626968752,
1.555787826,
```

```
1.469375166,
1.555471779,
1.305223595,
1.235517558,
1.189997935,
1.051288189,
0.950627053,
0.900954917,
0.83859084,
0.772663327,
0.731989766,
0.689067001,
0.652092075,
0.622679463,
0.579415191,
0.560121368,
0.562794603,
0.591856928,
0.610613471,
0.424085735,
0.392713629,
0.399415592,
0.283681623,
0.266106747,
0.267698397,
0.241715779])



S_3_63 = np.array([0,
0.264177101,
0.50740903,
0.973830209,
0.896433682,
0.984654836,
1.033378829,
1.04096397,
1.025258164,
0.990378808,
0.952769153,
0.901472884,
0.794888491,
0.738616229,
0.677794639,
0.629041675,
0.585130384,
0.531843016,
0.500105699,
0.456882689,
0.405577641,
0.387245128,
0.360502241,
0.332675134,
0.306795232,
0.280731846,
0.341135551,
0.313657852,
0.290145672,
0.304029816,
0.180936329,
0.162014739,
0.157101956,
0.138480612])

S_4_21 = np.array([0,
0.309421938,
0.557830901,
0.774086418,
0.922358358,
1.009745498,
1.039673443,
1.0344411,
1.010412709,
0.973285905,
0.913157863,
0.852890235,
0.794888491,
0.738616229,
0.677794639,
0.629041675,
0.585130384,
0.531843016,
0.500105699,
0.456882689,
0.405577641,
0.387245128,
0.360502241,
0.332675134,
0.306795232,
0.280731846,
0.341135551,
0.313657852,
0.290145672,
0.304029816,
0.180936329,
0.162014739,
0.157101956,
0.138480612])
```

```
S_4_23 = np.array([0,
0.825445897,
1.004925773,
1.079082813,
1.086378243,
1.06256933,
1.033545632,
0.958624811,
0.884063933,
0.825854125,
0.745688671,
0.682594172,
0.639877716,
0.57576572,
0.519938559,
0.471918649,
0.434723368,
0.39149509,
0.363834786,
0.320126292,
0.2872275,
0.287733176,
0.259956989,
0.239782622,
0.240620148,
0.219651273,
0.283071475,
0.276279087,
0.272344296,
0.290630279,
0.152688704,
0.137967912,
0.141909727,
0.118033215])


S_4_26 = np.array([0,
1.034915172,
1.371066828,
1.56061633,
1.607558167,
1.596496503,
1.577419524,
1.521654694,
1.424838803,
1.337126836,
1.240188038,
1.155285382,
1.080311887,
1.018436805,
0.916160315,
0.863127541,
0.79881187,
0.737010532,
0.688858059,
0.63660838,
0.584412254,
0.557494662,
0.5134868,
0.479844419,
0.455270846,
0.412998612,
0.454677379,
0.424085735,
0.392713629,
0.399415592,
0.283681623,
0.266106747,
0.267698397,
0.241715779])
```

In [ ]:

In [41]:

```
#Combined concentrations ( with similar species) in mmol/L

Cyclomet = (Cyclomet1 + Cyclomet2) / 2
Int1_combined = (S_4_26 + S_4_23 + S_3_57 + S_3_47) / 2
Int2_combined = (S_4_21 + S_3_63) / 2
Free_Lig_corr = Free_Lig

#Combined concentrations ( with similar species, only same conc)
```

Free Lig (A) -> Int1 (B) -> Int2 (C) -> cyclomet (D)

In [ ]:

In [45]:

```
#same as before but without Free Lig with no A + another A side reaction
import numpy as np
import matplotlib.pyplot as plt
from scipy.integrate import odeint
import ipywidgets as widgets
from ipywidgets import interactive

# Define the system of ODEs
def model(y, t, k1, k2, k3, k4, A0):
    A, B, C, D = y      ### Change here
```

```
    # Rate equations
    dA_dt = -k1 * A -k4 * A
    dB_dt = k1 * A -k2 * B
    dC_dt = k2 * B -k3 * C
    dD_dt = k3 * C

    return [dA_dt, dB_dt, dC_dt, dD_dt] ### Change here


# Initial conditions: [A0, B0, C0, D0]
A0 = 42.5  # Initial concentration of A = 42.5 mmol/L (max) - all species recorded
B0 = 0   # Initial concentration of B
C0 = 0  # Initial concentration of C
D0 = 0   # Initial concentration of D
E0 = 0    ### Change here

# Time points where solution is computed
t = np.linspace(0, 120, 500)  # from t=0 to t=50, with 500 time points

# Initial values vector
y0 = [A0, B0, C0, D0]    ### Change here
```

Optimization of the variables

In [48]:
```
from scipy.optimize import minimize

def objective(params, t_exp, data):
    k1, k2, k3, k4 = params

    # Solve ODE system with current parameters
    solution = odeint(model, y0, t_exp, args=(k1, k2, k3, k4, A0))

    #A_t = solution[:, 0]
    B_t = solution[:, 1]
    C_t = solution[:, 2]
    D_t = solution[:, 3]

    # Experimental data
    A_exp = Free_Lig_corr
    B_exp = Int1_combined
    C_exp = Int2_combined
    D_exp = Cyclomet

    # Compute sum of squared errors (SSE)
    sse = np.sum((C_exp - C_t)**2 + (D_exp - D_t)**2 + (B_exp - B_t)**2)
    # sse = np.sum((A_exp - A_t)**2 + (C_exp - C_t)**2 + (D_exp - D_t)**2)

    return sse
```

In [54]:
```
# Experimental time points
t_exp = Time

# Initial guess for k1, k2, k3, k4
initial_guess = [0.01, 0.1, 0.2, 0.01]
df_data = pd.DataFrame ({"Time (h)":Time, "A_exp": Free_Lig_corr, "B_exp": Int1_combined, "C_exp": Int2_combined, "D_exp": Cyclomet})


# Run optimization
result = minimize(objective, initial_guess, args=(t_exp, df_data), method='Nelder-Mead')


SSE_min = result.fun
best_k1, best_k2, best_k3, best_k4 = result.x
print("Optimized k1 =", best_k1)
print("Optimized k2 =", best_k2)
print("Optimized k3 =", best_k3)
print("Optimized k4 =", best_k4)
print("SSE_min =", SSE_min)
```

```
Optimized k1 = 0.013443331344868924
Optimized k2 = 0.09980351141294905
Optimized k3 = 0.36779236044572017
Optimized k4 = 0.03635399518006792
SSE_min = 13.686178478798503
```

In [56]:
```
# Confidence intervals for the optimized parameters (k1-k4)
# Nelder-Mead doesn't return a Hessian/covariance, so it's estimated numerically

from scipy import stats

best_params = result.x  # [best_k1, best_k2, best_k3, best_k4]

# Degrees of freedom: 3 fitted series (B, C, D) x len(Time) points, minus 4 parameters
n_points = 3 * len(Time)
p_params = len(best_params)
dof = n_points - p_params

# Residual variance estimate (reduced chi-square)
sigma2 = result.fun / dof

def sse_func(params):
    return objective(params, t_exp, df_data)

def numerical_hessian(f, x0, rel_step=1e-4):
    n = len(x0)
    H = np.zeros((n, n))
    steps = np.maximum(np.abs(x0) * rel_step, 1e-8)
    for i in range(n):
        for j in range(n):
            xpp = x0.copy(); xpp[i] += steps[i]; xpp[j] += steps[j]
```

```
            xpm = x0.copy(); xpm[i] += steps[i]; xpm[j] -= steps[j]
            xmp = x0.copy(); xmp[i] -= steps[i]; xmp[j] += steps[j]
            xmm = x0.copy(); xmm[i] -= steps[i]; xmm[j] -= steps[j]
            H[i, j] = (f(xpp) - f(xpm) - f(xmp) + f(xmm)) / (4 * steps[i] * steps[j])
    return H

# Hessian of SSE at the optimum
H = numerical_hessian(sse_func, best_params)

# For SSE = sum(residuals^2): Hessian(SSE) ~= 2 * J^T J  (Gauss-Newton approx)
JTJ = H / 2.0
JTJ_inv = np.linalg.inv(JTJ)

# Parameter covariance matrix
cov = sigma2 * JTJ_inv
se = np.sqrt(np.diag(cov))

alpha = 0.05
t_crit = stats.t.ppf(1 - alpha / 2, dof)

names = ["k1", "k2", "k3", "k4"]
print(f"dof = {dof}, sigma^2 = {sigma2:.6g}, t_crit (95%) = {t_crit:.4f}\n")
print("95% Confidence Intervals:")
for name, val, s in zip(names, best_params, se):
    lo, hi = val - t_crit * s, val + t_crit * s
    print(f"{name} = {val:.6f}  ±  {t_crit*s:.6f}   (95% CI: [{lo:.6f}, {hi:.6f}])")

print("\nCovariance matrix:")
print(cov)
```

```
dof = 98, sigma^2 = 0.139655, t_crit (95%) = 1.9845

95% Confidence Intervals:
k1 = 0.013443  ±  0.001188   (95% CI: [0.012255, 0.014632])
k2 = 0.099804  ±  0.009208   (95% CI: [0.090596, 0.109011])
k3 = 0.367792  ±  0.113401   (95% CI: [0.254391, 0.481194])
k4 = 0.036354  ±  0.004004   (95% CI: [0.032350, 0.040358])

Covariance matrix:
[[ 3.58625285e-07 -1.20837546e-06 -1.30950124e-05  1.17465050e-06]
 [-1.20837546e-06  2.15279342e-05 -5.03598235e-05 -3.65031179e-06]
 [-1.30950124e-05 -5.03598235e-05  3.26549183e-03 -3.89185704e-05]
 [ 1.17465050e-06 -3.65031179e-06 -3.89185704e-05  4.07014759e-06]]
```

In [58]:

```
# Identifiability analysis of the fitted parameters (k1-k4)
# Two checks:
#  1) Parameter correlation matrix (from the covariance matrix above) -> pairwise identifiability
#  2) Profile likelihood for each parameter

# 1) Correlation matrix
D_ = np.diag(1 / se)
corr = D_ @ cov @ D_
corr_df = pd.DataFrame(corr, index=names, columns=names)
print("Correlation matrix:")
print(corr_df.round(3))
print("\n|corr| > 0.9 flags strong pairwise correlation (practical non-identifiability):")
strong_pairs = []
for i in range(len(names)):
    for j in range(i + 1, len(names)):
        if abs(corr[i, j]) > 0.9:
            strong_pairs.append((names[i], names[j], corr[i, j]))
            print(f"  -> {names[i]} & {names[j]}: corr = {corr[i, j]:.3f}  (STRONGLY CORRELATED)")
if not strong_pairs:
    print("  (none)")

#  2) Profile likelihood
alpha = 0.05
delta_chi2 = stats.chi2.ppf(1 - alpha, df=1)      # 1 parameter of interest at a time
threshold = SSE_min * (1 + delta_chi2 / dof)
print(f"\nProfile-likelihood SSE threshold (95%, 1 dof): {threshold:.4f}  (SSE_min = {SSE_min:.4f})")

def profile_param(idx, span=0.6, npts=13):
    val0 = best_params[idx]
    grid = val0 * np.linspace(1 - span, 1 + span, npts)
    grid = grid[grid > 0]
    sses = []
    for v in grid:
        def obj_fixed(p_other):
            p = np.insert(p_other, idx, v)
            return objective(p, t_exp, df_data)
        p0_other = np.delete(best_params, idx)
        r = minimize(obj_fixed, p0_other, method='Nelder-Mead',
                     options={'xatol': 1e-10, 'fatol': 1e-10, 'maxiter': 2000})
        sses.append(r.fun)
    return grid, np.array(sses)

def lin_interp(x0, y0, x1, y1, ytarget):
    if y1 == y0:
        return x0
    return x0 + (ytarget - y0) * (x1 - x0) / (y1 - y0)

def find_crossings(grid, sses, val0, threshold):
    idx0 = np.argmin(np.abs(grid - val0))
    left_grid, left_sse = grid[:idx0 + 1], sses[:idx0 + 1]
    right_grid, right_sse = grid[idx0:], sses[idx0:]
    lo = hi = None
    for i in range(len(left_grid) - 1, 0, -1):
        if (left_sse[i] - threshold) * (left_sse[i - 1] - threshold) <= 0 and left_sse[i - 1] != left_sse[i]:
            lo = lin_interp(left_grid[i], left_sse[i], left_grid[i - 1], left_sse[i - 1], threshold)
            break
    for i in range(len(right_grid) - 1):
```

```python
        if (right_sse[i] - threshold) * (right_sse[i + 1] - threshold) <= 0 and right_sse[i + 1] != right_sse[i]:
            hi = lin_interp(right_grid[i], right_sse[i], right_grid[i + 1], right_sse[i + 1], threshold)
            break
    return lo, hi

profiles = {}
print("\nRunning profile likelihood scans (re-optimizing 3 parameters at ~13 grid points, 4x)...")
for idx, name in enumerate(names):
    grid, sses = profile_param(idx)
    profiles[name] = (grid, sses)

print("\nProfile-likelihood 95% CIs (vs. linearized CI from the previous cell):")
for idx, (name, val0, s) in enumerate(zip(names, best_params, se)):
    grid, sses = profiles[name]
    lo, hi = find_crossings(grid, sses, val0, threshold)
    lo_s = f"{lo:.5f}" if lo is not None else "< scanned range (flat/unbounded)"
    hi_s = f"{hi:.5f}" if hi is not None else "> scanned range (flat/unbounded)"
    lin_lo, lin_hi = val0 - t_crit * s, val0 + t_crit * s
    print(f"{name}: best={val0:.5f}")
    print(f"    profile CI    ~= [{lo_s}, {hi_s}]")
    print(f"    linearized CI ~= [{lin_lo:.5f}, {lin_hi:.5f}]")

# ---------- Plot the profiles ----------
fig, axes = plt.subplots(1, 4, figsize=(18, 4))
for ax, name in zip(axes, names):
    grid, sses = profiles[name]
    ax.plot(grid, sses, 'o-', color='steelblue')
    ax.axhline(threshold, color='red', linestyle='--', label='95% threshold')
    ax.axvline(best_params[names.index(name)], color='gray', linestyle=':')
    ax.set_title(name)
    ax.set_xlabel(name)
    ax.set_ylabel("SSE")
    ax.legend(fontsize=8)
plt.tight_layout()
plt.show()
```

```
Correlation matrix:
       k1     k2     k3     k4
k1  1.000 -0.435 -0.383  0.972
k2 -0.435  1.000 -0.190 -0.390
k3 -0.383 -0.190  1.000 -0.338
k4  0.972 -0.390 -0.338  1.000

|corr| > 0.9 flags strong pairwise correlation (practical non-identifiability):
  -> k1 & k4: corr = 0.972  (STRONGLY CORRELATED)

Profile-likelihood SSE threshold (95%, 1 dof): 14.2227  (SSE_min = 13.6862)

Running profile likelihood scans (re-optimizing 3 parameters at ~13 grid points, 4x)...

Profile-likelihood 95% CIs (vs. linearized CI from the previous cell):
k1: best=0.01344
    profile CI    ~= [0.01256, 0.01462]
    linearized CI ~= [0.01225, 0.01463]
k2: best=0.09980
    profile CI    ~= [0.09298, 0.10970]
    linearized CI ~= [0.09060, 0.10901]
k3: best=0.36779
    profile CI    ~= [0.28486, 0.52820]
    linearized CI ~= [0.25439, 0.48119]
k4: best=0.03635
    profile CI    ~= [0.03266, 0.04043]
    linearized CI ~= [0.03235, 0.04036]
```

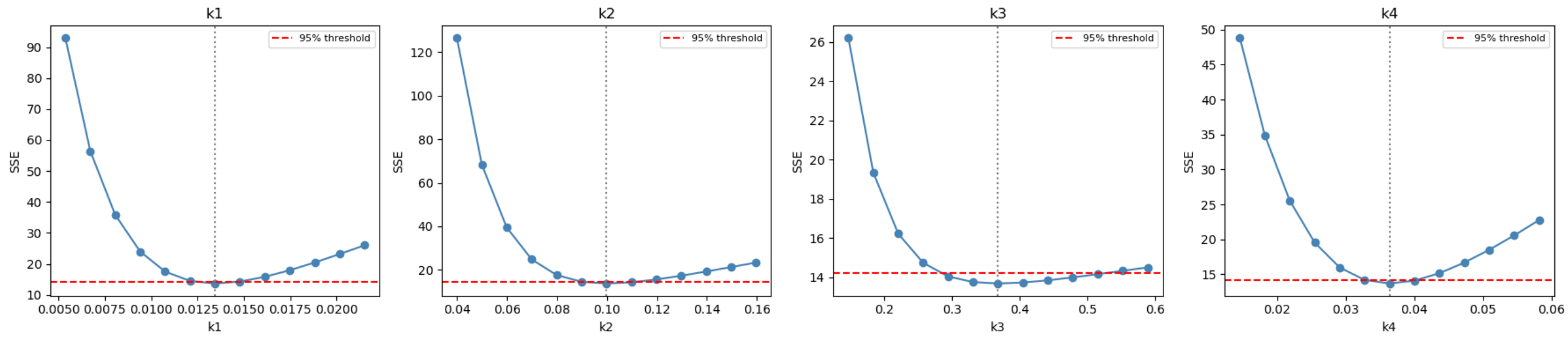

In [59]:

```python
#Residual analysis (r squared for indivivual concentrations and combined)
solution_opt = odeint(model, y0, t_exp, args=(best_k1, best_k2, best_k3, best_k4, A0))

A_fit = solution_opt[:, 0]
B_fit = solution_opt[:, 1]
C_fit = solution_opt[:, 2]
D_fit = solution_opt[:, 3]

res_B = Int1_combined - B_fit #intermediate A residuals
res_C = Int2_combined - C_fit #intermediate B residuals
res_D = Cyclomet - D_fit #cyclometatalated product residuals


rmse_B = np.sqrt(np.mean(res_B**2)) #root mean square errors
rmse_C = np.sqrt(np.mean(res_C**2))
rmse_D = np.sqrt(np.mean(res_D**2))
print(rmse_B, rmse_C,rmse_D)

#individial R squared calculation
ss_res_B = np.sum(res_B**2)
ss_tot_B = np.sum((Int1_combined - np.mean(Int1_combined))**2)
r2_B = 1 - ss_res_B / ss_tot_B
print("R squared Int 1 = ",r2_B)
```

```
ss_res_C = np.sum(res_C**2)
ss_tot_C = np.sum((Int2_combined - np.mean(Int2_combined))**2)
r2_C = 1 - ss_res_C / ss_tot_C
print("R squared Int 2 = ",r2_C)

ss_res_D = np.sum(res_D**2)
ss_tot_D = np.sum((Cyclomet - np.mean(Cyclomet))**2)
r2_D = 1 - ss_res_D / ss_tot_D
print("R squared Cyclomet = ",r2_D)

#R squared combined
ss_res_comb = ss_res_B + ss_res_C + ss_res_D
ss_tot_comb = ss_tot_B + ss_tot_C + ss_tot_D
r2_comb = 1 - ss_res_comb / ss_tot_comb
print("R squared combined = ",r2_comb)
```

```
0.3667622123040644 0.25785977830137735 0.4489192304873175
R squared Int 1 =  0.7918509928058375
R squared Int 2 =  0.28624139289598105
R squared Cyclomet =  0.9841621457487354
R squared combined =  0.9701026246002837
```

In [60]:
```
# Solve ODE with optimized parameters
solution_opt = odeint(model, y0, t_exp, args=(best_k1, best_k2, best_k3, best_k4, A0))


#ax.tick_params(axis='both', which='major', labelsize=14)
#ax.tick_params(axis='both', which='minor', labelsize=8)

A_fit = solution_opt[:, 0]
B_fit = solution_opt[:, 1]
C_fit = solution_opt[:, 2]
D_fit = solution_opt[:, 3]

# Plot results
fig, ax = plt.subplots(figsize=(10, 6))
plt.plot(Time, df_data["A_exp"], 'bo', label=" Free Ligand")
plt.plot(Time, df_data["B_exp"], 'yo', label="Intermed. 1")
plt.plot(Time, df_data["C_exp"], 'ro', label="Intermed. 2")
plt.plot(Time, df_data["D_exp"], 'go', label="Cyclomet.")

#plt.plot(t_exp, A_fit, 'b-', label="A_fit")
plt.plot(t_exp, B_fit, 'y-', label="Intermed. 1 fit")
plt.plot(t_exp, C_fit, 'r-', label="Intermed. 2 fit")
plt.plot(t_exp, D_fit, 'g-', label="Cyclomet. fit")

ax.set_ylim([0, 60])
ax.set_xlim([0, 130])

plt.xlabel("time / min")
plt.ylabel("concentration / mmol/L")
plt.legend()
plt.savefig('PTbisPYA_MeCN_75deg_2int.png')
```

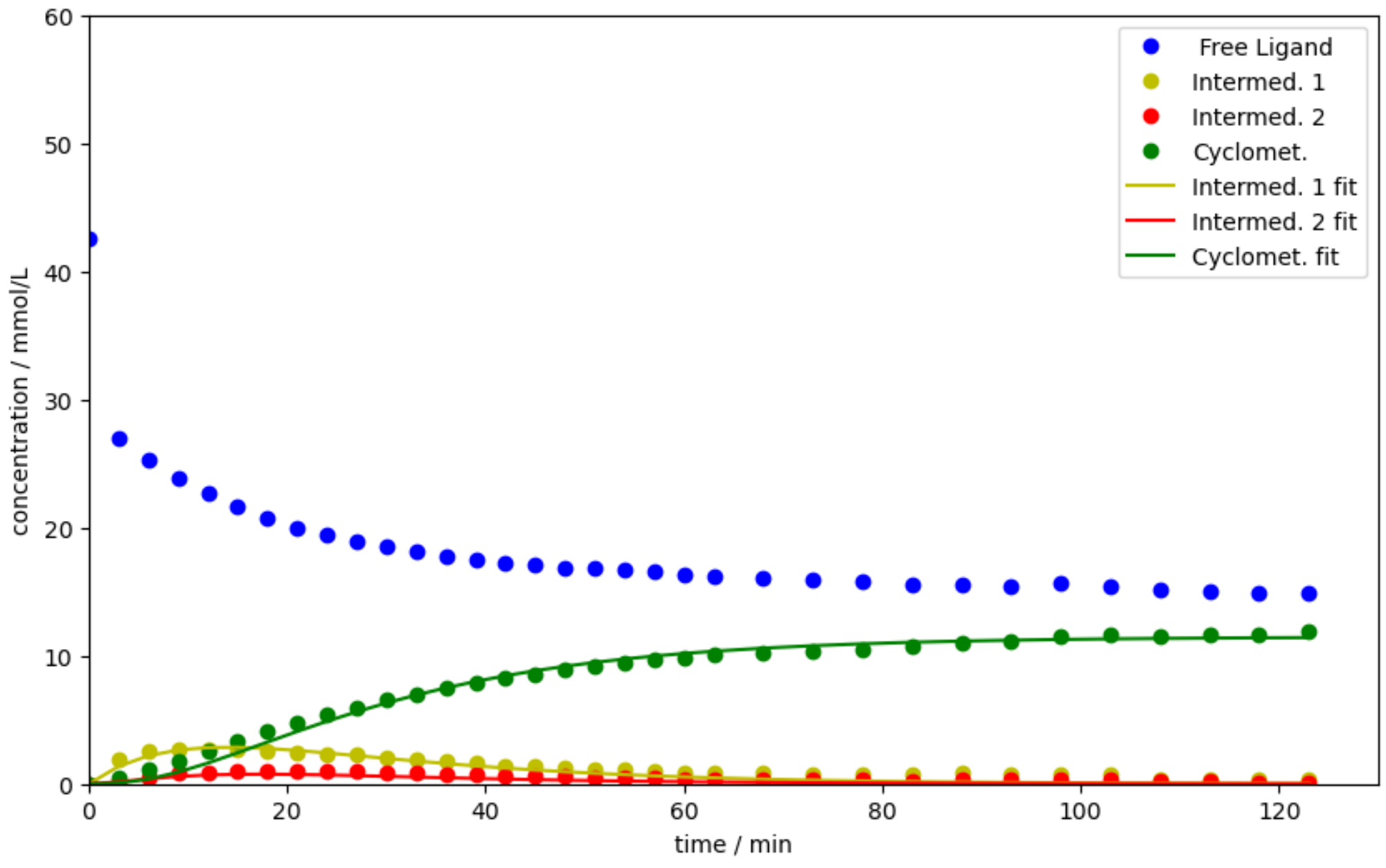

In [67]:
```
# Solve ODE with optimized parameters
solution_opt = odeint(model, y0, t_exp, args=(best_k1, best_k2, best_k3, best_k4, A0))

A_fit = solution_opt[:, 0]
B_fit = solution_opt[:, 1]
C_fit = solution_opt[:, 2]
D_fit = solution_opt[:, 3]


# Plot results
plt.rcParams.update({'font.size': 14})
#ax.tick_params(axis='both', which='major', labelsize=14)
#ax.tick_params(axis='both', which='minor', labelsize=8)
csfont = {'fontname':'Arial'}
```

```python
hfont = {'fontname':'Arial'}



#fig, ax = plt.subplots(figsize=(6, 4))
fig, ax = plt.subplots(figsize=(10, 5))
#plt.plot(Time, df_data["A_exp"], 'bo', label=" Free Ligand")
plt.plot(Time, df_data["B_exp"], 'yo', label="Intermediate A")
plt.plot(Time, df_data["C_exp"], 'ro', label="Intermediate B")
plt.plot(Time, df_data["D_exp"], 'go', label="Complex 2")

#plt.plot(t_exp, A_fit, 'b-', label="A_fit")
plt.plot(t_exp, B_fit, 'y-')
plt.plot(t_exp, C_fit, 'r-')
plt.plot(t_exp, D_fit, 'g-')

ax.set_ylim([0, 13])
ax.set_xlim([0, 120])

plt.xlabel("Time [min]",  **hfont)
plt.ylabel("Concentration  [mmol/L]",  **hfont)
#plt.legend()
plt.savefig('PTbisPYA_MeCN_75deg_fit_no_freeLig.png')
```

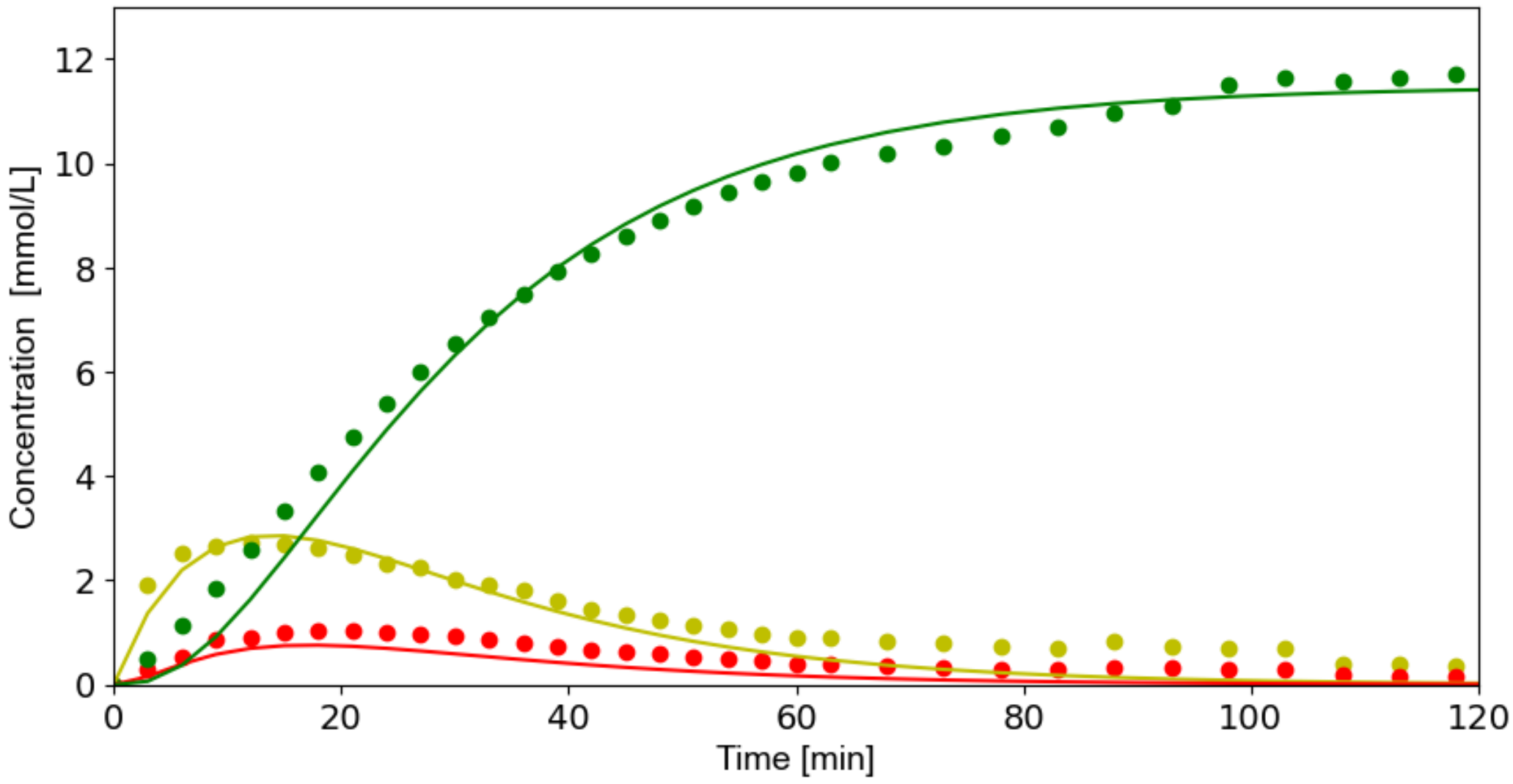

In [ ]:

Kinetic Model Complex 2 Formation 75 deg (Int. 1 as Two Isomers)

In [1]:
```
import numpy as np
import pandas as pd
import matplotlib.pyplot as plt
import math
```

NMR Data

In [3]:
```
#Data arrays, c in mmol/L, first concentration values for t = 0 (in dead time) was fixed at added starting conditions
Time = np.array([0,
3,
6,
9,
12,
15,
18,
21,
24,
27,
30,
33,
36,
39,
42,
45,
48,
51,
54,
57,
60,
63,
68,
73,
78,
83,
88,
93,
98,
103,
108,
113,
118,
123])


Free_Lig = np.array([42.5,
27.03264201,
25.27791983,
23.82243319,
22.62724681,
21.61839678,
20.70383426,
19.97688107,
19.39223704,
18.90188053,
18.53135871,
18.08906776,
17.77517111,
17.45666544,
17.27836197,
17.09795151,
16.86038912,
16.88426826,
16.74924574,
16.54416601,
16.35089417,
16.22825018,
16.07882993,
15.95030394,
15.78257927,
15.58259143,
15.54277822,
15.3842716,
15.63921661,
15.48150011,
15.14332926,
14.97766769,
14.84786873,
14.85159984])

S_3_47 = np.array([0,
0.825445897,
1.004925773,
1.079082813,
1.086378243,
1.06256933,
1.013090334,
0.948010882,
0.860550875,
0.806698133,
0.770329843,
0.726048073,
0.699984687,
0.57576572,
0.519938559,
```

```
0.471918649,
0.434723368,
0.39149509,
0.363834786,
0.320126292,
0.2872275,
0.364422986,
0.32975784,
0.306591556,
0.207845142,
0.193154199,
0.352245499,
0.32119822,
0.308004991,
0.277686377,
0.104180676,
0.101778715,
0.072472243,
0.116416105])


Cyclomet1 = np.array([0,
0.511858277,
1.126050986,
1.847833296,
2.603099084,
3.345512274,
4.086942204,
4.758279817,
5.419811178,
6.008230211,
6.54813595,
7.048184585,
7.498332828,
7.923838142,
8.265555747,
8.61352407,
8.913330262,
9.18407764,
9.439461596,
9.639010491,
9.826532021,
10.02959255,
10.19033784,
10.33870459,
10.53316161,
10.70918252,
10.97782291,
11.10608553,
11.50913392,
11.62738836,
11.55724337,
11.63327036,
11.71078979,
11.84221289])

Cyclomet2 = np.array([0,
0.512653663,
1.124321504,
1.844075842,
2.583223207,
3.328349137,
4.059156359,
4.738017659,
5.386775432,
5.979584015,
6.531701479,
7.029485107,
7.471591696,
7.897167243,
8.246074929,
8.580839489,
8.887607505,
9.170118874,
9.433052855,
9.655251822,
9.826532021,
10.02959255,
10.19033784,
10.33870459,
10.53316161,
10.70918252,
10.97782291,
11.10608553,
11.50913392,
11.62738836,
11.55724337,
11.63327036,
11.71289678,
11.86468738])

S_3_57 = np.array([0,
1.169068566,
1.653244592,
1.610218233,
1.667317486,
1.665201723,
1.626968752,
1.555787826,
1.469375166,
```

```
1.555471779,
1.305223595,
1.235517558,
1.189997935,
1.051288189,
0.950627053,
0.900954917,
0.83859084,
0.772663327,
0.731989766,
0.689067001,
0.652092075,
0.622679463,
0.579415191,
0.560121368,
0.562794603,
0.591856928,
0.610613471,
0.424085735,
0.392713629,
0.399415592,
0.283681623,
0.266106747,
0.267698397,
0.241715779])



S_3_63 = np.array([0,
0.264177101,
0.50740903,
0.973830209,
0.896433682,
0.984654836,
1.033378829,
1.04096397,
1.025258164,
0.990378808,
0.952769153,
0.901472884,
0.794888491,
0.738616229,
0.677794639,
0.629041675,
0.585130384,
0.531843016,
0.500105699,
0.456882689,
0.405577641,
0.387245128,
0.360502241,
0.332675134,
0.306795232,
0.280731846,
0.341135551,
0.313657852,
0.290145672,
0.304029816,
0.180936329,
0.162014739,
0.157101956,
0.138480612])

S_4_21 = np.array([0,
0.309421938,
0.557830901,
0.774086418,
0.922358358,
1.009745498,
1.039673443,
1.0344411,
1.010412709,
0.973285905,
0.913157863,
0.852890235,
0.794888491,
0.738616229,
0.677794639,
0.629041675,
0.585130384,
0.531843016,
0.500105699,
0.456882689,
0.405577641,
0.387245128,
0.360502241,
0.332675134,
0.306795232,
0.280731846,
0.341135551,
0.313657852,
0.290145672,
0.304029816,
0.180936329,
0.162014739,
0.157101956,
0.138480612])

S_4_23 = np.array([0,
```

```
0.825445897,
1.004925773,
1.079082813,
1.086378243,
1.06256933,
1.033545632,
0.958624811,
0.884063933,
0.825854125,
0.745688671,
0.682594172,
0.639877716,
0.57576572,
0.519938559,
0.471918649,
0.434723368,
0.39149509,
0.363834786,
0.320126292,
0.2872275,
0.287733176,
0.259956989,
0.239782622,
0.240620148,
0.219651273,
0.283071475,
0.276279087,
0.272344296,
0.290630279,
0.152688704,
0.137967912,
0.141909727,
0.118033215])


S_4_26 = np.array([0,
1.034915172,
1.371066828,
1.56061633,
1.607558167,
1.596496503,
1.577419524,
1.521654694,
1.424838803,
1.337126836,
1.240188038,
1.155285382,
1.080311887,
1.018436805,
0.916160315,
0.863127541,
0.79881187,
0.737010532,
0.688858059,
0.63660838,
0.584412254,
0.557494662,
0.5134868,
0.479844419,
0.455270846,
0.412998612,
0.454677379,
0.424085735,
0.392713629,
0.399415592,
0.283681623,
0.266106747,
0.267698397,
0.241715779])
```

In [ ]:

In [5]:
```
#Combined concentrations ( with similar species) in mmol/L

Cyclomet = (Cyclomet1 + Cyclomet2) / 2
Int1_combined = (S_4_26 + S_4_23 + S_3_57 + S_3_47) / 2
Int2_combined = (S_4_21 + S_3_63) / 2
Free_Lig_corr = Free_Lig

#Combined concentrations ( with similar species, only same conc)

#Int1_combined_V2 = S_4_26 + S_3_57
#Int2_combined_V2 = S_4_23 + S_3_47
Int2_combined_V2 = (S_4_26 + S_3_57) / 2
Int1_combined_V2 = (S_4_23 + S_3_47) / 2


Int3_combined_V2 = (S_4_21 + S_3_63) /2
```

Free Lig (A) -> Int1 (B) + Int1' (C) -> Int2 (D) -> cyclomet (E)

In [ ]:

In [11]:
```
#same as before but without Free Lig with no A + another A side reaction
import numpy as np
import matplotlib.pyplot as plt
from scipy.integrate import odeint
```

```python
import ipywidgets as widgets
from ipywidgets import interactive

# Define the system of ODEs
def model(y, t, k1, k2, k3, k4, k5, A0):
    A, B, C, D, E = y      ### Change here

    # Rate equations
    dA_dt = -k1 * A -k2 * A -k5 * A
    dB_dt = k1 * A -k3 * B
    dC_dt = k2 * A -k3 * C
    dD_dt = k3 * C + k3 * B - k4 * D
    dE_dt = k4 * D

    return [dA_dt, dB_dt, dC_dt, dD_dt, dE_dt] ### Change here

# Initial conditions: [A0, B0, C0, D0]
A0 = 42.5  # Initial concentration of A = 42.5 mmol/L (max) - all species recorded
B0 = 0   # Initial concentration of B
C0 = 0  # Initial concentration of C
D0 = 0   # Initial concentration of D
E0 = 0    ### Change here

# Time points where solution is computed
t = np.linspace(0, 120, 500)  # from t=0 to t=50, with 500 time points

# Initial values vector
y0 = [A0, B0, C0, D0, E0]    ### Change here
```

Optimization of the variables

In [13]:
```python
from scipy.optimize import minimize

def objective(params, t_exp, data):
    k1, k2, k3, k4, k5 = params

    # Solve ODE system with current parameters
    solution = odeint(model, y0, t_exp, args=(k1, k2, k3, k4, k5, A0))

    #A_t = solution[:, 0]
    B_t = solution[:, 1]
    C_t = solution[:, 2]
    D_t = solution[:, 3]
    E_t = solution[:, 4]

    # Experimental data
    A_exp = Free_Lig_corr
    B_exp = Int1_combined_V2
    C_exp = Int2_combined_V2
    D_exp = Int3_combined_V2
    E_exp = Cyclomet

    # Compute sum of squared errors (SSE)
    sse = np.sum((C_exp - C_t)**2 + (D_exp - D_t)**2 + (B_exp - B_t)**2 + (E_exp - E_t)**2)
    # sse = np.sum((A_exp - A_t)**2 + (C_exp - C_t)**2 + (D_exp - D_t)**2)

    return sse
```

In [16]:
```python
# Experimental time points
t_exp = Time

# Initial guess for k1, k2, k3, k4
initial_guess = [0.001, 0.001, 0.002, 0.02, 0.02]
df_data = pd.DataFrame ({"Time (h)":Time, "A_exp": Free_Lig_corr, "B_exp": Int1_combined_V2, "C_exp": Int2_combined_V2,"D_exp": Int3_combined_V2,


# Run optimization
result = minimize(objective, initial_guess, args=(t_exp, df_data), method='Nelder-Mead')


SSE_min = result.fun
best_k1, best_k2, best_k3, best_k4, best_k5 = result.x
print("Optimized k1 =", best_k1)
print("Optimized k2 =", best_k2)
print("Optimized k3 =", best_k3)
print("Optimized k4 =", best_k4)
print("Optimized k5 =", best_k5)
print ("SSE min =", SSE_min)
```

```
Optimized k1 = 0.004974332620281321
Optimized k2 = 0.008422741247257202
Optimized k3 = 0.10517773127623177
Optimized k4 = 0.3466902334371947
Optimized k5 = 0.036417999811252905
SSE min = 11.583324911616293
```

In [23]:
```python
# Solve ODE with optimized parameters
solution_opt = odeint(model, y0, t_exp, args=(best_k1, best_k2, best_k3, best_k4, best_k5, A0))

A_fit = solution_opt[:, 0]
B_fit = solution_opt[:, 1]
C_fit = solution_opt[:, 2]
D_fit = solution_opt[:, 3]
E_fit = solution_opt[:, 4]

# Plot results
fig, ax = plt.subplots(figsize=(10, 6))
plt.plot(Time, df_data["A_exp"], 'bo', label=" Free Ligand")
plt.plot(Time, df_data["B_exp"], 'yo', label="Intermed. 1")
plt.plot(Time, df_data["C_exp"], 'ro', label="Intermed. 2")
```

```
plt.plot(Time, df_data["D_exp"], 'bo', label="Intermed. 3")
plt.plot(Time, df_data["E_exp"], 'go', label="Cyclomet.")

#plt.plot(t_exp, A_fit, 'b-', label="A_fit")
plt.plot(t_exp, B_fit, 'y-', label="Intermed. 1 fit")
plt.plot(t_exp, C_fit, 'r-', label="Intermed. 2 fit")
plt.plot(t_exp, D_fit, 'b-', label="Intermed. 3 fit")
plt.plot(t_exp, E_fit, 'g-', label="Cyclomet. fit")

ax.set_ylim([0, 50])
ax.set_xlim([0, 120])

plt.xlabel("time / min")
plt.ylabel("concentration / mmol/L")
plt.legend()
plt.savefig('PTbisPYA_MeCN_fit_75_3intem.pdf')
plt.savefig('PTbisPYA-MeCN_fit_75_3intem.jpg')
```

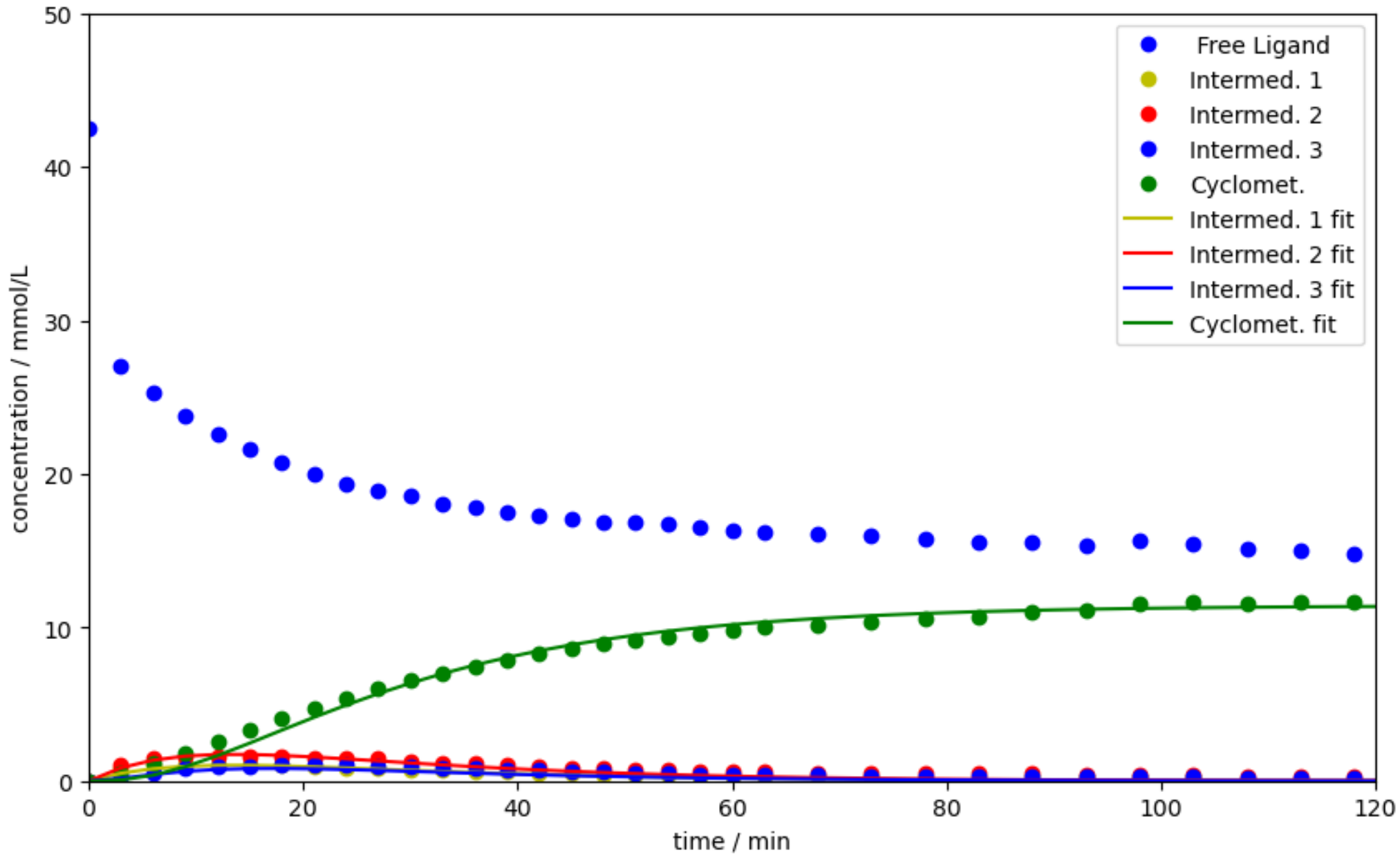

In [32]:
```
# Plot results
#fig, ax = plt.subplots(figsize=(6, 4))
fig, ax = plt.subplots(figsize=(10, 5))
#plt.plot(Time, df_data["A_exp"], 'bo', label=" Free Ligand")
plt.plot(Time, df_data["B_exp"], 'yo', label="Intermed. 1")
plt.plot(Time, df_data["C_exp"], 'bo', label="Intermed. 2")
plt.plot(Time, df_data["D_exp"], 'ro', label="Intermed. 3")
plt.plot(Time, df_data["E_exp"], 'go', label="Cyclomet.")

#plt.plot(t_exp, A_fit, 'b-', label="A_fit")
plt.plot(t_exp, B_fit, 'y-', label="Intermed. 1 fit")
plt.plot(t_exp, C_fit, 'b-', label="Intermed. 2 fit")
plt.plot(t_exp, D_fit, 'r-', label="Intermed. 3 fit")
plt.plot(t_exp, E_fit, 'g-', label="Cyclomet. fit")
ax.set_ylim([0, 13])
ax.set_xlim([0, 120])

plt.xlabel("Time [min]")
plt.ylabel("Concentration  [mmol/L]")
#plt.legend()
plt.savefig('PTbisPYA_MeCN_fit75_3int.pdf')
plt.savefig('PTbisPYA-MeCN_fit75_3int.jpg')
```

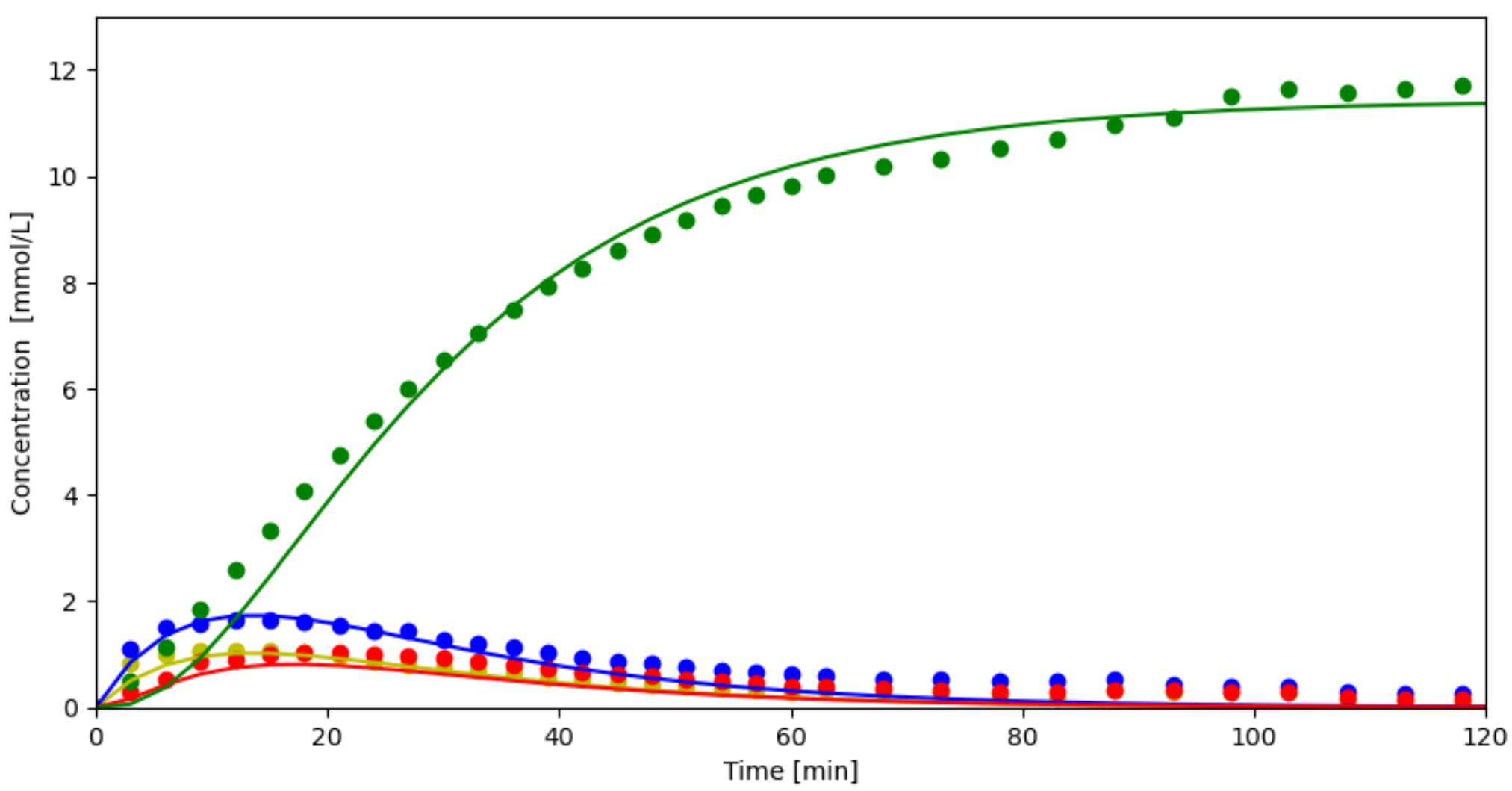

In [ ]:

In [29]:
```
#Residual analysis (r squared for indivivual concentrations and combined)
solution_opt = odeint(model, y0, t_exp, args=(best_k1, best_k2, best_k3, best_k4, best_k5, A0))

A_fit = solution_opt[:, 0]
B_fit = solution_opt[:, 1]
C_fit = solution_opt[:, 2]
D_fit = solution_opt[:, 3]
E_fit = solution_opt[:, 4]

res_B = Int1_combined_V2 - B_fit #intermediate A isomer 2 residuals
res_C = Int2_combined_V2 - C_fit #intermediate A isomer 1 residuals
res_D = Int3_combined_V2 - D_fit #intermdiate B  residuals
res_E = Cyclomet - E_fit #cyclometalated product residuals


rmse_B = np.sqrt(np.mean(res_B**2)) #root mean square errors
rmse_C = np.sqrt(np.mean(res_C**2))
rmse_D = np.sqrt(np.mean(res_D**2))
rmse_E = np.sqrt(np.mean(res_E**2))
print(rmse_B, rmse_C,rmse_D,rmse_E)

#individial R squared calculation
ss_res_B = np.sum(res_B**2)
ss_tot_B = np.sum((Int1_combined_V2 - np.mean(Int1_combined_V2))**2)
r2_B = 1 - ss_res_B / ss_tot_B
print("R squared Int 1 = ",r2_B)

ss_res_C = np.sum(res_C**2)
ss_tot_C = np.sum((Int2_combined_V2 - np.mean(Int2_combined_V2))**2)
r2_C = 1 - ss_res_C / ss_tot_C
print("R squared Int 2 = ",r2_C)

ss_res_D = np.sum(res_D**2)
ss_tot_D = np.sum((Int3_combined_V2 - np.mean(Int3_combined_V2))**2)
r2_D = 1 - ss_res_D / ss_tot_D
print("R squared Int 3 = ",r2_D)

ss_res_E = np.sum(res_E**2)
ss_tot_E = np.sum((Cyclomet - np.mean(Cyclomet))**2)
r2_D = 1 - ss_res_E / ss_tot_E
print("R squared Cyclomet = ",r2_D)


#R squared combined
ss_res_comb = ss_res_B + ss_res_C + ss_res_D + ss_res_E
ss_tot_comb = ss_tot_B + ss_tot_C + ss_tot_D + ss_tot_E
r2_comb = 1 - ss_res_comb / ss_tot_comb
print("R squared combined = ",r2_comb)
```

```
0.14722027352660297 0.2565079684490591 0.23996544590489052 0.4423035889169335
R squared Int 1 =  0.796057946879247
R squared Int 2 =  0.7152907617791854
R squared Int 3 =  0.38186751555297416
R squared Cyclomet =  0.9846255053727951
R squared combined =  0.9741021891281791
```

In [ ]:

In [ ]:
